# Nutritional status of school‐age children (5–19 years) in South Asia: A scoping review

**DOI:** 10.1111/mcn.13607

**Published:** 2023-12-14

**Authors:** Tashi Choedon, Eilise Brennan, William Joe, Natasha Lelijveld, Oliver Huse, Christina Zorbas, Kathryn Backholer, Zivai Murira, Stephanie V. Wrottesley, Vani Sethi

**Affiliations:** ^1^ Institute of Economic Growth (IEG) Delhi India; ^2^ Emergency Nutrition Network (ENN) Kiddlington Oxford UK; ^3^ Global Centre for Preventive Health and Nutrition (GLOBE), Institute for Health Transformation, Faculty of Health Deakin University Geelong Victoria Australia; ^4^ UNICEF South Asia Regional Office Kathmandu Nepal

**Keywords:** adolescents, micronutrients, nutrition, school‐age children, South Asia

## Abstract

Information on malnutrition for school‐age children and adolescents (5–19 years) in South Asia is fragmented and inconsistent, which limits the prioritization of nutrition policies, programmes and research for this age group. This scoping review aimed to synthesize existing evidence on the burden of malnutrition for children and adolescents aged 5–19 years in South Asia, and on interventions to improve their nutritional status. Cochrane Library, EMBASE, Medline and Google Scholar were systematically searched for articles published between January 2016 and November 2022. Eligible studies reported the prevalence of undernutrition, overweight/obesity, micronutrient deficiencies and unhealthy dietary intakes, and interventions that aimed to address these in South Asia. In total, 296 articles met our inclusion criteria. Evidence revealed widespread, yet heterogeneous, prevalence of undernutrition among South Asian children and adolescents: thinness (1.9%–88.8%), wasting (3%–48%), underweight (9.5%–84.4%) and stunting (3.7%–71.7%). A triple burden of malnutrition was evident: the prevalence of overweight and obesity ranged from 0.2% to 73% and 0% to 38% (with rapidly rising trends), respectively, alongside persistent micronutrient deficiencies. Diets often failed to meet nutritional requirements and high levels of fast‐food consumption were reported. Education, fortification, supplementation and school feeding programmes demonstrated beneficial effects on nutritional status. Comprehensive and regular monitoring of all forms of malnutrition among children and adolescents, across all countries in South Asia is required. Further, more large‐scale intervention research is needed to ensure policy and programmes effectively target and address malnutrition among children and adolescents in South Asia.

## Key messages

- A triple burden of malnutrition (undernutrition, overweight/obesity and micronutrient deficiencies) is affecting children and adolescents (5–19 years) in South Asia, with heterogeneity within, and between, countries.
- In most countries, nationally representative data on the nutritional status of children and adolescents is absent and should be prioritized.
- Interventions targeting children and adolescents are primarily limited to small‐scale trials and focused predominantly on undernutrition and micronutrient deficiencies despite rapidly rising rates of overweight and obesity.
- Further research is needed to inform the scalability and sustainability of nutrition interventions across different contexts and to understand the effectiveness of policies and programmes that address overnutrition.

## Graphical abstract

Information on malnutrition for school‐age children and adolescents in South Asia is fragmented and inconsistent, limiting the prioritization of nutrition policies, programmes and research. This scoping review synthesized existing evidence on the burden of malnutrition for children and adolescents in South Asia, and on interventions to improve their nutritional status.

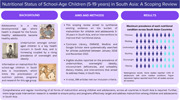

## 1 INTRODUCTION

Middle childhood (5–9 years) and adolescence (10–19 years) are critical phases of growth and development, during which time health outcomes impact current and future generations (Aguayo & Paintal, 2017; Norris et al., 2022). Rapid growth during this time creates high nutrient demand and can lead to growth faltering if nutritional needs are not met (Barker et al., 2021). In low‐ and middle‐income countries (LMICs), approximately 12 million girls aged 15–19 years give birth each year (Sully et al., 2020), further predisposing them to nutritional disadvantage, and increasing their risk of maternal mortality and morbidity, as well as poor birth outcomes (Fall, 2013; Sully et al., 2020). The adolescent period is also characterized by increased independence and exposure to social and environmental factors that shape diet and physical activity behaviours across the life‐course (Barker et al., 2021). Rapid urbanization has increased access to, and marketing of, highly processed foods high in salt, sugar and fat in LMICs, with the consumption of these foods also associated with a triple burden of undernutrition, overweight/obesity and micronutrient deficiencies in childhood and adolescence.

The United Nations (UN) Sustainable Development Goal (SDG) 2, which calls for an end to all forms of malnutrition by 2030, cannot be achieved without targeting school‐age children and adolescents. School‐age children and adolescents (5–19 years) form a quarter (1.97 billion) of the global population, with 28.1% (552 million) of them residing in South Asia (United Nations, Department of Economic and Social Affairs, Population Division, 2022). South Asia is the epicentre for the global nutrition crises for women and children, with 28% of children born at a low birth weight and 38% of children under 5 being stunted (Aguayo & Paintal, 2017). Despite the potential for multiple benefits to current and future generations, school‐age children and adolescents remain inconsistently considered across most of the region's nutrition programming, policy and research agendas (Akseer et al., 2017; Norris et al., 2022). For example, data for adolescents are often omitted from regional and national reports, with nationally representative household surveys focusing on children under‐five, pregnant women and lactating mothers (Ties Boerma & Sommerfelt, 1993). This limits our understanding of the burden of malnutrition in the region as well as of effective interventions to optimize nutrition in this critical period of life.

This review aimed to summarize existing research on the nutritional status of school‐age children and adolescents (5–19 years of age) in South Asia, as well as on interventions targeting malnutrition in this age group.

## 2 METHODS

### 2.1 Search strategy

Electronic databases (Cochrane Library, EMBASE and MEDLINE) and grey literature (Google Advanced Search, first 208 results) were searched to identify relevant studies. The databases were searched for studies published from January 2016 to November 2022. The search was limited to studies published after 2016 to ensure the findings reflected contemporary data to increase the relevance of findings to policy and practice. The review was conducted according to PRISMA guidelines for scoping reviews. The scope of the review was defined by the population, interventions, comparison, outcome (PICO) framework and the inclusion and exclusion criteria are presented in Table 1. The search terms are summarized in Supporting Information: Tables S1–S3.

**Table 1:** PICO framework.

Population	Intervention	Comparison	Outcome
Adolescents and school‐age children aged 5–19 years living in South Asia South Asia as defined by UNICEF working areas—Afghanistan, Maldives, Bangladesh, Nepal, Bhutan, Pakistan, India and Sri Lanka	None required: when identifying data on nutritional status no intervention was required. For intervention studies, any intervention designed to improve nutritional status was eligible for inclusion. Both effective and ineffective interventions were eligible for inclusion Intervention outcomes were required to focus on one or more indicators of malnutrition (rather than process evaluations or similar).	None required	Prevalence of: Stunting, thinness, underweight. The following micronutrient deficiencies (anaemia, iron deficiency, iodine deficiency, vitamin A deficiency, vitamin D deficiency, vitamin C deficiency, zinc deficiency). Overweight and obesity. Dietary patterns and behaviours
			Drivers or predictors of: Stunting, thinness, underweight. The following micronutrient deficiencies (anaemia, iron deficiency, iodine deficiency, vitamin A deficiency, vitamin D deficiency, vitamin C deficiency, zinc deficiency). Overweight and obesity. Dietary patterns and behaviours
Additional inclusion criteria	Peer‐reviewed English human studies published between January 2016 to November 2022 No restrictions on study design.		
Additional exclusion criteria	Studies where subjects were pregnant, had any form of disability or were diagnosed with diseases were not eligible for inclusion. All studies that utilized self‐reported anthropometry or had sample sizes less than 100.		

### 2.2 Screening and data extraction

The search results were imported into Endnote X9 and all duplicates were removed. The results were screened for eligibility based on their title and abstract. Full texts of the remaining results were then screened for eligibility according to the inclusion and exclusion criteria. The following data were extracted for all relevant articles: author, date, country in which the research took place, year of data collection, target population (including the age of subjects, sex and sample size), outcomes of interest, classification criteria used and key findings from the literature (Supporting Information: Table S4). To provide a comparable value, national representative Demographic and Healthy Survey data were also searched for and extracted for all countries and variables of interest.

### 2.3 Analysis

Data were synthesized in a narrative format. Results are presented by outcome of interest (stunting, wasting, underweight and thinness, overweight and obesity, micronutrient deficiencies, and dietary patterns and quality. Within each outcome, prevalence and trends studies are presented separately from intervention studies. Demographic and Health Survey data are also presented, where available.

## 3 RESULTS

**Figure 1:**
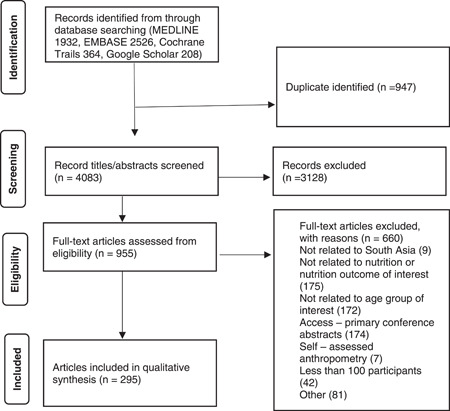
PRISMA flow chart for nutritional status of school‐age children in South Asia, 2016–2022.

**Figure 2:**
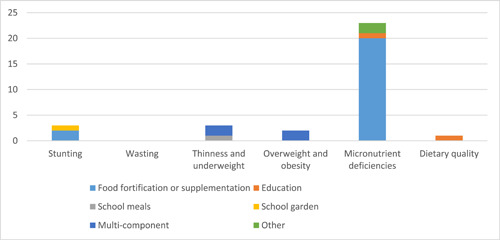
Intervention studies by nutritional outcome of interest.

The initial search revealed 5030 articles. A total of 955 full texts were assessed, of which 295 were eligible for this review (Figure 1). Out of those eligible studies, 174 studies (54%) were from India, 40 studies (12%) were from Pakistan, 38 (12%) were from Bangladesh, 34 (11%) from Nepal, 22 (7%) from Sri Lanka, 6 from Afghanistan, 2 each from Maldives and Bhutan and 4 studies reported prevalence estimates from multiple countries based on pooled data (Supporting Information: Tables S5 and S6). A total of 272 studies reported on the prevalence of, or trends in, malnutrition in one or more countries of interest (Supporting Information: Table S5), while 25 studies reported on the outcomes from one or more interventions aimed at addressing malnutrition in one or more countries of interest (Figure 2 and Supporting Information: Table S6). Of the 25 intervention studies, 22 considered interventions that lasted for 12 months or less. Nationally representative Demographic and Health Survey data were available for Bangladesh, Bhutan, India, Maldives, Nepal, Pakistan and Sri Lanka, for some variables of interest (Table 2). One hundred and ninety‐three included studies were published before the year 2020, while 103 studies were published in the year 2020 or later. Most studies utilized internationally recognized definitions, though some studies used wider or context‐specific definitions (Supporting Information: Table S7).

**Table 2:** Nutritional indicators of adolescents and school‐age girls based on national reports.

Indicator	Afghanistan	Bangladesh	Bhutan	India	Maldives	Nepal	Pakistan	Sri Lanka
Stunting	‐	‐	‐	21.9%^a^	‐	‐	‐	‐
Underweight	‐	‐	‐	35.2%^a^	‐	‐	11.9%^b^	‐
Thinness	‐	24.3%^c^	‐	39.7%	37.5%	30.3%	18.6%^c^	22.9%^c^
Overweight	‐	10.7%^c^	‐	4.2%	11.1%^c^	2.9%^c^	21%^c^	15.8%^c^
Obesity	‐	0.9%^c^	‐	1.2%	5.1%^c^	0.4%^c^	3.7%^c^	5.2%^c^
Anaemia	‐	‐	31.3%^c^	59.1%	60%^c^	43.6^c^	54.7%^b^	‐
Iron deficiency	‐	‐	‐	31%^b^	‐	‐	‐	‐
Iodine deficiency	‐	‐	‐	5.4%^b^, ^d^	‐	‐	‐	‐
Zinc deficiency	‐	‐	‐	28.4%^b^	‐	‐	‐	‐
Vitamin A deficiency	‐	‐	‐	15.5%^b^	‐	‐	‐	‐
Vitamin D deficiency	‐	‐	‐	34.3%	‐	‐	‐	‐

*Note*: ‐, not available.

^a^ Girls aged 5–9 years.

^b^ Adolescent girls aged 10–19 years.

^c^ Adolescent girls 15‐19 years.

^d^ Urinary Iodine Concentration (UIC).

*Source*: Based on national survey reports of the Bangladesh‐DHS 2017–2018, Bhutan‐NNS 2015, India‐NFHS 2019–2021 and CNNS 2016, Maldives‐DHS 2016–2017, Nepal‐DHS 2016, Pakistan‐DHS 2017–2018 and NNS 2018 and Sri Lanka‐DHS 2016.

### 3.1 Stunting

Nationally representative Demographic and Health Survey data on stunting was available for girls aged 5‐9 years in India, with the reported value being 21.9% (Table 2). The prevalence of stunting was recorded in 64 studies, across Bangladesh (*n* = 6), India (*n* = 31), Nepal (*n* = 3), Pakistan (*n* = 11) and Sri Lanka (*n* = 6) and ranged from 3.7% in Sri Lanka (Warnakulasuriya et al., 2019) to 71.7% in Pakistan (Zainab & Kadir, 2016; Supporting Information: Table S5). Fourteen studies reported that the prevalence of stunting increased with age; however, two studies from India (Schott et al., 2019; Verma et al., 2020) and one from Pakistan (Saeedullah et al., 2021) found a higher prevalence among younger adolescents. Studies reported mixed results on gender disparities in stunting, with 4 and 12 studies highlighting higher prevalence in girls and boys, respectively. Other variations in stunting prevalence were observed across household wealth status, social group affiliations, maternal education, adolescent nutrition awareness, paternal occupation, rural residence, access to safe drinking water and physical inactivity (Schott et al., 2019; van Tuijl et al., 2021).

One study conducted in Ethiopia, India, Peru and Vietnam (Gausman et al., 2019) assessed catch‐up growth, finding that 28.7% of children in India who were stunted at 5 years of age in 2006 remained stunted at 15 years of age in 2016. However, 30.4% of all surveyed children in India who were stunted at 5 years of age recovered and were no longer stunted by 8 years of age, and 36.9% of children who were stunted at 12 years of age recovered and were no longer stunted by 15 years of age.

#### 3.1.1 Stunting intervention studies

Three intervention studies in India and one study in Nepal were identified (Figure 2 and Supporting Information: Table S6). The three studies in India focused on providing supplementary nutritious or fortified food to children, while the study in Nepal evaluated a school garden programme. The three food provision studies in India showed mixed impacts on stunting. The first study (Anitha et al., 2019) found that substituting rice for millet in school meals was associated with a significant reduction in stunting (*p* = 0.000) among 136 students at two intervention schools as compared to 107 students at two control schools. The second Indian study (Devara & Deshmukh, 2017) observed reductions in stunting in both the intervention (who received nutritious meals through centralized kitchens) and control groups (who received meals through local kitchens according to Government guidelines), with no significant differences between the groups. The third study in India found feeding micronutrient‐fortified, high‐quality legume protein‐based food products to 100 moderately stunted primary school children did not impact stunting, though the intervention was only implemented for 1 month (Varkey et al., 2020). Similarly, a school garden programme in Nepal did not find an effect on stunting among 172 children, though it positively influenced nutrition knowledge and practices among participants (Shrestha et al., 2020).

### 3.2 Wasting

A total of nine studies presented prevalence estimates on wasting (Supporting Information: Table S5). The prevalence of wasting across studies from Bangladesh (Kamruzzaman et al., 2021; Khanam & Haque, 2021), India (Ramkumar et al., 2018;  Veereshwar Rao et al., 2021; Yang et al., 2020), Pakistan (Iqbal et al., 2020; Jan et al., 2021; Khan et al., 2022) and Sri Lanka (Warnakulasuriya et al., 2019) ranged from 3% (Veereshwar Rao et al., 2021) to 48% (Khanam & Haque, 2021). Six studies (Jan et al., 2021; Kamruzzaman et al., 2021; Khan et al., 2022; Khanam & Haque, 2021; Ramkumar et al., 2018; Veereshwar Rao et al., 2021) reported sex differences in wasting prevalence, with four (Jan et al., 2021; Kamruzzaman et al., 2021; Khan et al., 2022; Khanam & Haque, 2021) studies finding higher prevalence of wasting among girls.

### 3.3 Underweight and thinness

Nationally representative Demographic and Health Survey data on underweight was available for girls aged 5–9 years in India (35.2%) and girls aged 10–19 years in Pakistan (11.9%) (Table 2). Nationally representative Demographic and Health Survey data on underweight was available for girls aged 15–19 years in Bangladesh, India, Maldives, Nepal, Pakistan and Sri Lanka (Table 2). The prevalence ranged from 18.6% (Pakistan) to 39.7% (India). The prevalence of and/or trends in thinness was reported in 121 studies conducted across all eight included South Asian countries, while the prevalence of and/or trends in underweight were reported by 35 studies conducted across Bangladesh, India, Nepal, Pakistan and Sri Lanka (Supporting Information: Table S5). However, the growth references used to define these anthropometric indicators varied between studies. Fifteen studies analysed nationally representative surveys and found thinness ranging from 7.2% (Nepal) to 48.8% (Sri Lanka); however, some surveys used age‐specific cut‐offs, while others utilized adult body mass index (BMI) cut‐offs. Eight national representative studies, conducted in India, Bangladesh, Pakistan, Nepal, and regionally, presented data on the change in the prevalence of thinness over time, with all showing some evidence of a decreasing trend. One nationally representative study (Yang et al., 2020) found that the prevalence of underweight decreased in India from 2012 to 2018. Across all studies, whether nationally representative or not, the prevalence of thinness ranged from 1.9% in Pakistan (Asif et al., 2020) to 88.8% in India (Parray et al., 2016), while the prevalence of underweight ranged from 9.5% in Bangladesh (Ghosh et al., 2021) to 84.4% in India (Verma et al., 2020).

Underweight was found to be more prevalent among boys compared to girls in four studies (Dhobi & Giri, 2021; Pant & Paudel, 2021; Veereshwar Rao et al., 2021; Zareen, 2021), and among girls compared to boys in two studies (Khan et al., 2022; Kunnath et al., 2022). No difference in underweight by gender was reported in one study (Verma et al., 2020). Underweight was reported as being greater among rural populations in three studies, reporting data from India, Pakistan and Bangladesh (Bellundagi et al., 2022; Khan et al., 2022; Wolf et al., 2020), compared to urban populations, while just one study, conducted in India (Tripathi & Chakravarti, 2022), reported a higher prevalence of underweight among urban, compared to rural populations.

Thinness was more prevalent in younger age groups across 13 studies, out of 17 studies which looked at differences by age. A higher prevalence of thinness was found in boys in 17 studies, compared to two which found a higher prevalence in girls (Gamage & Jayawardana, 2017; Hassan et al., 2020). Five studies reported a higher prevalence of thinness in rural areas compared to urban areas across India and Pakistan, although two additional studies from India (Choudhuri & Balaram, 2020; Sutradhar & Choudhuri, 2017) did not find any significant differences. Family size, household income, maternal occupation and education, belonging to religious minorities and lower social class, low dietary diversity, water sanitation and hygiene practices, paternal education, physical inactivity and a history of illness were also reported as predictors of thinness (Radhika et al., 2018; Shinsugi et al., 2019; Young et al., 2020; van Tuijl et al., 2021). Two studies reported on the influence of perceptions and social norms; one study found that underweight adolescents underestimate their weight, the other reported that girls aspire to smaller body sizes, while boys aspire to larger body sizes (Acharya et al., 2016; Goonapienuwala et al., 2019).

#### 3.3.1 Underweight and thinness intervention studies

Three studies reported interventions targeting thinness in school children, two in India and one in Nepal (Figure 2 and Supporting Information: Table S6). One of these studies evaluated a school meals programme (Devara & Deshmukh, 2017), while two evaluated multi‐component interventions (Muhammad et al., 2019; Shrestha et al., 2020). The school midday‐meal programme (India) saw a reduction in thinness among both the control and interventions groups (Devara & Deshmukh, 2017). One of the multi‐component interventions, which comprised of 1 year of school‐health screening, counselling, health education and peer‐led sessions in India saw a reduction in thinness (Muhammad et al., 2019), while the other multi‐component intervention (school gardens, education and water and sanitation strategies) in Nepal found no significant effect on the prevalence of thinness (Shrestha et al., 2020).

### 3.4 Overweight and obesity

Nationally representative Demographic and Health Survey data on overweight and obesity was available for girls aged 15–19 years in Bangladesh, India, Maldives, Nepal, Pakistan and Sri Lanka (Table 2). The prevalence of overweight ranged from 2.9% (Nepal) and 21% (Pakistan). The prevalence of obesity ranged from 0.4% (Nepal) to 5.2% (Sri Lanka). Overweight and/or obesity was reported in 155 studies across all eight South Asian countries (Supporting Information: Table S5). Of those, 104 studies reported on the prevalence of overweight and obesity separately. The prevalence of overweight ranged from 0.2% (India in 2017) (Kujur et al., 2020) to 73% (India in 2015) (Pawar, 2016) and the prevalence of obesity ranged from 0% in India in 2016 (Pawar, 2016; Savanur et al., 2017) and Pakistan in 2016 (Kapoor et al., 2018) to 38% (India in 2015) (K. J. Kumar et al. 2017). However, all studies that reported an obesity prevalence of 0% were conducted among nonrepresentative samples, such as tribal communities. Fifty‐one studies reported on the combined prevalence of overweight and obesity, which ranged from 0.1% (Verma et al., 2020) to 32% (Wolf et al., 2020), with both datapoints coming from studies in India. Of the 155 studies, 12 presented data on the national prevalence of overweight and/or obesity across Bangladesh, India, Maldives, Nepal, Pakistan and Sri Lanka. Among studies that presented data on overweight, this ranged from 3% (Pakistan in 2013) to 25.3% (Afghanistan in 2013) (Harding et al., 2019). Among studies that presented data on obesity, this ranged from 0.1% (Pakistan in 2013) (Harding et al., 2019) to 5.2% (Department of Census and Statistics (DCS) and Ministry of Health, Nutrition and Indigenous Medicine, 2017). Two studies analysed the change in prevalence of overweight over time, with a pooled analysis finding that South Asia has had the highest increase in the number of obese adolescents since the 1970s relative to other global regions (NCD Risk Factor Collaboration (NCD‐RisC), 2017). Two studies (Caleyachetty et al., 2018; Pal et al., 2017) reported on concurrent forms of malnutrition, with one study reporting concurrent stunting and overweight among 2% of adolescents in India in 2007 and among almost 1% of adolescents in Pakistan in 2009 and Sri Lanka in 2008 (Caleyachetty et al., 2018).

Twenty‐one studies explored differences between rural and urban areas and the majority of them (16 studies) (Bellundagi et al., 2022; Bhargava et al., 2016; Biswas et al., 2017; Chakraborty et al., 2018; Ganie et al., 2017; Goonapienuwala et al., 2022; Harding et al., 2019; Hombaiah et al., 2021; Islam et al., 2020; Khan et al., 2022; Khatri et al., 2021; Mohan et al., 2019; Pandurangi et al., 2022; Prasad et al., 2016; Yadav et al., 2018; Young et al., 2020) found a higher prevalence of overweight and/or obesity in urban areas. Differences in overweight/obesity prevalence for males and females were inconsistent across studies; however, a pooled analysis combining 2416 population‐based measurements found that BMI was higher among girls compared to boys in South Asia (NCD Risk Factor Collaboration (NCD‐RisC), 2017). Other variations in overweight or obesity prevalence were reported across indicators for socioeconomic status, ethnicity, fast food and soft drink intake, maternal occupation and education, watching TV, less fruit and vegetable consumption, consumption of snack foods, physical inactivity, living distance from green space, parental weight, irregular food habits, skipping breakfast, and increased restaurant visits and street food shopping.

#### 3.4.1 Overweight and obesity intervention studies

Two studies implemented school‐based lifestyle intervention packages to address overweight and obesity in India (Figure 2 and Supporting Information: Table S6). One of these reported reductions in weight and energy intakes after 20 weeks, but no impact on BMI (Thakur et al., 2016). The other intervention also did not significantly impact BMI but was associated with positive effects on skinfold measurements and lifestyle practices (Nayak, 2016).

### 3.5 Micronutrient deficiencies

Nationally representative Demographic and Health Survey data on anaemia were available for girls aged 15–19 years in Bhutan, India, Maldives and Nepal, and girls aged 10–19 years in Pakistan (Table 2). The prevalence of anaemia ranged from 31.3% (Bhutan) to 60% (Maldives). India was the only other country where Demographic and Health Survey data were available for other micronutrient deficiencies. The prevalence of anaemia was reported by 58 studies across Afghanistan, Bangladesh, Bhutan, India, Nepal, Pakistan and Sri Lanka (Supporting Information: Table S5). Nationally representative data from Bangladesh, Sri Lanka and India indicated iron deficiency prevalence estimates of 7.1%, 19.2% and 30.0%, respectively (Kulkarni et al., 2021; Rahman et al., 2016; Wray et al., 2017). The prevalence of micronutrient deficiency reported by all studies (whether nationally representative or not) ranged from 1.8% (Pakistan, year undisclosed) (Zareen, 2021) to 87% (India in 2014–2015) (Ahankari et al., 2020). Fewer studies (*n* = 13) reported on the prevalence of iron deficiency and iron‐deficiency anaemia (IDA) but found ranges of 3.7% (Nepal in 2016; Ford et al., 2022) to 62.6% (Pakistan, year undisclosed) (Mohsin et al., 2016), and 1.3% (Bangladesh in 2011–12) (Rahman et al., 2016) to 41.5% (Pakistan in 2016–17) (Afridi et al., 2017), respectively.

Twenty‐eight studies analysed risk factors for anaemia, with 13 and six studies finding that anaemia was more prevalent among girls and among older adolescents respectively. Other variations in the prevalence of anaemia were reported across consumption of green leafy vegetables, milk, eggs, fruits and meat for more than 3 days a week, low dietary diversity, socioeconomic status, latrine use, iron folic acid (IFA) supplementation, level of education, infection with helminthiasis, washing hands before eating and use of footwear.

A total of nine studies from Bangladesh, India and Nepal reported on the prevalence of iodine deficiencies, which ranged from 6.3% (India, year undisclosed) (Bhattacharya & Chandra, 2019) to 31.8% (Nepal in 2015–2016) (Tamang, Khatiwada, et al., 2019). Clinical deficiency (goitre) was examined in three studies and ranged from 2.2% (India in 2016) (Bali et al., 2019) to 9.3% (India in 2017) (Shetty et al., 2019). Three out of 14 studies reported higher prevalence of iodine or goitre for girls compared to boys (Aslami et al., 2016; Gupta et al., 2020; Shetty et al., 2019). Five (Anusha et al., 2018; Aslami et al., 2016; Bali et al., 2018; Bhattacharyya et al., 2020; Shetty et al., 2019) studies reported on an association between the prevalence of iodine deficiencies and age, with four finding a higher prevalence in older adolescents (Aslami et al., 2016; Bali, et al., 2018; Bhattacharyya et al., 2020; Shetty et al., 2019). Three studies reported a positive association between adequately iodized salt and iodine status (Ansari & Khan, 2017; Anusha et al., 2018; Bhattacharya & Chandra, 2019). However, one study found a low prevalence of inadequate salt iodization in children with goitre, hypothesizing that there might be other contributing factors such as deficiency in other nutrients like iron, vitamin A or selenium (Shetty et al., 2019).

The prevalence of vitamin D deficiency was analysed in 17 studies from Afghanistan, Bangladesh, India, Sri Lanka and Nepal. The prevalence of vitamin D insufficiency and deficiency was 5.9% (India in 2014–2015) (Kapil et al., 2016) to 71% (India in 2018) (Mandlik, Kajale, et al., 2018) and 8.4% (India in 2011–2012) (Sarma et al., 2019) to 93% (India in 2014–2015) (Kapil et al., 2016), respectively. Four studies reported a higher prevalence of Vitamin D deficiency among girls compared to boys (Azizi & Tariq, 2019; Kapil et al., 2016, 2017; Siddiqee et al., 2022). Other factors associated with vitamin D status were sun exposure, calcium intake, diet, BMI, clothing, use of sun protector and darker skin (Azizi & Tariq, 2019; Sarma et al., 2019). A total of 14, nine and six studies analysed vitamin A, calcium and zinc intakes or deficiencies, with prevalence estimates ranging from 1.2% to 74% (deficiencies), 16.1% to 34% (intake) and 15.8% to 54% (deficiencies), for each micronutrient respectively.

#### 3.5.1 Micronutrient deficiency intervention studies

Eighteen intervention studies targeted some form of micronutrient deficiencies (Figure 2 and Supporting Information: Table S6). Thirteen intervention studies targeted anaemia; 10 studies through micronutrient fortification (Adams et al., 2017; Field et al., 2020; Gupta et al., 2022; M. V. Kumar & Erhardt, 2020; C. Kumar et al., 2021; Kunnath et al., 2022; Rahman et al., 2015; Ramírez‐Luzuriaga et al., 2018; Scott et al., 2018; Vinod Kumar & Erhardt, 2021), one through supplementation (Bansal et al., 2016), and two explored the efficacy of herbal remedies (Bhuvaneswari, 2020; Resmi et al., 2017). Of the 10 micronutrient fortification programmes, six had a positive impact on anaemia (Adams et al., 2017; Kumar & Erhardt, 2020; Kumar et al., 2021; Kunnath et al., 2022; Scott et al., 2018; Vinod Kumar & Erhardt, 2021). Supervised IFA supplementation with and without vitamin B_12_ was associated with increased mean haemoglobin levels and a reduction in the prevalence of anaemia (Bansal et al., 2016). The consumption of Indian gooseberry, jaggery and pumpkin leaf extract for 60 days was associated with a significant increase in haemoglobin levels, while honey dates and Indian gooseberry mix were associated with a reduction in the clinical symptoms of iron deficiency anaemia among adolescent girls (Bhuvaneswari, 2020; Resmi et al., 2017).

A total of two interventions targeted iodine consumption, with one making use of educational programmes and the other food fortification strategies. The educational intervention, conducted in government schools in Nepal between 2015 and 2017, reported slightly decreased excessive urinary iodine concentrations (Tamang, Gelal, et al., 2019). The food fortification intervention, conducted in India among school children, noted that the use of multiple micronutrient fortified salt in mid‐day meals for 1 year had no impact on urinary iodine concentrations (Kumar & Erhardt, 2020).

Four interventions (Adams et al., 2017; Kumar et al., 2021; Mandlik, Khadilkar, et al., 2018; Marwaha et al., 2019) analysed the impact of vitamin D supplementation or fortification programmes on school children, three in India (Kumar et al., 2021; Mandlik, Khadilkar, et al., 2018; Marwaha et al., 2019) and one in Bangladesh (Adams et al., 2017). Two supplementation studies from India conducted in 2014–2015 (Mandlik, Khadilkar, et al., 2018) and 2015–2017 (Marwaha et al., 2019), respectively, found a positive effect on serum 25(OH)D levels, while evidence on the impact of fortification from Bangladesh (2011–2017) and India (2017–2019) was mixed—Adams et al. (2017) found a positive impact of school‐based fortification programmes in Bangladesh, Kumar et al. (2021) found no impact of fortified milk supplementation.

Two studies reported a positive impact of fortification programmes on vitamin A deficiency (Adams et al., 2017; Rahman et al., 2015). One study, conducted in India, found that 200 mL fortified (vitamin A and D) milk supplementation for 1 year had a significant effect on children's calcium and B_12_ levels (Kumar et al., 2021). A study from India, conducted in six villages in Tamil Nadu among children aged 5–17 years and all women in the households, in 2012–2013, found that use of multiple micronutrient (iron, iodine, vitamin B_12_, folic acid and zinc) fortified salt for 8 months significantly decreased zinc deficiency from 32.7% to 12.4% (Vinod Kumar & Erhardt, 2021). However, the provision of zinc‐biofortified flour to adolescent girls 10–16 years for 25 weeks in Pakistan between 2019 and 2021 had no significant effect on plasma zinc concentrations (Gupta et al., 2022).

### 3.6 Dietary patterns and quality

Overall, 65 studies reported on dietary practices, with data reported for all countries in the South Asia region except for Bhutan (Supporting Information: Table S5).

Low dietary diversity among school‐age children and adolescents was reported in several studies, with diets primarily being cereal‐based (Nithya & Bhavani, 2018; Williams et al., 2019). Dietary diversity was found to increase from the least to the most affluent households in India in 2015–2016 (Rathi et al., 2017). However, those with a higher socioeconomic status were also less likely to demonstrate poor diet quality (Kumar et al., 2017). Expenditure on meat consumption increased with wealth in India in 2002, even though meat and dairy intakes were reported to be less than adequate in most studies (Humphries et al., 2017). Similarly, consumption of fruits and vegetables was mostly inadequate and did not vary much across wealth quintiles in India in 2002 (Humphries et al., 2017). Likewise, >75% of school‐age children in Bangladesh, the Maldives, Nepal, Pakistan and Sri Lanka consumed insufficient fruits and vegetables, with weekly consumption of fast‐food and daily consumption of soft drinks also common (survey years ranged from 2009 to 2016) (Fan & Zhang, 2020). Another study in India found energy‐dense snacks to be commonly consumed, with adolescents primarily eating fast food for the taste (Khongrangjem et al., 2018; Rathi et al., 2017). Hunger was also reported in four studies with 5.5%–43.4% adolescents skipping breakfast (Hassan et al., 2017; Jeyakumar & Ghugre, 2017; Saikia et al., 2016; Thapa et al., 2017).

#### 3.6.1 Dietary patterns and quality intervention studies

Only one intervention was identified that addressed diet quality (Figure 2 and Supporting Information: Table S6). This was an intervention study in Nepal (2015–2016) that utilized the Bhaskar Initiative for School Heart‐Health Empowerment Studies action tool to change dietary habits and found an aggregate decline in the consumption of junk food and unhealthy drinks following the implementation of the tool (Thapa et al., 2020).

## 4 DISCUSSION

This review summarized existing research on the nutritional status of school‐age children and adolescents aged 5–19 years in South Asia, as well as on interventions to reduce malnutrition in this age group. While the absolute number of studies describing the prevalence of all forms of malnutrition is high, our findings show that the data are limited in terms of their coverage. A substantial number of studies were identified (*n* = 296); however, more than half focused on India (54%). This could be due to the fact that almost three quarters of the population of the South Asia region reside in India (World Bank Group, 2022). Notable literature was also found from Pakistan (12%), Bangladesh (12%), Nepal (11%) and Sri Lanka (7%). Only 2% of studies were conducted in Afghanistan and only four papers examined the prevalence of malnutrition in Bhutan and the Maldives.

There was mixed evidence on the prevalence of overweight (0.2%–73%) and obesity (0%–38%) in South Asia, with the maximum and minimum ranges demonstrating notable heterogeneity across different parts of India. Nonetheless, when the prevalence of overweight and obesity was examined longitudinally using nationally representative Indian data, the prevalence of overweight increased among adolescents from 3% in 2005/2006 to 5.2% in 2015/2016 (Young et al., 2020). This may be due to the nutrition transition, with Indian studies reporting high levels of snacking and fast‐food consumption (Khongrangjem et al., 2018; Rathi et al., 2017). At the same time, a high prevalence of undernutrition persists, with South Asia demonstrating the highest global burden of undernutrition globally (Aguayo & Paintal, 2017). Longitudinal data sets suggested opportunities for catch‐up growth during childhood and adolescence (Ferdous et al., 2021; Gausman et al., 2019; Schott et al., 2019), with a nationally representative Indian study finding that thinness decreased among Indian adolescents over a 10‐year period (Young et al., 2020). However, transitions to energy‐dense and nutrient‐poor diets will undermine intergenerational gains in linear growth if left unchecked.

High prevalence of anaemia was evident across South Asian children and adolescents, with data indicating a comparatively higher prevalence of undernutrition and anaemia in adolescent girls than in adult women. This highlights the importance of adopting a lifecycle approach to nutrition policy and programming, rather than maintaining an explicit focus on pregnant and lactating women and children under 5 years of age (Rai et al., 2019; Tanwi et al., 2019). More research is also needed to understand the burden of iodine, vitamin D, calcium, vitamin A and zinc deficiencies across South Asia and on context‐specific risk factors and interventions to address these deficiencies.

Evidence from this review supports the importance of intervening to improve the nutritional status of school‐age children and adolescents in South Asia. There was a stark lack of intervention studies, particularly of sufficient durations to demonstrate impact—just three of 25 intervention studies included an intervention period of longer than 12 months. Overall, school feeding programmes, herbal remedies, vitamin D and IFA supplementation demonstrated benefits for undernutrition and micronutrient deficiencies. Of the identified intervention studies, few addressed overweight and obesity, or dietary quality, and most were administered through school settings. The mechanisms of existing large‐scale IFA supplementation and feeding programmes require strengthening to effectively target adolescents and to ensure adequate dietary quality and coverage. Such strengthening should include expansion to settings outside of just schools to ensure maximum coverage and to address a wider range of nutritional concerns. Many could also serve double duty for addressing underweight and overweight and obesity. While evidence suggests that fortification programmes could potentially address micronutrient deficiencies, levels of fortification and programme lengths were important determinants of efficacy. Similarly, the length of education programmes and their focus on addressing context‐specific drivers of malnutrition were important determinants of programme success. Although education programmes demonstrated positive effects on thinness, there were no school‐based education programmes identified to reduce BMI, though positive impacts of school education on dietary practices and skinfold thicknesses were documented.

### 4.1 Policy and research implications

This review found evidence of the triple burden of malnutrition among school‐age children and adolescents in South Asia, with micronutrient deficiencies being prevalent, alongside undernutrition and overweight/obesity. Yet, it is difficult to interpret trends within and between countries due to the use of different terminologies, reference data, cut‐off points and irregular data collection. The dearth of data in Bhutan, the Maldives and Afghanistan is particularly concerning. Greater standardization of anthropometric indicators and a commitment to more regular nutritional monitoring is required to support programmers and policymakers to monitor trends across the region, which can inform effective intervention design and commitment to national and global nutrition targets. Most intervention studies focused on addressing undernutrition and micronutrient deficiencies, despite rapidly increasing rates of overweight and obesity. Greater adoption of double duty actions would help tackle these coexisting forms of malnutrition, although additional research is needed to identify approaches that can simultaneously address all forms of malnutrition in South Asia.

### 4.2 Strengths and limitations

This scoping review involved a thorough literature search covering multiple databases and grey literature sources. Limitations included no analysis of quality (due to the breadth of the evidence retrieved); the potential for key relevant data to be missed by our search (especially those in languages not interpretable by the research team); and the lack of data reporting on malnutrition across all countries in South Asia.

## 5 CONCLUSION

This review shows that school‐age children and adolescents in South Asia are experiencing a triple burden of malnutrition. Key evidence gaps include a lack of data from some countries (particularly Bhutan, the Maldives and Afghanistan); a lack of data on micronutrient deficiencies; irregular undertaking of representative national nutrition surveys; and a lack of data from large‐scale intervention studies. The prevalence of malnutrition varies according to age, sex, rural–urban residence and wealth disparities, and these determinants should inform the development, targeting and implementation of nutrition policies and programmes. Several interventions, including education, fortification, supplementation and school feeding programmes, were identified as potential avenues for combating malnutrition. However, more research is needed across different contexts to understand the effectiveness of policies and programmes that can address the increasing burden of overnutrition.

## AUTHOR CONTRIBUTIONS

Vani Sethi, Zivai Murira, William Joe and Natasha Lelijveld formulated the research questions and study design. Tashi Choedon, Vani Sethi and Eilise Brennan were responsible for conducting the systematic literature review, summarizing the results and drafting the manuscript. Tashi Choedon, Vani Sethi, Oliver Huse, Christina Zorbas Kathryn Backholer, Natasha Lelijveld and Stephanie V. Wrottesley were responsible for finalizing the manuscript. All authors provided feedback on the draft manuscript.

## CONFLICTS OF INTEREST STATEMENT

Tashi Choedon and William Joe are employed by IEG India, which conducts work relating to malnutrition in the South Asia region. Vani Sethi and Zivai Murira are employed by UNICEF's South Asia Regional Office, which conducts work relating to malnutrition in the South Asia Region. Oliver Huse, Kathryn Backholer and Christina Zorbas receive consulting funds from UNICEF New York. Christina Zorbas receives additional funds from the Victorian Health Promotion Foundation (VicHealth) and Deakin University. The remaining authors declare no conflict of interest.

## Supporting information

Supporting information.

## Data Availability

The data that support the findings of this study are available in the supplementary material of this article
