# Supplementary material for: Nutritional status of school‐age children (5–19 years) in South Asia: A scoping review
Source: Matern Child Nutr. 2023 Dec 14;20(2):e13607. doi: 10.1111/mcn.13607 (PMC10981491; doi:10.1111/mcn.13607)
Supplement: Supplementary file 1 — Supporting information. [file MCN-20-e13607-s001.docx]

**Supplementary Table S1**: Search strategy for MEDLINE database

| MEDLINE Search Terms | | | |
| --- | --- | --- | --- |
| Population | Intervention | Comparison | Outcome |
| ((adolescen* or "school aged" or "school children" or "school child" or "school-age" or teen*) OR (exp adolescent/) OR (exp school child/))  AND  (Afghanistan OR Maldives OR Bangladesh OR Nepal OR Bhutan OR Pakistan OR India OR Sri Lanka OR "South Asia") | Any intervention that improves are defined outcomes | Not required | ((anaemia OR anemia OR iron OR ferrous OR ferric OR hepcidin OR ferritin OR transferrin OR heme OR haem) OR (exp anemia/) OR ( exp anemia, iron deficiency/) OR (exp anemia, megaloblastic/ ) OR (exp anemia, macrocytic/) OR (exp iron, dietary/) OR (exp receptor, transferrin /) OR (exp transferrin/ ) OR (exp hepcidin/) OR (exp ferritin/))  OR  (("vitamin A" OR retinol OR carotenoid* OR xerophthalmia) OR (exp vitamin A/) OR (exp carotenoid/)  OR  (("vitamin D" OR "vitamin D deficiency") OR (exp vitamin D/) OR (exp vitamin D deficiency/))  OR  ((zinc OR "zinc deficiency") OR (exp zinc/)  OR  ((calcium OR "calcium deficiency") OR (exp calcium/) OR (exp calcium, dietary/) OR (exp hypercalcemia/) OR (exp hypocalcaemia/))  OR  ((iodine OR "iodine deficiency" OR goitre OR goiter OR "iodised salt" OR "iodized salt") OR (exp goiter/) OR (exp iodine/))  OR  ((nutritio* OR malnutr* OR micronutrient OR undernutr* OR overnutr* OR undernourish* OR malnourish* OR stunt* OR "stunted growth" OR "growth failure" OR "linear growth" OR "failure to thrive" OR "growth disorder" OR "wasting" OR "wasted" OR overweight OR obes* or "high body mass index" OR "high BMI" OR thinness) OR (exp nutritional status/) OR (exp nutrition disorders) OR (exp malnutrition/) OR (severe acute malnutrition/) OR (exp thinness/) OR exp (exp overnutrition/) OR (exp growth disorder/) OR (exp failure to thrive/) OR (exp wasting syndrome/) OR (exp obesity/) OR (exp obesity management/) OR (exp diet-induced obesity/))  OR  ((diet OR "dietary pattern*" OR "healthy eating" OR "healthy diet" OR "dietary diversity" OR "food recall" OR "diet recall") OR (exp dietary /) OR (exp diet healthy) |

Note: *limited to human, English, since 2016-Nov 2022

**Supplementary Table S2**: Search strategy for Cochrane Library database

| Cochrane Library Search Terms | | | |
| --- | --- | --- | --- |
| Population | Intervention | Comparison | Outcome |
| (adolescen* OR "school aged" OR "school children" OR "school child" OR "school-age" OR teen*)  AND  (Afghanistan OR Maldives OR Bangladesh OR Nepal OR Bhutan OR Pakistan OR India OR Sri Lanka OR "South Asia") | Any intervention that improves are defined outcomes | Not required | (anaemia OR anemia OR iron OR ferrous OR ferric OR hepcidin OR ferritin OR transferrin OR heme OR haem OR "vitamin A" OR retinol OR carotenoid* OR xerophthalmia OR "vitamin D" OR "vitamins D deficiency" OR zinc OR "zinc deficiency" OR calcium OR "calcium deficiency" OR iodine OR "iodine deficiency" OR goitre OR goiter OR "iodised salt" OR "iodised salt"  OR  ((nutritio* OR malnutr* OR micronutrient OR undernutr* OR overnutr* OR undernourish* OR malnourish* OR stunt* OR "stunted growth" OR "growth failure" OR "linear growth" OR "failure to thrive" OR "growth disorder" OR "wasting" OR "wasted" OR overweight OR obes* or "high body mass index" OR "high BMI" OR thinness) OR (exp nutritional status/) OR (exp malnutrition/) OR (exp underweight/) OR exp (exp overnutrition/) OR (exp stunting/) OR (exp growth disorder/) OR (exp failure to thrive/) OR (exp wasting syndrome/) OR (exp obesity/) OR (exp obesity management/) OR (exp diet-induced obesity/))  OR  ((diet OR "dietary pattern*" OR "healthy eating" OR "healthy diet" OR "dietary diversity" OR "food recall" OR "diet recall") OR (exp dietary intake/) OR (exp dietary pattern/)) |

Note: *limited to human, English, since 2016-Nov 2022

**Supplementary Table S3**: Search strategy for EMBASE database

| EMBASE Search Terms | | | |
| --- | --- | --- | --- |
| Population | Intervention | Comparison | Outcome |
| (adolescen* OR "school aged" OR "school children" OR "school child" OR "school-age" OR teen*)  AND  (Afghanistan OR Maldives OR Bangladesh OR Nepal OR Bhutan OR Pakistan OR India OR Sri Lanka OR "South Asia") | Any intervention that improves are defined outcomes | Not required | ((anaemia OR anemia OR iron OR ferrous OR ferric OR hepcidin OR ferritin OR transferrin OR heme OR haem) OR (exp anemia/) OR ( exp iron deficiency anemia/) OR (exp megaloblastic anemia/ ) OR (exp microcytic anemia/) OR (exp macrocytic anemia/) OR (exp normochromic normocytic anemia/) OR (exp iron intake/) OR (exp iron depletion/) OR (exp iron absorption/) OR (exp iron deficiency/) OR (exp iron binding capacity/) OR (exp iron blood level/) OR (exp transferrin receptor/) OR (exp transferrin blood level/) OR (exp transferrin/ ) OR (exp hepcidin/) OR (exp ferritin/))  OR  (("vitamin A" OR retinol OR carotenoid* OR xerophthalmia) OR (exp retinol/) OR (exp carotenoid/) OR (exp retinol deficiency/))  OR  (("vitamin D" OR "vitamins D deficiency") OR (exp vitamin D/) OR (exp vitamin D deficiency/))  OR  ((zinc OR "zinc deficiency") OR (exp zinc/) OR (exp zinc deficiency/))  OR  ((calcium OR "calcium deficiency") OR (exp calcium/) OR (exp hypercalcemia/) OR (exp hypocalcaemia/))  OR  ((iodine OR "iodine deficiency" OR goitre OR goiter OR "iodised salt" OR "iodized salt") OR (exp iodine deficiency/) OR (exp goiter/) OR (exp iodine/))  OR  ((nutritio* OR malnutr* OR micronutrient OR undernutr* OR overnutr* OR undernourish* OR malnourish* OR stunt* OR "stunted growth" OR "growth failure" OR "linear growth" OR "failure to thrive" OR "growth disorder" OR "wasting" OR "wasted" OR overweight OR obes* or "high body mass index" OR "high BMI" OR thinness) OR (exp nutritional status/) OR (exp malnutrition/) OR (exp underweight/) OR exp (exp overnutrition/) OR (exp stunting/) OR (exp growth disorder/) OR (exp failure to thrive/) OR (exp wasting syndrome/) OR (exp obesity/) OR (exp obesity management/) OR (exp diet-induced obesity/))  OR  ((diet OR "dietary pattern*" OR "healthy eating" OR "healthy diet" OR "dietary diversity" OR "food recall" OR "diet recall") OR (exp dietary intake/) OR (exp dietary pattern/) |

Note: *limited to human, English, since 2016-Nov 2022

**Supplementary Table S4**: Summary of identified evidence on the nutritional status of school-aged children and adolescents in South Asia

| **Author (year)** | **Country (year of data collection)** | **Study design** | **Target population (sample size)** | **Outcome(s) of interest & classifications criteria used** | **Nutritional status or Interventions** | **Key findings** | **Author’s Notes** |
| --- | --- | --- | --- | --- | --- | --- | --- |
| ***Stunting*** | | | | | | | |
| 1. Gausman et al (2019) | India (2002-2016) | Longitudinal study | Children 8-15 years (n=1827) | Stunting (HAZ – WHO ref) | Nutritional Status | Stunting (28.6-27%) | Ethiopia, Peru, Vietnam - Young Lives Cohort  India had the largest percentage of children first stunted at age 5 who remained stunted at age 15 (28.7%)  However substantial percentage of children who are stunted recover – stunted at 5 and recovered by age 8 (40.5%) - 28.07%  of children stunted at 12 are unstunted by age 15, but still at increased risk of growth faltering – 18.8% recovered at age 8 falter again by age 15 |
| 1. Shinsugi et al (2020) | Sri Lanka (2017) | Cross-sectional study | School going children 5-10 years (n=538) | Stunting (HAZ) | Nutritional Status | Stunting (5.2%) | Urban areas |
| 1. Skroder et al (2018) | India | Prospective cohort study | Children approximately 9 years old (n=395) | Stunted (HAZ <-2 SD) | Nutritional Status | Stunting (22%) | Nested in MiniMat trial - Mother received micro-nutrients intervention during pregnancy |
| 1. Ferdous et al (2020) | Bangladesh | Large-scale cohort study | Childe approx. 9 years (n=517) | BAZ (WHO) | Nutritional Status | Stunted (28.6%)  21.9% of children who ever experience stunting dementated catch-up growth at 9 years | MiniMat – follow up study (6 months-9 years)  At 4.5 years follow up 28.6% of participants stunted  Growth trajectories from 6 months to 9 years: catch up (12.3%); unaltered (31.7%); faltering (12.1%) and normal (44%) |
| 1. Das SK et al (2021) | India (Young Lives Survey 2016-17) | Retrospective cohort analysis was undertaken from Young Lives panel survey | Adolescents aged 15y (n=1827) | WHO 2007 reference standard - moderately stunted (HAZ -3 to -2), and severely stunted (HAZ <-3). | Nutritional status | Overall stunting (27%), Severe stunting (5.3%), moderate stunting (21.7%) | Between Round-one and Round-five, cross-sectional prevalence of severe stunting decreased from 10.4% to 5.3%, while moderate stunting increased from 19.9% to 21.7%.  The stunting trajectory had shown gender differential where more faltering to severe stunting and lower recovery to the normal state was observed among girls between 8 and 12 years and among boys between 12 and 15 years. |
| ***Underweight and Thinness*** | | | | | | | |
| 1. Debnath et al (2016) | India | Cross-sectional survey | School going adolescent girls 9-14 years (n=387) | BMI: thinness grade 1 (17-18.5), grade II (BMI 16-17) and grade III (BMI< 16) – corresponds to CED | Nutritional status | Thinness (23.77%); Grade 1 (17.31%), Grade 2 (4.39%) and Grade 3 (2.07%) | No age specific trend |
| 1. Radhika et al (2018) | India (2011-12) | Cross-sectional study | Adolescents 10-19 years (n=3930) | 24 hr recall; DD (FAO – 14 food groups); BAZ (WHO growth ref standard) | Nutritional Status | 10-14 years: Thinness (35.1%); DDS =<5 (2.2%)  15-19 years: Thinness (18.9%); DDS =<5 (1.6%) | Representative sample for rural pop in India (NNMB dataset)  DD does not differ by age group/nutritional status  Younger adolescent girls (10–14 years) have a two fold higher risk of being undernourished  Family size, mother occupation and education, WASH, wealth ass with nutritional status |
| 1. Sethi et al (2019) | India (October 2016-April 2017) | Cross sectional survey | Adolescent girls 10-19 years (n=4628) | BAZ (WHO criteria) | Nutritional Status | Thinness (9.6-17.1%) and serve thinness (1.7-3.6%) | Tribal dominated areas |
| 1. Singh JK et al (2021) | India, UP (September 2016 and July 2017) | Cross-sectional survey | Adolescent girls aged 13 to 19 years (n=418) | WHO 2007 reference standard- Underweight, overweight and obesity | Nutritional status | underweight (49.8%), | Teenage adolescent girls who were SC/ST, primary or lower father’s education level,  and number of people in the family >4, from food insecure households was associated with being underweight.  Dietary behaviour-related factors such as vegetarian and <3 meals per day than their counterparts were associated with being underweight. |
| ***Overweight and obesity*** | | | | | | | |
| 1. Eshwar et al (2017) | India (January to April 2015) | Cross sectional survey | School going children 8-18 years (n=1496) | BMI - IAP – revised 2015 growth charts for height, weight, and BMI for 5–18 years old Indian children, WHO 2007 growth reference for 5–19 years, and OTF reference for 2–18 years. | Nutritional status | Total: Obesity: IAP (14%), WHO (11.1%), and IOTF standard (5.1%).  Overweight: IAP (19.1%), IOTF (15.8%), and WHO standards (15.3%) | Affluent school children  IOTF standards underestimate the obesity  IAP 2015 and WHO 2007 standards were very similar in classifying the obesity.  Higher prevalence of overweight ad obesity in boys |
| 1. Biswas et al (2016) | Bangladesh (1998-2015) | Systematic review | Children 13-19 years (n=117 174) | BMI cut off reference stands: WHO 2004, CDC 2000, WHO 2007, WHO 2006, WHO 2009, National obesity observatory 2012, WHO 1995, WHO 1993, Deitz and Robison, 1998 | Nutritional status | Overweight: 1998–2003 (3.6%); 2004–2009 (5.7%); 2010–2015 (7.9%)  Obesity:1998–2003 (9.7%); 2004–2009 (2.0%); 2010–2015 (9.0%) | Systematic review – 20 studies  Overweight gradually increasing, while obesity fluctuating  Factors: overweight parents, sedentary activities, higher income  Higher rates in urban compared to rural areas |
| 1. Viswanathan et al (2020) | India, Maharashtra (October 2017-October 2019) | Data collection- quasi-  experimental study | School going children 10-15 years (n=700) | IAP standard for BMI – for overweight and obesity, adult equivalent of 23 and 27 BMI cut-off lines were utilised | Nutritional status | Overweight (12.9%) and obesity (3.4%)  Females high prevalence of overweight, while males higher prevalence of obesity | No age increase/decrease in prevalence  Obesity risk factors: Frequent consumption of fast-food (OR 2.31), carbonated drinks (OR 0.39)  watching television for more than 1 (OR 3.9)  Rural area |
| 1. Viswanathan et al (2020) | India, Maharashtra (March-October 2018) | Cross-sectional survey | School going adolescent 12-15 years (n=300) | IAP standard for BMI – for overweight and obesity, adult equivalent of 23 and 27 BMI cut-off lines were utilised | Nutritional status | Obesity (5%) and overweight (9%) | Obesity prevalence higher in males  Consumption of fast food (OR 5.9), snacks (OR 3.8), soft-drinks (OR 4,5) and watching television (OR 5.8) were significant risk factors.  Under detection of overweight-obesity and over detection of undernutrition that was observed with the WHO reference and the IAP Reference compared to regional standards – IAP + Who standards similar detection of obesity/overweight |
| 1. Wickramasinghe et al (2017) | Sri Lanka (September 2004-April 2005) | Cross sectional survey | School going adolescent 5-15 years (n=309) | IOTF reference standards: overweight (BMI 25 kg/m^2^); obesity (BMI 30 kg/m^2^); thinness (BMI <18·5 kg/m^2^). | Nutritional status | Obese (4.2%) and overweight (11.3%) | Many anthropometric parameters showed significant relationship with insulin resistance |
| 1. Khadgawat et al (2016) | India (2013) | Cross sectional survey | School going girls 6-17 years (n=335) | Obesity defined IOTF criteria: BMI: overweight (25 ≤ BMI < 30 kg/m^2^) and obese (BMI ≥ 30 kg/m^2^) | Nutritional status | Overweight/obese (19.1%) | Private schools |
| 1. Ganie et al (2017) | India (September 2011-June 2013) | Cross sectional survey | School going children 6-12 years (n=2024) | BAZ (CDC); overweight (85^th^-95^th^ percentile); obesity (=>95^th^ percentile) | Nutritional status | Overweight (6.69%) and obesity (4.64%) | Majority from fee paying private schools  Higher prevalence of obesity in girls and urban areas, younger age groups |
| 1. Goel et al (2016) | India | Prospective cross-sectional observational study | School going adolescents 14-17 years (n=1221) | BMI (CDC): thinness (<5^th^ percentile); overweight (85^th^-95^th^ percentile); obesity (=>95^th^ percentile) | Nutritional status | Obesity (5.65%) and overweight (9.18%) | Urban schools  27 students that were thin were excluded from study (2.21%)  No diff between genders |
| 1. Jayawardena et al (2017) | South Asia: Bangladesh (2009); India (2008-11); Nepal (2011); Pakistan (2010) Sri Lanka (2009-10) | Systematic Review | Adolescents 11-19 years (Bangladesh n=5560, India n=798, Nepal n=241, Pakistan n=431, Sri Lanka n=465) | WHO (BAZ); percentiles IAP Growth Chart; WHO BM (overweight <25, obese (<30); IOTF | Nutritional status | Bangladesh: overweight (12.2%) and Obesity (3%)  India: overweight (19%) and obesity (7.1%) Nepal: overweight (12.1%) and obesity (4.9%)  Pakistan: overweight (17%) and obesity (11%)  Sri Lanka: overweight (11%) and obesity (2.4%)  Eating meals outside of the home, while watching TV, frequently visiting restaurants and buying lunch from school canteen, missing meals leads to overweight/obesity  Protective effect of fruit/veg consumption | 8 cross sectional prevalence studies and 28 studies evaluating nutrition factors  At present there is no consensus regarding ethnic-specific cut-offs for teenagers- international BMI/adult cut off may underestimate |
| 1. Khan et al (2019) | Bangladesh (2014) | Global School Health Survey- Population-based cross-sectional survey | School going adolescents 11-17 years (n=2989) | BAZ (WHO): overweight (BAZ <1SD); obesity (BAZ <2SD) | Nutritional status | Overweight/obese (7.4-9.9%)  Adolescent high frequency of soft drink consumption had obesity rates 12.4-18.75 | Global School-based Student Health Survey  Consumption of fruit and vegetables, soft drinks and fast food, physical activity were found to be significantly related to being overweight or obese  Prevalence higher in males  Male adolescents with high food insecurity are at an increased risk of being overweight or obese – poor diet quality  Fruit and vegetables can help restrain excessive weight gain among children |
| 1. Kumar et al (2017) | India (August 2014-December 2015) | Cross-sectional survey | School going children 5-15 years (n=138) | BAZ (WHO): overweight (>=1SD); obesity (>+2SD) | Nutritional status | Overweight (29%) and obesity (38%) | Rural residential school – boarding school |
| 1. Macwana et al (2016) | India (August 2012-December 2013) | Cross-sectional survey | School going adolescent 11-19 years (n=1050) | BAZ (WHO): overweight (>=1SD); obesity (>+2SD) | Nutritional status | Overweight (7.8%) and obesity (2.7%) | Stat sig higher risk in early adolescent group, for boys (3 times), student that reported outside food consumption, or had a habit of skipping meals, sedentary activities (TV) |
| 1. Mohan et al (2019) | India  (March 2015-June 2017) | Cross- sectional survey | School going children 11-17 years (n=1959) | IAP growth reference: thinness (<3d adult equivalent line; overweight >23^rd^ adult equivalent line; obese >27^th^ adult equivalent line)  BMI (WHO growth reference) | Nutritional status | Overweight (8.6-10.5%) and obese (4.1-8.6%) | Rural and urban school  High proportion overweight/obese from urban schools |
| 1. Pawar et al (2016) | India (December 2014-January 2015) | Cross- sectional survey | School going children 11-16 years (n=1828) | Cole et al (International): Overweight (BMI >25kg/m2) and obesity (BMI >30kg/m2)  And  Khadikar et al (India): Overweight (23kg/m2) and obesity (28kg/m2) | Nutritional status | Overweight (11.3-17.5%) obesity (3.3-7.8%) | More prevalent in private schools  Large proportion of student would be missed if solely using international criteria  No difference between boys and girls |
| 1. Pawar et al (2016) | India | Cross- sectional survey | School going children 11-15 years (n=616) | Overweight (BMI >25kg/m2) and obesity (BMI >30kg/m2)  And Overweight (23kg/m2) and obesity (28kg/m2) | Nutritional status | Overweight (73% & 49%); obesity (27% & 12%) | Sample frame: one school  Males had higher prevalence of abdominal obesity  Large proportion of student would be missed if solely using international criteria |
| 1. Piryani et al (2016) | Nepal (October-November 2014) | Cross- sectional survey | School going children 16-19 years (n=360) | BMI (WHO): overweight (<+1SD) | Nutritional status | Overweight (12.2%) | Associated factors: males, private school higher socio-economic status, watching TV for more than 2hrs, consuming fruit or veg 4 times or less a week.  Urban school |
| 1. Prasad et al (2016) | India | Cross- sectional survey | School going children 10-18 years (n=2465) | BAZ– IAP cut offs | Nutritional status | Overweight (9.7%) and obesity (4.3%) | No difference between sexes, highest among older age groups (16-18 years)  Higher among urban and private school students |
| 1. Ranjani et al (2016) | India (1990-2013) | Systematic review | Adolescent 2-18 years (n=435 162) | Majority used IOTF, WHO and CDC cut offs; Other include Gomez classification, EHPA and that of Must *et al,* Rosner *et al*^,^ Pandey *et al* and Khadilkar *et al* | Nutritional status | Obesity and overweight/obesity:  2001-05: obesity (5.5%) and overweight/obesity (16.3%)  2006-10: obesity (4%) and overweight/obesity (17.4%)  After 2010: obesity (4.65) and overweight and obesity (19.3%)  Overweight prior 2001 (13.9%) and after 2010 (13.9%) | A systematic review  52 studies: prevalence studies for overweight (43 studies) and obesity (50 studies) – covered 16 states  Obesity somewhat constant but increasing prevalence of overweight and combined overweight/obesity |
| 1. Roman-Vinas et al (2016) | India (September 2011-Decembver 2013) | Cross- sectional survey | Children 9-11 years (n=532) | BAZ (WHO); obesity (BAZ>=2SD) | Nutritional status | Obesity (10.7%) | Multi-country study (ISCOLE sites)  At least 60 min per day of MVPA, no more than 2 h per day spent on recreational screen time, and a sleep duration between 9 and 11 h per night was associated with lower odds ratios for obesity  62% meet screen time recommendation; 25% meet MVPA and 26.9% meet sleep recommendation |
| 1. Saikia et al (2018) | India (May 2015-April 2016) | Cross- sectional survey | School going adolescent 10-14 years (n=1096) | WHO 2007 growth reference | Nutritional status | Overweight (20.9%) and obesity (10.2%) | Private and public schools |
| 1. Shahid et al (2017) | Pakistan (January – February 2017) | Cross- sectional survey | School going adolescent 10-16 years (n=197) | Overweight/obesity: BMI <25kg/m2 | Nutritional status | Overweight/obesity (18.2%) |  |
| 1. Shakya et al (2020) | Nepal | Cross- sectional survey | School going adolescents 12-17 years (n=356) | CDC reference standard | Nutritional status | Overweight (13.8%) and obesity (1.4%) | Private schools |
| 1. Chakraborty et al (2020) | India (January 2012-November 2014) | Cross-sectional survey | School going children 10-17 years (n=4438) | Cole et al. (IOTF cut off) | Nutritional Status | Overweight (16.7%) and obesity (6.56%) | Overweight/obesity more prevalent in private school participants |
| 1. Arora et al (2019) | India | Cross sectional survey | School going children 12-17 years (n=456) | BMI: overweight (<25kg/m2) and obesity (<=30 kg/m2) | Nutritional Status | Overweight (16.23%) and obese (5.48%) | Urban area only |
| 1. Ashraf et al (2017) | Pakistan (April-October 2010) | Cross sectional survey | Children 5-14 years (n=305) | BAZ (WHO) | Nutritional Status | Overweight (7.5%) and obese (8.5%) | Hospital based – healthy children  58% correctly defied child weights – parents of obese children most likely to misclassify child weight – 62% of obese children were perceived as thinness |
| 1. Asif et al (2018) | Pakistan (March-June 2016) | Cross sectional survey | Adolescents 12-18 years (n=4962) | BMI (CDC) | Nutritional Status | Overweight (10%) and obesity (5%) | Urban areas – three major cities  Girls had higher burden of overweigh and obesity |
| 1. Asif et al (2018) | Pakistan (March-June 2016) | Cross sectional survey | Children 5-14 years (n=7921) | BAZ (WHO) | Nutritional Status | Overweight (16%) and obesity (3.3%) | Higher obesity prevalence in boys, similar prevalence for overweight  Population based – three major cities |
| 1. Chaput et al (2018) | India (September 2011 – December 2013) | Cross-sectional study | Adolescents 9-11 years (n=553) | BAZ (WHO) | Nutritional Status | Obesity (10.9%); 8.6 sedentary hours/day | ISCOLE  Obesity is a multifactorial condition, and both MVPA and sedentary behaviour, alone or in combination, are not able to accurately detect children with obesity |
| 1. Jeevandam et al (2018) | India | Cross sectional survey | Adolescents 18-19 years (n=168) | BMI - thinness (<18.5 kg/m2); overweight (>23 kg/m2); obesity (>25 kg/m2) | Nutritional Status | Overweight (25%) and obesity (28.6%) | University students  Females higher prevalence of overweight and obesity |
| 1. Nasreddine et al (2018) | Pakistan & Afghanistan (2013 – 2018) | Review | School aged children | Overweight and obesity (BAZ <1SD) | Nutritional Status | Overweight and obesity Pakistani (1%) and Afghanistan (2.7% - girls only) | Systematic review includes School Based Student Health Surveys |
| 1. Kunwar et al (2018) | India Kunwar (2018) | Cross sectional survey | School going children 6-18 years (n=886) | BAZ (WHO criteria) | Nutritional Status | Overweight (9.76-10.97%); obese (1.22-3.23%) | Population: Large military station |
| 1. Dendup et al (2021) | Bhutan (Global School based Student Health Survey, October- November 2016) | Global School-based Student Health Survey data- Cross-sectional design | School going adolescents aged 13-18 years (n= 7341) | WHO 2007 reference standard BAZ- overweight/obesity | Nutritional status | Overweight /obesity (11.4%)  The prevalence of overweight was around two times higher among girls than boys (14.72% vs. 7.74%) | Females, adolescents who  were day students, and sedentary had higher odds of being overweight.  Those aged >15 years and received education on  healthy eating had reduced odds |
| 1. Khatri et al (2021) | Nepal | Cross-sectional study | Secondary school students aged 11-19 years (n=279) | WHO 2007 reference standard for overweight/obesity | Nutritional status | Overweight/obesity (9.31%) | The early aged adolescents (11-13y), living in urban areas, sedentary behaviour were more likely overweight than  their counterparts. |
| 1. Batish et al (2021) | India (January 2019 to December 2019) | Cross-sectional study | School going children aged 6-16 years (n=355) | WHO 2007 reference standard for overweight and obesity | Nutritional status | Overweight (14.8%), obese (7.8%) |  |
| 1. Chiplonkar et al (2017) | India | Secondary analysis of previous multicenter cross-sectional studies | School going children 5-17 years (n=3832) | Adult equivalent BMI Z scores using values for Asians: overweight (BMI 23 to 28 kg/m2) and obese (BMI >28 kg/m2) | Nutritional Status | Overweight (11.1-23.3%) and obesity (2-11.5%) | Private schools – from five major cities in 5 different states |
| ***Micronutrient deficiencies*** | | | | | | | |
| 1. Sabharwal et al (2018) | India (October 2016 to September 2017) | Cross sectional survey | Adolescent 6-15 years (n=105) | Serum 25-(OH)-D: severe vitamin D deficiency (< 5 ng/ml); moderate hypovitaminosis (5-10ng/ml); mild hypovitaminosis (20 ng/ml). | Nutritional status | 6-10 years: Severe Vit D deficiency (60%), moderate (31.82%), mild (32.71%)  11-15 years: Severe Vit D deficiency (30%), moderate (27.27%), mild (40.19%)  Overall: Females had decreased vitamin D levels as compared to males. | Wide variations in serum 25-(OH)-D across the various seasons and region of different geography/latitude  Insufficient daily intake of vitamin D on daily basis can lead to its deficiency in the summer and autumn despite sufficient exposure to sunlight.  Despite its sunny environment, hypovitaminosis is common in India. |
| 1. Afridi et al (2017) | Pakistan (2016-2017) | Cross sectional survey | Adolescent 7-11 years (n=498) | Anaemia (hemoglobulin <11 g/dL); IDA (anaemia alongside decrease in mean corpuscular volume, hypochromic red blood cells, low serum iron and high total iron binding capacity | Nutritional status | Children 7-9years: IDA (33.3%)  Children 9-11 years: IDA (41.45%) | Children presenting with pallor at Akhtar Saeed Trust Teaching Hospital Lahore Pakistan were included –does not represent general population prevalence.  Females more effected – cultural reasons. |
| 1. William et al. (2016) | India | Cross sectional survey | School going adolescent (15-18 years) | Anaemia and haemoglobin was estimated by calorimetric method;  dietary habits via interview | Nutritional status | 61.3% of adolescent were anaemic  Adolescents who consumed vegetables, green leafy vegetables, milk, egg, fruits and meat for more than 3 days a week were at lesser risk of developing anaemia. | Anaemia associated with number of siblings, family income, latrine use. |
| 1. Rakesh et al (2019) | India | Cross sectional survey | School going adolescent (n=880) | Anaemia: children less than 12 years (: <11.5 g/dl for); girls 12 to 18 years and boys less than 14 years (<12 g/dl); boys from 15 to 18 years of age (<13 g/dl for). Severe anaemia (<8 g/dl); moderate anaemia (8.1 and 10 g/dl) | Nutritional status | Anaemia (44%); Among them 0.8% had severe anaemia, 3.5% had moderate anaemia and 39.7% had mild anaemia  81.7%, 21% and 52.6% reported not in the habit of consuming meat, green leafy vegetables and citrus fruits respectively, at least three times on a usual week | Private and public school included  Anaemia was highest in the age group of 12–15  Anaemia associated with age, gender and regular intake of tea/coffee along with major meals.  Above WHO prevalence for supplementation (30%).  Different cut off used in DLHS surveys than WHO cut offs |
| 1. Ahankari et al (2017 & 2019) | India (April 2014-June 2015) | Cross sectional survey | Adolescent girls 13-17 years (n=1010) | 7 day food frequency recall  Anaemia (Hb level <12.0 g/dl); severe (Hb 7.9 g/dl) moderate (Hb 8.0e10.9 g/dl) and mild anaemia (Hb 11.0e11.9 g/dl)  thinness (BMI <18.5kg/m²) | Nutritional status | Anaemia (87%); severe anaemia (5%), moderate anaemia (65%) and mild anaemia (17%) of the study participants  thinness (67%)  Ate pure milk (21.5%), green leafy veg (28.4%), bean sprouts (4.2%), pulses-lentils (69.6%), fruits (23.3%), rice (89.8%), eggs (2.9%), chicken (0.89%) and goat meat (0.05%) => 3 times a week. | No participants reported systemic diseases that could influence Hb levels  Factors associated with anaemia in adjusted models - age, MUAC, current, IFA supplementation, fruit/fruit juice, rice intake, and current education  Rice- high consumption brown rice, higher iron content than polished white rice. |
| 1. Allen et al (2017) | Sri Lanka (June 2009 and July 2010) | Cross sectional survey | School going children 11-19 years (n=5912) | Anaemia: Children <12 years (Hb <11.5.0 g/dl); females =>12 years and males 12-14 years (Hb <12.0 g/dl); males 15+ years (Hb <13.0 g/dl)  Iron depletion (ferritin <15ng/ml); cellular iron deficiency (ferritin <15ng/ml & transferrin receptor >28.1nmol/l); iron deficiency anaemia (cellular iron deficiency with low haemoglobin according to gender) | Nutritional status | Anaemia (8.1%); severely anaemic males (0.031%) and females (0.54%)  Iron depletion (19.3%) and cellar iron deficiency (11.6%) and iron deficiency anaemia (3.9%) | Across 25 districts and haemoglobin levels adjusted for differing altitudes  Anaemia more common in females  All stages of iron deficiency were more common in younger students; this may be due to the increased demand for iron during the most active phase of the pubertal growth spurt that occurs before age 16 years in most children.  Folate and/or vitamin B_12_ may also account for higher frequency of anaemia compared to IDA  A part of national survey |
| 1. Ansari et al (2017) | India (January-December 2013) | Cross sectional survey | School going children (6-12 years) | Iodine deficiency: urinary iodine concentrations <100 ug/L; mild (50-99 ug/L, moderate (20-49 ug/L and serve (<20ug/L) iodine deficiency | Nutritional status | Iodine deficiency (23.3%); Severe (10%), moderate (5.5%) and mild (7.8%) iodine deficiency | If all salt is iodized adequately and all families use only iodized salt, then iodine deficiency will no longer threaten the health and development of children |
| 1. Anusha et al (2018) | India (July 2015-Febuary 2016) | Cross sectional survey | School going children 6-14years (n=670) | Goitre: Grade 1 (Persons with neck thickening as a result of enlarged thyroid, palpable goitre, not visible in normal position of neck. Includes nodular goitre also) and Garde 2 (Neck swelling, visible when the neck is in normal position, corresponding to enlarged thyroid found in palpation)  Dietary questionnaire  Salt iodisation levels (Rapid Test Kits) | Nutritional status | Diet: consume non vegetarian food (93.8%)  Goitre (6.6%): Grade 1 (4.8%) and Grade 2 (1.8%)  Percentage of household achieving adequality iodised salt (44.5%) | Prevalence of goitre higher among younger children  Significant association between adequality iodised salt and goitre |
| 1. Aslami et al (2016) | India (2012) | Cross sectional survey | School going children age 6-12 years (n=790) | Grade 1 (Persons with neck thickening as a result of enlarged thyroid, palpable goitre, not visible in normal position of neck. Includes nodular goitre also) and Grade 2 (Neck swelling, visible when the neck is in normal position, corresponding to enlarged thyroid found in palpation  Biochemical iodine deficiency (n=132)(<100ug/l)  Salt iodisation status (n=121): Inadequately iodised (<15ppm) | Nutritional status | Goitre: Grade one (5.2%) and Grade 2 (0%).  Biochemical iodine deficiency (22.5%)  Households consuming adequately iodized salt (50.4%) | Higher goitre prevalence in girls and older children |
| 1. Bali et al (2018) | India (May-June 2016) | Cross sectional survey | School going children 6-12 years (n=2700) | Grade 1 (Persons with neck thickening as a result of enlarged thyroid, palpable goitre, not visible in normal position of neck. Includes nodular goitre also) and Grade 2 (Neck swelling, visible when the neck is in normal position, corresponding to enlarged thyroid found in palpation  Urine iodine excretion (n=70)(WHO criteria)  Salt iodisation status (n=432) Inadequately iodised (<15ppm) | Nutritional status | Goitre: grade 1 (2.08%) and Grade II (0.41%)  Inadequately iodised salt (27.6%)  Insufficient urine iodine excretion (25.9%): Mild (17%), moderate (8%) and serve (0.7%) iodine deficiency  Proportion of children with more than adequate iodine intake (10%) and excessive (36%) | Insufficient UIE more prevalent in older children |
| 1. Bali et al (2019) | India (April-May 2016) | Descriptive cross-sectional study | School going children 6-12 years (n=2700) | Grade 1 (Persons with neck thickening as a result of enlarged thyroid, palpable goitre, not visible in normal position of neck. Includes nodular goitre also) and Grade 2 (Neck swelling, visible when the neck is in normal position, corresponding to enlarged thyroid found in palpation  Urine iodine excretion (n=270): Severe (<20 ug/l), moderate (20-49 ug/l) and mild (50-99 ug/l) iodine deficiency; above requirement (200-299 ug/L) and excessive UIE (=>300 ug/L)  Salt iodisation status (n=90): Inadequately iodised (<15ppm) | Nutritional status | Goitre: Grade 1 (2.2%) and Grade 2 (0)  Insufficient UIE (24.7%): severe (8.6%), moderate (6.4%) and mild (9.7%).  Above requirements (18.7%) and excessive (37.5%)  Adequately iodised salt (90.6%) | Goitre higher in males |
| 1. Bawaskar et al (2020) | India | Cross sectional survey | School going children 5-15 years (n=200) | Vitamin D deficiency: <10 ng/ml, 11-15 ng/ml and 16-30ng/ml | Nutritional status | Vitamin D deficiency: <10 ng/ml (9.5%), 11-15 ng/ml (20%) and 16-30ng/ml (47%) | High for all socio-economic groups.  High socio-economic status: 66% >30ng/ml, but also situated on hill top |
| 1. Bhattaacharya et al (2019) | India | Cross sectional survey | School going children 6-12years (n=3500) | Grade 1 (Persons with neck thickening as a result of enlarged thyroid, palpable goitre, not visible in normal position of neck. Includes nodular goitre also) and Grade 2 (Neck swelling, visible when the neck is in normal position, corresponding to enlarged thyroid found in palpation  UIE (n=240): Insufficient (<9.9 ug/dl); above requirements (20-29.9 ug/dl); excessive (=>30ug/dl)  Salt iodisation status (n=210): Inadequately iodised (<15ppm) | Nutritional status | Goitre (5.5%): Grade 1 (5.4%) and Grade 2 (0.6%)  UIE: iodine deficient (6.25%); more than adequate (39.17%); excessive (22.08%).  11.9% of salt sample inadequately idolised. | Mild public health problem in Kolkata  Significant correlation between UIE and iodine content of salt samples  Overall: Iodine intake adequate to above requirements in all areas; iodine nutritional status was optimal to more than adequate  Kolkata is clinically mild iodine deficient.  Regular consumption of goitrous plant food |
| 1. Bhattaacharya et al (2020) | India, East Khasi Hill districts (June 2016-December 2018) | Cross sectional survey | School going children 6-10 years (n=2700) | Grade 1 (Persons with neck thickening as a result of enlarged thyroid, palpable goitre, not visible in normal position of neck. Includes nodular goitre also) and Grade 2 (Neck swelling, visible when the neck is in normal position, corresponding to enlarged thyroid found in palpation  UIE: Iodine deficient (<99 ug/L); severe iodine deficiency (<20ug/L)  Salt iodisation status (n=540): Inadequately iodised (<15ppm)  WAZ: Moderate (=<-2SD) and server (=<-3SD) underweight  HAZ: Moderate (=<-2SD) and server (=<-3SD) stunting | Nutritional status | Goitre (7.22%): Grade 1 (6%) and Grade 2 (0.7%)  Moderately (3.9%) and severely (25.1%) Underweight: moderate (31%) and severe (16.8%) stunting  95.9% of salt sample were adequately iodised | Higher prevalence of goitre in older age groups  Higher than 5% cut off – IDD public health problem  A higher goitre prevalence was seen among thin and stunted children  Significant association between goitre and UIE |
| 1. Campbell et al (2018) | Bhutan (2015) | Secondary research | Adolescents 10-19 years (n=11 216) | Anaemia: mild (Hb < 12 g/dl) or for girls 10 or 11 years old (Hb < 11.5 g/dl); moderate (Hb < 11 g/dl); severe (Hb < 8 g/dl) | Nutritional status | Anaemia (29.3%); Mild (15.1%), moderate (12.9%) and severe (1.3%)  Surprisingly high anaemia prevalence given the relatively good nutritional status | National Nutrition Survey  Cut off based WHO guidelines  Risk factors: older age, living in west/central regions, living at home rather than school – socio-economic/diet quality not associated  *by age if needed |
| 1. Chalise et al (2018) | Nepal (2014) | Cross sectional survey | Adolescents 10-19 years (n=3655) | BMI-z scores: thinness (<-2 SD); overweight (<+1SD).  Anaemia adjusted for altitudes: children 12-14 years and females 15+ years (mild (110-119 g/l), moderate (80-109 g/l), severe (<80)); men 15+ (mild (110-129 g/l), moderate (80-109 g/l), severe (<80g/l) | Nutritional status | Anaemia (31%):  Stag significant associated with age (highest among 15-19 year olds (37%), gender, DD and living in Teria areas | National Adolescent Nutrition Survey  Teria areas – higher poverty, lack of sanitation facilities |
| 1. Wray et al (2016) | Sri Lanka (June 2009 and July 2010) | Cross sectional survey | School going children 12-19 years (n=2273) | Anaemia (Hb < 12 g/dL in girls and in boys 12–14 years, Hb < 13 g/dL in boys 14–19 years); Iron deficiency (ferritin < 15 ng/mL, or ferritin < 30 ng/mL if CRP > 5 mg/L, and sTfR‐F > 2) | Nutritional status | Anaemia (19.8%); iron deficiency (19.2%) – girls (27.6%) and boys (10.4%).  Study was enriched with patients likely to carry hemoglobinopathies, thus population prevalence of anaemia and iron deficiency in Sri Lanka is even lower still. | National representative  Prevalence of anaemia was below the threshold for which a routine universal iron intervention programme would be considered |
| 1. Ahankari et al (2016) | India | Cross sectional survey | Adolescent 13-17 years (n=766) | Anaemia (Hb <12g/dl); severe anaemia (Hb =<7.9 g/dl) | Nutritional status | Anaemia (84%); severe anaemia (5%) | Sahli’s haemoeter  Rural India |
| 1. Ahankari et al (2020) | India (2018) | Cross sectional survey | Adolescent girls 10-19 years (n=401) | Anaemia (Hb <12g/dl) | Nutritional status | Anaemia (31%) | Rural India  Associated with anthropometric measurements |
| 1. Didzun et al (2019) | India (January 2015-December 2016) | Cross sectional survey | Adolescent 15-19 years (n=132 214) | Men: anaemia (<13g/dl), moderate (<11 g/dl), severe (<8 g/dl)  Females: Anaemia (<12 g/dl), moderate (<11 g/dl), severe (<8 g/dl) | Nutritional status | Anaemia: Men (29.3%) and women (53.9%)  Moderate anaemia: men (5.7%) and women (27.9%)  Severe anaemia: Men (0.5%) and women (2.2%) | Nationally Family Household Survey  Varied between states  Anaemia among men is a substantial public health issue in India  In 2013 National Iron Plus Initiative extended the provision of IFA to boys aged 10–19 years.  The few existing evaluations of the National Nutritional Anaemia Prevention Programme suggest that it has not been successful in reducing anaemia prevalence in any age group.  [Anemia Mukt Bharat](https://anemiamuktbharat.info/dashboard/#/) launched 2018 - large-scale initiative to tackle anaemia, includes boys 10-19 years |
| 1. Habib et al (2019) | Pakistan (2015) | Cross sectional survey | Adolescent girls 10-15 years (n=626) | Anaemia: 10–11 years, severw (Hb ≤8.0 g/dL), moderate (8.1 to 10.9 g/dL), and mild (11 to 11.4 g/dL); ≥12 years, severe (Hb less ≤8.0 g/dL), moderate (8.1 to 10.9 g/dL), and mild( 11 to 1.9 g/dL) | Nutritional status | Anaemia (47.9%): mild (20.4%), moderate (24.8%) and severe (2.7%) | Anaemia is a severe health problem  Associated factors: education, duration of menstruation, heavy blood loss during menstruation, communicable diseases, healthcare utilization, meals regularity, and exercise habits, income etc |
| 1. Karageorgou et al (2018) | Bangladesh (2011-12) | Bangladesh Household Integrated Survey (BIHS)- Cross sectional survey | Adolescents 6-19 years (n=6963) | Household survey & 24hr dietary recall | Nutritional status | 6-10 years: Energy (1596.7 kcal/d), Vit A (163 UG RAE/d), Vit D (0.8 ug/d), calcium (213.9 mg/d), zinc (6.6 mg/d)  11-19 years: Energy (2186.1) kcal/d), Vit A (222.4 UG RAE/d), Vit D (1.2 ug/d), calcium (282.6 mg/d), zinc (9.1 mg/d) | 2011–2012 Bangladesh Household Integrated Survey (BIHS) |
| 1. Leyvraz et al (2016) | Bangladesh (October-December 2011) | National Micronutrients Status Survey- Cross-sectional nationally  representative survey | Children 6-14 years | Food frequency questionnaire: Iron, Zinc and Vit A intakes | Nutritional status | Dietary intake for iron zinc and Vit A covers 28%, 50.5% and 23.7% of needs | National Micronutrients Status Survey December 2011  Zinc (3.8 mg/d) Vitamin A (277.9 RE/d)  Zinc/Vita correlated with socio-economic status  Based on the consumption patterns, rice is the most suitable vehicle for fortification |
| 1. Din et al (2019) | Pakistan (September 2014-April 2015) | Cross sectional survey | School aged children 5-12 years (n=318) | Anaemia (Hb<11.5gm/dl): Mild (Hb 10-11 g/dl), moderate (Hb 7-10 g.dl), severe (Hb <7 g/dL) | Nutritional status | Anaemia (37.1%): mild (30.5%), moderate (5.35%) and severe (1.26%) | Prevalence increased by age |
| 1. Gupta et al (2020) | India (2010-14) | Cross- sectional survey | School going adolescents 6-14 years (n=2700) | Goitre (WHO guidelines); urinary iodine concentrations (n=270) >100ug/L; | Nutritional status | Goitre (6.4%); Grade 1 (4.2%) & Grade 2 (2.2%).  UIC (18.5%) | Goitre more prevalent in females  Iodization indicators reflected adequate iodization in the population. |
| 1. Gupta et al (2017) | India (November 2007-08 & December 2009-10) | Cross- sectional survey | Adolescents 6-14 years (n=214) | Anaemia (WHO) <12 gm/dl; serum iron and transferrin saturation | Nutritional status | Anaemia (49.7%) | Hospital based study,  Majority was IDA |
| 1. Jeyakumar et al (2018) | India (2005-2016) | Systematic Review and Meta-analysis | School going adolescent girls 10-19 years (n=1352) | Vit D deficiency (25(OH)D <50 nmol/L) + one study <30 nmol/L*, | Nutritional status | Prevalence (25.7%) | Systematic review and meta-analysis  Mixed urban/rural, school and population based studies |
| 1. Kapil et al (2016) | India (2014-15) | Cross-sectional survey | Adolescent 6-18 years (n=626) | Vit D deficient (25(OH)D <20 ng/ml) and insufficient (20-29 ng/ml)  Food frequency questions | Nutritional status | Vit D deficient (93%) and insufficient (5.9%) | High altitude district (Shimla)  Community based study  65% were veg, Vit d deficient more common in these participants  Females had higher prevalence of Vit D deficiency |
| 1. Sethi et al (2017) | India (October-December 2014) | Cross-sectional survey | Adolescent girls 14-19 years (n=124) | Anaemia (WHO) (Hb <12g/dL); mild (10-11.9 g/dl), moderate (7-9.9 g/dl) and severe (<7g/dL) | Nutritional status | Baseline: Mild (40%), moderately (37%) and severe (2%) anaemia | Tribal district  Community involvement is missing in entire design of WIFS programme |
| 1. Kapil et al (2015) | India (2014) | Cross-sectional survey | School going adolescent girls 12-18 years (n=5430) | WHO criteria for Goitre (Grade 1 & Grade 2)  Urinary iodine concentrations  Inadequately iodised salt (>15ppm) | Nutritional status | Goitre rate (6.8%):  Inadequately iodised salt (44-59%)  Adequate iodine status among the studied population |  |
| 1. Khatiwada et al (2016) | Nepal (2014) | Cross-sectional survey | School going children 6-12 years (n=227) | Iodine deficiency (UIE <100 ug/l)  Anaemia (WHO age specific cut offs) and iron deficiency (transferrin saturation cut-off < 16): | Nutritional status | Iodine deficiency (19.8%); Anaemic (35.24%); iron deficient (43.61%) |  |
| 1. Kumari et al (2017) | India (April 2015-October 2015) | Cross-sectional survey | Adolescent girls 10=19 years (n=200) | Anaemia (<12 gm/bl): mild (10=11.9 gm/dL); moderate (9-9.9 gm/dl); severe (<8 gm/dl) | Nutritional status | Anaemia (50%); mild (43.3%), moderate (3.3%) and severely (3.3%) | Hospital based study |
| 1. Mandlik et al (2018) | India | Cross-sectional survey | School going children 6-12 years (n=359) | Vit D insufficient (50-74.9 nmol/L) and deficient (<50 nmol/L)  Three-one-day dietary recall | Nutritional status | Vit D insufficient (71%) and deficient (24%)  34% consumed calcium RDA | Government school  Pune |
| 1. Rahman et al (2016) | Bangladesh (2011-12) | National Micronutrient Survey- Cross-sectional survey | Adolescents 6-14 years (n=1266) | Vit A: Subclinical deficiency (<.7 umol/l), mild (=>.7-<1.05 umol/l); moderate (=>.35-<.7 umol/l); severe (<.35 umol/l) | Nutritional status | Subclinical (20.8%), mild (53.5%), moderate (20%) and sever (.5%) Vit A deficiency  27.1-46% meet Vit A RDA – bioavailability  Intake animal source food associated with higher vit A levels | National micro-nutrition surveys 2011-12  Serum retinal level adjusted for infection  Slum areas severe deficiency (2.4%)  79% of RDA was plant origin  Of subclinical Vit A has not changed over the past decade  Fortification of all probable food items combined is considered in Bangladesh, along with dietary sources, intake of vitamin A would reach the tolerable upper level only for PSAC |
| 1. Mohsin et al (2016) | Pakistan | Cross- sectional descriptive survey | School going children 6-14 years (n=353) | Anaemia children 8-11 (Hb <11.5g/dl) and 11-14 years (Hb <12g.dl); IDA (ferritin <15yg/l) | Nutritional status | Anaemia (37.11%); Iron deficient (62.61%); IDA (28.9%) | Significant correlation with intellectual activities and school performance of the children |
| 1. Pareek et al (2018) | India, Jaipur (July 2015-January 2016) | Cross- sectional survey | Adolescent girls 12-15 years (n=472) | Anaemia (<12g/dl): mild (10-<12 g/dl), moderate (7-<10 g/dl) and severe (<7 g/dl) | Nutritional status | Anaemia (73.73%); mild (51.27%), moderate (19.91%) and severe (2.54%) | Slum area  Majority from urban slum, remainder rural areas.  Most were not receiving food from National Program (e.g. mid-day-meal).  Majority vegetarian  27.33% receiving IFA |
| 1. Rahman et al (2016) | Bangladesh (2011-2012) | Cross- sectional survey | Adolescents 6-14 years (n=1321) | Anaemia 6-11 years (<11.5 gm/dl) and 12-14 years (<12 gm/dl); iron deficiency (serum ferritin <15 ng/ml) | Nutritional status | Anaemia 6-11 years (19.1%) and 12-14 years (17.1%); Iron deficiency 6-11 years (3.9%) and 12-14 years (9.5%); iron deficient anaemia 6-11 years (1.3%) and 12-14 years (1.8%)  Overall 6-14 years: Iron deficiency (7.1%); anaemia (20.6%) | National micro-nutrient survey 2011-12  Area’s with ‘predominantly high groundwater Fe’ was a determinant of higher serum ferritin levels  Food security was associated with higher HB levels  Compared to rural areas, urban areas had lower level of ferritin, but higher level of household expenditure was significantly higher in urban children, but lower usage of water from tube wells for drinking |
| 1. Rakesh et al (2017) | India (2016) | Systematic review | School aged children (n=9061) | Anaemia: DHLS (<11g/dl for all age groups) and studies (WHO criteria) | Nutritional status | Studies (2003-14): Males (67%) and females (19.1%-77.5%)  Adolescents girls: DHS 2002-04 (90.1%) and 2012-13 (31.3%) | Systematic review (Kerala state)  Proportion of anaemia in school children was less in recent studies, giving the impression that anaemia is declining – but no conclusive data  Higher among tribal children |
| 1. Sarna et al (2020) | India (February 2016 and October 2018) | Comprehensive National Nutrition Survey- Cross- sectional survey | Children 5-19 years (n=28 964) | Anaemia: WHO age and sex specific cut offs;  WHO cut offs were also used to define ferritin, folate, and vitamin B12 deficiencies  Iron deficiency: ferritin <15 ng/mL plus folate =>151 ng/ml and Vit b12 >=203 pg/mL | Nutritional status | Anaemia: 5-9 years (23.4%) and 10-19 years (28.4%)  among anaemic: Iron deficient anaemia 5-9 (16.3%) and 10-19 (22.25) anaemia; Folate or b12 (24.2-25.6%); Dimorphic anaemia (11.7-19%); other causes (47.8-33.3%) | Nationally Nutrition Survey 2016-18  Anaemia prevention efforts should focus on strengthening the existing iron and folate supplementation programmes |
| 1. Majid et al (2017) | Pakistan (January 2013-December 2014) | Observational study | Children 6-16 years (n=175) | Zine deficiency (<65ug/dl) and toxicity (>150ug/dl) | Nutritional Status | Deficiency (54%) and toxicity (12.2%) | Wheat flour fort scheme (2005) – at a pop level decrease in Zn deficiency after fortification prog |
| 1. Nunn et al (2019) | India (January 2006) | Cross sectional survey | Adolescents girls 16-18 years (n=188) | Food frequency questioner | Nutritional Status | >RNI: Calcium (0); Iron (0); Zin (0); Vit A (3.2%); Vit C (43.6%)  >EAR: Calcium (1.1%); Iron (12.2%); Zinc (34.6%); Vit A (11.7%); Vit C (73.4%) | Mumbai slums  Half of the adolescents were not meeting the EAR for 10–14 micronutrients.  Medium intake below RNI for all micro-nutrients |
| 1. Amarasinghe et al (2017) | Sri Lanka | Cross sectional survey | School going children 5-11 years (n=4412) | Anaemia (Hb <12 g/dl) | Nutritional Status | Anaemia (11.8-20.2%).  among 126 anaemia children 18.5% iron deficient and 32.5% early iron deficiency. | No diff between males/females  Significantly associated with wealth, diet, BMI, age. |
| 1. Dutta et al (2020) | India (December 2016-Febuaey 2017) | Cross-sectional study | School going children 6-14 years n=1764) | Anaemia (Hb < 11 gm/dl) | Nutritional Status | Anaemia (72%) | Rural areas  Associated with disadvantaged castes, 51% received and appropriately consumed IFA tablets |
| 1. Rai et al (2020) | Nepal (2006, 2011 and 2016) | Demographic Health Survey- Cross-sectional study | Adolescents girls 15-19 years | Anaemia (<120g/L) | Nutritional Status | Anaemia: 2006 (8.8%); 2011 (8.3%); (2016 (8.8%) | Prevalence of anaemia remain stagnant  DHS data |
| 1. Jayatisaa et al (2020) | Sri Lanka (2000-2016) | National iodine surveys- Cross sectional design | School going children 6-12 years (n=16 910) | Presence of goitre  UIC: adequate 100-299 ug/L; excessive (-=>300 ug/L); sufficiency -<20% samples should have mUIC <50 ug/L | Nutritional Status | Significant reduction in the percentage of schoolchildren with mUIC < 50 µg/L (2.7% in 2000 vs 1.6% in 2016)  Goitre: reduce from 18% in 2000 to 1.9% in 2016 | NIS data – National Iodine Surveys  UIC was consistently in the adequate or iodine-sufficient range in all four national iodine surveys of 2000–2016  Regional differences – sig higher in North and northern central proveniences  Sig increase in percentage of salt adequately iodised (2005 43.5% v.s. 15% 2016) – presence of over iodised salt also fell with this period  USI was first implemented in Sri Lanka in 1995 |
| 1. Shrestha et al (2018) | Nepal (March-May 2015) | Cross sectional survey | School going children 8-16 years (n=708) | Anaemia (age-specific WHO cut offs) | Nutritional Status | Anaemia (23.6%) | No difference among sexes  Intestinal parasites common among these children |
| 1. Bansal et al (2016) | India (January 2012-March 2013) | Secondary analysis of the haemoglobin values from a randomized controlled trial (RCT) | Adolescent girls 11-18 years (n=888) | Anaemia (<120 g/l); mild (110-199 g/l); moderate (80-109 g/l) and severe (<80 g/l) | Nutritional Status | Anaemia (59.6-78.2%); mild (33.2-33.8%), moderate (24.8-43.4%) and severe (1-1.7%) | Slum areas  Used both direct and indirect cyanmethaemoglobin method |
| 1. Tamang et al (2019) | Nepal (2015-16) | Cross-sectional study | School going children 6-12 years (n=213) | Iodine deficiency: (WHO Classification – UIC)  Excessive iodine nutrition (=>300 ug/L) | Nutritional Status | Iodine deficiency (31.8%): Severe (.5%), moderate (3.8%) and mild (13.1%); excessive Iodine nutrition (9.9%) | Altitude effected iodine nutrition |
| 1. Rodrigo et al (2017) | Sri Lanka (June 2009 and July 2010) | Cross-sectional study | School going children 11-19 years (total n=7526) | Iron deplete (serum ferritin <15 ng/Ml); anaemia (WHO age/sex specific cut offs); IDA (Serum ferritin < 15 ng/ml and transferrin receptor > 28.1 nmol/l and Hb < 11.5.0 g/dl in children < 12 years, Hb < 12.0 g/dl in females ≥12 years and males aged 12–14 years and Hb < 13.0 g/dl in males aged ≥15 years) | Nutritional Status | Iron depleted (37%); Anaemia (25.2%); IDA (12.4%) | In participants with normal haemoglobin genotype (over 1000) |
| 1. Shetty et al (2018) | India, Karnataka (January 2017) |  | School going children 6-12 years (n=2703) | Goitre (WHO); salt samples (n=543); UIE (n=270) | Nutritional Status | Goitre (9.3%): grade 1 (7%) and grade 2 (2.3%)  Among participant examined UIE 4% had UIE <100 mcg/dl, none had goitre | Females and older children had higher prevalence  69.8% of salt adequately iodised (>15ppm); low prevalence of inadequate salt iodisation among children with goitre suggesting other contributing factors – deficiency in other nutrients like iron, vitamin A or selenium |
| 1. Pullakhandam et al (2021) | India (CNNS data) | Cross-sectional survey | Pre-school children (1-4 years, n=7874), school going children (5-9 years, n=10 430), adolescents (10-19 years, n=10 140) | International Zinc Nutrition Consultative Group (IZiNCG) Low serum zinc concentration cut offs-  Children <10 y (<65mcg/dl irrespective of fasting status, girls >10 y (70mcg/dl with morning fasting sample), boys >10 y (74 mcg/dl with morning fasting)  Or  Lower alternative cutoffs  Children <10 y (<59 mcg/dl independent of fasting status)  Girls <10y (<66 mcg/dl for morning non fasting)  Boys >10y (<70 mcg/dl) with morning non fasting) | Nutritional status | Low SZC with IZiNCG in adolescents (31.1%), School age children (15.8%), preschool children (17.4%) | The prevalence of low SZC was higher among rural preschool children, those belonging to households with poor socioeconomic status, and those with severe stunting or underweight.  Preschool children with diarrhea, productive cough,  or malaria/dengue in the 2 wk preceding the survey had a higher prevalence of low SZC than those without morbidity. |
| 1. Kulkarni et al (2021) | India (CNNS data) | Cross-sectional survey | Children and adolescents (1–19 y) (1–4 y: n=9635; 5–9 y:  n=11,938) and adolescents (10–19 y; n=11,507) | Anaemia using WHO cut offs, Iron deficiency with serum ferritin level (<12 mcg/dl in 1-4 y and <15 mcg/dl in 5-19 y) | Nutritional status | Anaemia in preschool children (40%), 5-9y (24%), and adolescents (29%)  Iron deficiency in preschool children (31.9%), 5-9 y (15.5%), and adolescents (20.9%) | In all age groups, ID  prevalence was higher in urban than in rural participants (1–4 y: 41% compared with 29%) and in those from richer quintiles (1–4 y: 44% in richest compared with 22% in poorest), despite adjustment for relevant confounders. |
| 1. Venkatesh et al (2021) | India (July 26, 2020) Systematic review | Systematic review and meta-analysis | Infants <5 years, Adolescents <18 years, adults <18 years and pregnant women | Iodine deficiency in <18 years, iron deficiency in 5-18 years, Folic acid deficiency (0-18 years), vitamin A deficiency (>5 years), vitamin D deficiency (0-18 years) | Nutritional status | Iodine deficiency (11%), Folic acid (39%), Vitamin A deficiency (13%), Vitamin D deficiency (60%), Iron deficiency (53%) |  |
| 1. Kamble et al (2021) | India, Delhi (October 2019) | Cross- sectional study | Adolescent girls 10-19 y (n=203) | WHO Haemoglobin cut offs; WHO 2007 reference standard BAZ classification | Nutritional status | Anaemic (59%); Among 119 anaemic girls, mild anaemic (48%), moderately anaemic (45%), severe anaemia (9%) | Anaemia among adolescents who took deworming tablet was significantly lower than their counterpart.  Adolescents who were underweight and vegetarian had significantly higher prevalence of anaemia than their counterparts. |
| 1. Scott et al (2022) | India (CNNS 2016-18) | Comprehensive National Nutrition Survey- Cross sectional study | Children and adolescents 10-19 years (n=14,673) | WHO Haemoglobin cut offs; FFQ | Nutritional status | Overall anemia (28.5%); mild anaemia (17.6%), moderate anaemia (10.0%), severe anaemia (0.9%)  Anemia in girls (39.6%) and boys (17.6%)  Prevalence varied by regions: South (29.0%) and East (45.8%) for girls; South (10.8%) and Northeast (28.4%) for boys  Prevalence varied by state: girls (7%–62%) and boys (4%–32%). | Iron deficiency (ferritin < 15 μg/L) was the strongest predictor of anaemia, followed by haemoglobinopathies, vitamin A deficiency and zinc deficiency (serum  zinc < 70 μg/L).  Higher odds of anaemia in Adolescents aged 15–19 years compared with those aged 10–14 years and girls were more likely to be anaemic than boys. |
| 1. Siddiqee MH et al (2022) | South Asia (Systematic review, 1 January 2001 to 31 December 2019)  Pakistan, Afghanistan, Bangladesh, India, Nepal, Sri Lanka | Systematic review and meta-analysis | Children and adolescents 0 to 18 years  6-18 years (n=12709 from Nepal and India) | Vitamin D deficiency or  Insufficiency- < 20 ng/  mL | Nutritional status | Prevalence in 6-18 years (57%) |  |
| ***Dietary patterns and quality*** | | | | | | | |
| 1. Som et al (2016) | India (March 2007 to February 2009 and august 2010 to March 2012) | Cross-sectional survey | Adolescent girls 14-19 years (2010/12 n=282) | Food frequency questionnaire | Nutritional status | 37.5% on a weight reduction diet and 74.1% felt dissatisfied with their own body weight.  Regular intake of vegetables (67%), pulses (79.4%), fish (51.4%), meat (12%) – occasional (80.4%), fruit (53.9%), milk (30.8%), fried foods (12.7%) – rare/never (44.6%) | Later study – school based  Self-reported anthropometry, not detailed  Food consumption and weigh perception differ by region  Nutrition transition |
| 1. Unisa et al (2020) | India (2016) | Prospective, non- randomized controlled evaluation | Adolescents girls 10-19 (n=6023) | MDD-W: low (1-3 DDS), medium (4-6 DDS) and high <6 | Nutritional status | Low (24.4%), medium (69%) and high (6,6%) DDS. Consumed pulses (60.6%), nuts (8.7%), milk (17.8%), meat etc (28.2%), eggs (9.6%), dark green veg (39.6%), vit a rich fruit/veg (74.7%), other veg (82.4%) and fruit (21.2%) | DDS varied across states.  Tribal adolescents has lowest DDS.  DD better for adolescent who consumed food from home gardens.  Older adolescents were likely to have lower DDS compared to younger adolescents |
| 1. Thapa et al (2017) | Nepal (2016) | Cross sectional survey | School going adolescents 12-16 years (n=1160) | CDC’s Youth Risk and Behaviour Surveillance System survey | Nutritional status | Gone hungry in the last month: sometimes (30.5%), most of the time (25.8%), always (13.9%).  Consumption of fruit (59.5%) and veg (71.4%) >1time/day, meat on day a week/less (71.5%) |  |
| 1. Tariq et al (2019) | Pakistan (February-April 2017) | Cross sectional survey | School going adolescent 13-19 years (n=226) | Consumption health/unhealth food via especially-designed proforma | Nutritional status | Consumption sweet snack (85.4-86.9%), salty snack (72.9-92.3%), fried foods (95.8-96.2%), soft drinks (89.6-84.6%), fast food meals ((68.8-81.5%), fresh fruit (80-90.6%) and veg (75.4-94.8%) | Significantly increased consumption of salty snacks, fast food, alongside decreased consumption of energy drinks, fruit and veg in females compared to males |
| 1. Ani et al (2016) | Pakistan (2009) | Global School Health Survey- Population-based cross-sectional survey | School going adolescents 13-15 years (n=4722) | Fruit and veg consumption | Nutritional status | Consumption of fruit and Veg >=5 times/day (10%); low per capita availability | High production and exportation of fruit and veg – food systems  Review |
| 1. Gupta et al (2018) | India | Cross sectional survey | School going adolescent 12-18 years (n=425) | Dietary patterns - questioner | Nutritional status | 36% consumer junk food in the past 24hrs; most popular was chips (71%) followed by chocolate (14%), bakery products (13%), soft drinks (7%), and sugar‑sweetened beverages (5%); junk food consist of 9.2% and 20.9% of calorie and fat content in the diet | No difference in consumption of junk foods between genders |
| 1. Islam et al (2020) | Bangladesh (September 2017-2019) | Cross sectional survey | Adolescents approximately 15 years (n=2463) | 24 hr recall – MDD (10 food groups FAO guidelines) | Nutritional status | Achieved MDD (58%); Apart from starchy staples, consumption proportion exceed 50% of only two food groups (veg and fish); Vit A-rich vegetables, tubers, and fruits were consumed by less than a third of the participant; expect fish animal source foods/product consumption was notably low | Rural areas  Nested in MiniMat trial - Mother received micro-nutrients intervention during pregnancy  Inadequate DD was prevalent across all socio-demographic strata – gradient from less to more affluent HH  Except for fish, consumption followed a socioeconomic gradient |
| 1. Rathi et al (2017) | India (December 2015 and April 2016) | Cross- sectional survey | School going children 14-16 years (n=1026) | Food frequency questionnaire | Nutritional status | Energy-dense snacks was the most commonly consumed food group (In decreasing order of average daily serving intake were cereals, energy-dense beverages, vegetables, fruits, milk and milk products, non-vegetarian food products, and pulses and legumes; half (52%) refrained from eating non-vegetarian food products and a similar proportion did not consume any fruit. | More females consumed cereals, vegetables, fruits and non-vegetarian food products than their male counterparts, but no difference in energy dense snacks.  Overconsumption of energy-dense, nutrient-poor foods and under consumption of vegetables, pulses and animal foods.  India is the largest producer of milk in the world, however, nearly two-fifths (36%) of the participants did not report any consumption.  Note 9 food groups were: cereals, pulses and legumes, vegetables, fruits, milk and milk products, non-vegetarian food products, energy-dense snacks, energy-dense beverages, and water. |
| 1. Shaikh et al (2016) | India (January-April 2012) | Cross- sectional survey | School going adolescents 13-16 years (n=399) | Food frequency questioner | Nutritional status | Compared with the Dietary Guidelines of India, adolescents consumed fruit 51·4 %, GLV 45·7 %, non-GLV 32·9 % and dairy 28·6 % less frequently and energy-dense foods more frequently than recommended | Public and private schools  More frequent consumption of processed foods among public-school students- high availability of low-cost versions  Girls reported more frequent consumption of packaged and ready-to-eat foods, non-green leafy vegetables and added fat than boys |
| 1. Sharma et al (2020) | India | Cross- sectional survey | Adolescent girls 10-19 years (n=223) | 24hr dietary recall | Nutritional status | Meet <70% of RDA: energy (35%); protein (45.7%); fat (54.2%); iron (12.1%); calcium (14.4%); zinc (17%); folic acid (29.2%) | Population based – 4 districts |
| 1. Fan et al (2020) | Afghanistan (2014); Bangladesh (2014); Maldives (2014); Nepal (2015); Pakistan (2009); Sri Lanka (2016) | Global School-Based Student Health Surveys (GSHS)- Cross-sectional survey | School going children 12-15 years (Afghanistan n=1319, Bangladesh n=2597, Maldives n=1676, Nepal n=4400, Pakistan n=4860, Sri Lanka n=2194) | Low FV = fewer 5 per day over the past 30 days; fast food consumption once per week; soft drink consumption once per day for the past 30 days | Nutritional Status | Afghanistan (Low FV 85.2%, FF 63.4%, soft drinks 40.7%); Bangladesh (Low FV 83.6%, FF 53.5%, soft drinks 47.8%); Maldives (Low FV 90.2%, FF 34.6%, soft drinks 32.5%); Nepal (Low FV 90.7%, FF 75.2%, soft drinks 33.4%); Pakistan (Low FV 90%, FF 20.9%, soft drinks 36.5%); Sri Lanka (Low FV 75.8%, FF 42.7%, soft drinks 26.7%) | Global School-Based Student Health Surveys 2009-2017  Clustering of poor dietary habits |
| 1. Shahbaz et al (2016) | India | Cross-sectional analytical study | School going children 12-14 years (n=385) | Food frequency questioner – high consumption <7 times/week | Nutritional Status | High consumption of soft drinks (33.25%), fruits (39.48%) | Private schools |
| 1. Aurino et al (2017) | India (2006-2013) | Longitudinal  study | Adolescent 5-15 years (n=2891) | 24 hr recall: DD 10 food groups - (i) cereals, (ii) starchy roots; (iii) legumes; (iv) milk and dairy products; (v) eggs; (vi) meat; (vii) fish; (viii) oil and fats; (ix) fruit; and (x) vegetables | Nutritional status | Mean DD (4.24-4.36); majority consume cereals, oil and vegetables, with substantial variation with regard to the consumption of animal-source foods or fruits, root vegetables and legumes | Young lives cohort  Pro-boy gaps exist at most ages with the exception of early adolescents.  Disparities between mid-adolescent boys and girls are driven by the increased likelihood of boys to consume protein- and vitamin-rich foods.  Disparities between mid-adolescent boys and girls are driven by the increased likelihood of boys to consume protein- and vitamin-rich foods |
| 1. Aziz et al (2018) | Pakistan (October-December 2016) | Cross sectional survey | School going children mean age 9 years – Grade 2-5 (n=1017) | Food frequency questioner | Nutritional Status | Eat breakfast regularly (75%) and never (10.3%); ate snacks at either morning, evening, bedtime regularly (43.9-54.1%); majority ate meat, chicken, fish eggs, nuts/seeds/pulses and fruits rarely; 64% ate junk food regularly | Regularly 6-7 day/week  Occasionally 3-5 days  Never/rare 0-2 days  Rural areas higher portion skipped breakfast (also related socio-economic)/snacks, higher consumption milk, lower consumption of fruit/animal protein/junk food.  Boys-higher portion skipped snacks, higher consumption of milk protein, lower consumption of animal protein/junk food |
| 1. Aurino et al (2016) | India (2006-2013) | Longitudinal study | Adolescents born 1995/6 (n=1914)and children born 2001/2 (n=994) | 24hr recall - Dietary diversity | Nutritional Status | DD remined stable over 2006-13, but changed in composition – Younger age groups more likely to consume eggs, milk products | Young Lives Study  No gender or rural/urban disparities but wealth disparities present  Sugar consumption decreased in urban but increased in rural areas |
| 1. Humphries et al (2017) | India (2002-2016) | Longitudinal study | Adolescents 5-12 years (n=1775 at 12 years) | Dietary diversity (7 food groups) | Nutritional Status | DD (3.84-4.49) | Increase in dietary divert from 5-18 years  Young Lives Cohort  DD, females and rural residence = predictors of HAZ  HH food expenditure ass with increases in HAZ – however how hh allocate spender if an important factor in India  Meat/fish expenditure lowest in India  Meat expenditure increased with wealth – stable for fruit/veg |
| 1. Li et al (2020) | Afghanistan (2014), Pakistan (2009), Bangladesh (2014) | Global School-Based Student Health Survey- Cross sectional survey | School going children 12-15 years (Afghanistan n=1490, Pakistan n=4988, Bangladesh n=2720) | Food frequency questioner | Nutritional Status | Fast food consumption: Afghanistan 2.4 days, Pakistan 1.4 days & Bangladesh 2.4 days/week | Global School Based Student Health Survey  Pakistan is one of the first developing countries to formulate a comprehensive National Action Plan for Non-Communicable Disease Prevention, Control and Health Promotion |
| 1. Khongrangjem et al (2017) | India (Agust-November 2016) | Cross sectional survey | School going students 15-18 years (n=158) | Fast food consumption in the last month | Nutritional Status | 41.9% of the participants reported that they eat fast food once a months and 18.7% reported that they had fast food twice a week; 72.5% reported eating fast food for the taste | 26.25% of participant had adequate knowledge of fast food; females had better level of knowledge |
| 1. Rathi et al (2018) | India | Cross sectional survey | School going adolescent 14-16 years (N=1026) | Dietary patterns | Nutritional Status | 58.8% stated that they were allowed to eat foods of their choice at home; 64.6% reported that they did not have a family food rule prohibiting television viewing during meal time; 76.7% revealed that vegetables were regularly served at dinner; Salty snack foods like potato chips (38.0%) and soft drink (36.2%) were the least regularly available food items in homes | Private schools  On the whole food rules did no differ but gender but a higher proportion of boys (59.3%) than girls (48.7%) reported that they were expected to eat all the foods served |
| 1. Talagala et al (2016) | Sri Lanka (2012) | Cross-sectional study | Adolescents 15-19 years (n=542) | Dietary patterns | Nutritional Status | 61 % spent their pocket money at least once/week on packaged snacks; most frequently consumed packaged food snacks were biscuits (85.4 %), instant noodles (66 %) and chocolates (61 %), while the bottled/packaged drinks were colas (76.9 %), milk (70.7 %) and fruit juices (26.4 %) | Over 74 % paid attention frequently to the brand name (75 %), price (85 %) and nutrition panel (81 %); The majority (84 %) had good knowledge (obtaining more than the 75^th^ percentile mark) on interpreting labels; 26.9% had good interpretation of % RDA |
| 1. Shridhar et al (2016) | India & Bangladesh (November 2011-March 2013) | Cross-sectional study | Children 5-14 years (India Chennai n=146, India Goa n=218, Bangladesh n=200) | Dietary intake | Nutritional Status | Fruit and vegetable consumption was low across all sites (87.8 % consumed <5 servings/day); processed food intake =>1 per/day Chennia (34.9%), Goa (10.1%); Bangladesh (44%) |  |
| 1. Kumar et al (2017) | India (January-June 2014) | Cross-sectional study | School going children mean age 14.2 years (n=1652) | Dietary patterns | Nutritional Status | Snack per day <= three (48.9%) four times (41.9%); skipping meals never (44.8%) and sometimes (42%); eating street shop/restaurant sometimes (53.3%) | Males had higher prevalence of snacking/number of meals  Older students and those belonging to higher socio-economic status were less likely to demonstrate good eating habits  Students who perceived themselves as overweight were less likely to take a high number of meals |
| 1. Bansal et al (2021) | India, UP and Bihar (UDAYA study- Wave 2) (2018-19) | UDAYA Study- Cross-sectional survey | Adolescents 10-19 years (n= 4221 male and 5787 female) | 24-hour recall; FAO 2016 Minimum dietary diversity score- intake of >=5 food groups (out of 10 food groups) in the past 24 hours | Nutritional status | Grains, white root tubers and plantains (~100%), dairy products (~80%), fruits (65%), nuts and seeds (26.8% in 10-14 y, 28.9% in 15-19 y), meat, poultry and fish (15% in 10-14y, 16% in 15-19y), eggs (14% in 10-14 y, 15% in 15-19 y)  Bihar (MDD- 61%), UP (MDD- 57%) | Males consumed more dairy products, nuts and seeds, Meat, Poultry and fish, eggs than females, while female consumed more fruits  than males.  Significant difference in the MDD by their socio-economic characteristics (age, education of self and mother, caste, place of residence, wealth etc)  No differentials were found among young and old adolescents |
| 1. Greksa et al (2017) | Bangladesh (2001) | Cross-sectional study | Children 7-14 years (n=289) | 24 hr recall | Nutritional Status | Three meals (78-66.2%); % achieve RDA for energy (60.3-72.2%); calcium (16.1-22.7%); vitamin A (33.5-43.6%); nearly all achieved protein and iron RDA | Street and slum children  No difference in meal patterns between genders  Boys had higher %RDAs than girls for protein, calcium, iron, thiamine, and niacin, after controlling for group and age  Boys had greater dietary diversity than girls |
| ***Multiple measures of nutritional status*** | | | | | | | |
| 1. Acharya et al (2016) | Nepal (October-December 2013) | Cross sectional survey | School going adolescent 16-19 years (n=838) | BAZ: Cut off value of 85th percentile was used for classification of overweight, 95th percentile for obesity and < 5th percentile for thinness. | Nutritional status | Thinness (15.27%); normal weight (76.61%); overweight (5.85%); obese (2.27%) | The study reported high underestimation among obese (94.7%) and overweight adolescents (55.1%)  and high overestimation among thinness adolescents (64.1%).  Overweight and obesity among adolescents wasn’t significantly associated with knowledge of obesity – nutrition education compulsory in Nepal curriculum. |
| 1. Adhikari et al (2020) | Nepal (2016) | Secondary data analysis (Demographic and  Health Survey) | Adolescent age 15-19 years (n=170) | Thinness (BMI less than 18.5); overweight/obesity (BMI of 25 or more); anaemia (haemoglobin level of less than 11 g per decilitre). | Nutritional status | Thinness (26.8%); overweight (4.7%); anaemic (40.5%) |  |
| 1. Rose-Clark et al (2019) | India (June 2016 to January 2017) | Cross sectional survey | Adolescent girls aged 10-19 years (3324) | Stunting (HAZ <-2 SD); thinness (BAZ <−2 SD; overweight BMI >1 SD; Mid-upper arm circumference (MUAC) cut off 10-14 years <160mm; FANTA MDD | Nutritional status | 10-14 years: Thinness (14.3%); overweight (1.9%); stunting (33.1%); MUAC (3.6%); MDD (23.8%)  15-19 years: Thinness (6.4%); overweight (1.1%); BMI <18.5 (40.8%); stunting (58.2%); MDD (21.5%)  Total: Thinness (10.7%); overweight (1.5%); stunting (44.8%); MDD (22.7%) | Prevalence of thinness among younger girls was more than twice the prevalence among older girls  Large community-based study but largely tribal communities |
| 1. Cunningham et al (2020) | Nepal (June-September 2017) | Cross sectional survey | Adolescent girls ages 10-19 years (n=1093) | BAZ scores: Severe thinness (<= -3SD; moderate thinness (<=2SD); mild thinness (<=-1SD); overweight (>=-1SD); obese (>=2SD) WHO thinness (BAZ <=-SD)  WHO overweight (BAZ >=1SD) MDD-Women anaemia was tested by measuring haemoglobin concentrations, using HemoCue Hb-301 photometers. | Nutritional status | 10-14 years: Severe thinness (2.2%); moderate thinness (11.1%); mild thinness (31.8%); overweight (2.6%); obese (0.6%); WHO thinness (45.1%); WHO overweight (3.2%); MDD (31.2%); anaemia (17.0%)  15-19 years: serves thinness (1.0%); moderate thinness (6.8%); mild thinness (20.4%); overweight (2.6%); obese (0.3%); WHO thinness (28.2%); WHO overweight (2.9%); MDD (39.1%); anaemia (27.3%)  Adolescent mothers: Severe thinness (0%); moderate thinness (3.6%); mild thinness (15.0%); overweight (7.7%); obese (0.8%); WHO thinness (18.6%); WHO overweight (8.5%); MDD (32.4%; anaemia (28.6%)    Consumption of food groups  Grains, white roots and tubers, and plantains (100%); pulses (72.1-74.2%); nuts and seeds (2.7-5.9%); dairy (26.2-32.9%); meat, poultry, and fish (25.8-33.2%); eggs (2.3-7.8%); dark-green leafy vegetables (40.6%-41.8); Vitamin A–rich fruits and vegetables (9.2-12.05); Other vegetables (83.6-90.5%); other fruits (26.9-40.3%); sweet snack food (11.3-27.1%), instant noddle (17.2-29.7%0, sugar-sweetened beverages (3.9-6.5%) | Suaahara data: across 16 districts  Data consistent with Nepal DHS, first - in Nepal’s history, the proportion of overweight and obese women surpassed the proportion of thinness  No differences in dietary diversity.  Young adolescent had lower levels of exposure to information and key nutrition messages  There is yet sufficient evidence on the effectiveness of school gardens and other interventions, particularly at scale, for improving adolescent dietary behaviours |
| 1. Mistry et al (2017) | Bangladesh (October 2015-January 2016) | Nationally representative  cross-sectional survey | Adolescent girls 10-19 years (n=1269) | Anaemia (< 12 g/dl); mild anaemia (10.0– 11.9 g/dl); moderate anaemia (7.0–9.9 g/dl); severe anaemia (<7g/dL).  BAZ: Malnourished (< −2SD); over nourished (> +1SD) | Nutritional status | Anaemia prevalence in non-pregnant adolescents (52.9%) | National representative sample -BRAC  Anaemia prevalence was slightly higher among adolescent girls from urban slums |
| 1. Pawar et al (2020) | India (2014-2016) | Cross sectional survey | Adolescent 14-17 years (n=1135) | BAZ thinness (<5^th^ percentile); overweight (85^th^-95^th^ percentile); obese (>=95^th^)  Anaemia: females (Hb <12g/dl), males (Hb <13g/dl); mild females (Hb 11.0-11..9 g/dl), males (Hb 11.0-12.9 g/dl); moderate (Hb 8.0-10.9 g/dl; serve (Hb <8.0g/dl) | Nutritional status | thinness: 41.3% of tribal adolescents (Boys 48.0%; Girls 34.0%) and 48.0 % non-tribal of the same region.  Obesity almost null  Anaemia in tribal (41.4%) and non-tribal (19.3%) adolescents; mild tribal girls (21.3%) and boys (37.7%) and non-tribal girls (13.9%) and boys (13.4%); moderate boys (nl), girls tribal (17.4%) and non-tribal (10.25); severe tribal girls (2.2%) and boys (0.8%), and non-tribal girls (0.5%) and boys (0.6%) | Microcytic hypochromic anaemia is a major problem in tribal communities, after iron supplementation reduction in anaemia; this IDA still present |
| 1. Ahmad et al (2018) | Pakistan (August-September 2015) | Descriptive analytic study | School going children 3-10 years (n=478) | Using WHO growth standards: Overweight (HAZ =>+2SD), obese (HAZ =>+3SD), underweight (WAZ =<-2SD), severely underweight (WAZ =<-3SD), stunting (HAZ =<-2SD), and severely stunted (HAZ=<-3SD) | Nutritional status | Obese (0.8%); overweight (0.6%); underweight (11.1%) and severely underweight (4.6%); stunted (11.5%); severely stunted (2.5%) |  |
| 1. Akram et al (2017) | Pakistan (July 2014) | Cross sectional descriptive study | School going children 4-14 years (n=310) | BAZ; thinness (<5^th^ percentile); overweight (85^th^-95^th^ percentile); obesity (=>95^th^ percentile) | Nutritional status | Thinness (56.1%), overweight (4.1%) and Obese (0.6%) |  |
| 1. Azizi et al (2019) | Afghanistan (June to September 2016) | Cross- sectional analytical study | Adolescent 10-18 years (n=308) | 25-OH-D levels: Insufficiency (>20-29 ng/ml); deficiency (<20 ng/ml); severe deficiency (<10 ng/ml)  BMI: Overweight/Obese (>85^th^ percentile), thinness (<5^th^ percentile) | Nutritional status | Low levels of vitamin D (77.3%); insufficiency (16.2%); deficiency (26.3%); severe deficiency (34.7%)  Overweight/obese (27.6%); thinness (9.1%)  Calcium with Vitamin D supplementation (35.1%). | Hospital based study  Exospore to sunlight was not observed in  38.3% of participants.  Among participant who had sunlight exospore 44.2% only exposed face/hands  Association with diet, BMI, clothing, sun protector, darker skin and gender  Conducted peak season for Vit D deficiency |
| 1. Balaram et al (2017) | India (October 2012 – March 2015) | Cross sectional survey | School children 10-14 years (n=1748) | BMI: Thinness (<5^th^ percentile); Overweight (>85^th^ percentile) | Nutritional status | Boys: thinness (10.22%) and overweight (12.5%).  Girls: Thinness (5.21%) and overweight (6.1%) | Half tribal, half non-tribal students  Little rural/urban difference & tribal adolescent having better physical characteristic compared to non-tribal counterparts |
| 1. Bellizzi et al (2020) | India (2005 and 2015) | Secondary research | Adolescent girls 15-19 years (2015 n=3041) | Anaemia (<12.0g/dl)  BMI: thinness (<18.5) | Nutritional status | 2005: Anaemia: 61.7%-50% in poorest -richest wealth quintiles  Thinness 22%-14% in poorest -richest wealth quintiles  2015: Anaemia: 56.2%-38.9% in poorest -richest wealth quintiles  Thinness: 17.6%-12% in poorest -richest wealth quintiles  Overall: Odds of anaemia were significantly higher among adolescent compared with adult women | Used DHS  In 2015 anaemia and thinness significantly high in rural areas – not observed in 2005 data  Largest improvements in wealthiest quintiles  India highest rate of anaemia in the world – diet  UNICEF “Adolescents, Diets and Nutrition: Growing Well in a Changing World” report revealed that the governmental nutritional schemes are not reaching the adolescents |
| 1. Bhargava et al (2020) | India (2005/6 -2015/6) | Secondary research | Adolescent (5-19 years (2015/6 n = 144 320) | Stunting (HAZ <-2SD); thinness (BAZ <-2SD); serve thinness (BAZ <-3SD); overweight (BAZ >+1SD); obese (BAZ >+2SD) | Nutritional status | Overall: Thinness (10%); Severe thinness (1.7%); stunting (34.1%); overweight (5.1%); obese (0.6%)  Boys: Thinness (16.5%); severe thinness (3.6%); stunting (32.2%); overweight (6.2%); obese (1.2%)  Girls: thinness (9%); serves thinness (1.4%); stunting (34.4%); overweight (4.9%); obese (0.8%) | National Family Health Surveys  Adult cut off overestimate –should consider adolescents as a separate age  Overall, in South Asia, the prevalence of thinness has been higher in boys |
| 1. Bhargava et al (2016) | India (June 2013- May 2014) | Cross sectional survey | School going children 6-16 years (n=1410) | 24-hour diet recall  BAZ: Thinness (<-2SD); overweight (BAZ >+1) | Nutritional status | Overall: Thinness (13.6%) and overweight (15.6%)  Boys: Thinness (14%) and overweight (18%)  Girls: Thinness (13.2%) and overweight (13.2%) | Statistically significant higher prevalence of overweight in urban and private schools  Caloric intake of urban participant higher compared to rural participants  Type of lunch eaten at school, habit of eating fruit (marker wealth) and eating out associated with nutritional status |
| 1. Bhuvaneswari et al (2020) | India, Madurai districts | Cross-sectional survey | School going adolescent 13-18 years (n=514) | Food choice questioner  BAZ: overweight (85^th^ – 95th percentile); obese (=>95^th^ percentile) | Nutritional status | Overweight/obese (6.71%); 45.51% of children eat watching TV compared to 39.04% having family meals; 74.4% of obese adolescents were found to have the habit of consuming snacks; no statistical sig relation found between BMI and snacking | Adolescents habitually choose more salty and crunchy snacks than the other healthier substitutes available |
| 1. Choudhary et al (2017) | India, Rajasthan | Cross-sectional survey | School going adolescent 12-15 years (n=1500) | BMI: thinness (<20); overweight/obese (BMI 25+) | Nutritional status | Overweight/obese (9.6%); thinness (60.7%) | Concern has been raised over the consumption of sugar-sweetened beverages |
| 1. Choudhary et al (2016) | India, Rajasthan (December 2011-May 2012) | Cross-sectional survey | School going girls 11-16 years (n=327) | Stunting (HAZ); BAZ (thinness-BAZ)  BMI: thinness (<18.5); overweight (23-24.9); obese grade 1 (25-29.9); obese grade 2 (>30)  Chronic energy deficiency: CED I (BMI 17-18.5), CED II (BMI 16-17) and CED III (BMI< 16)  24hr recall | Nutritional status | Stunting (21.2-26.3%); thinness - BAZ (27-18.9%); thinness <18.5 (73.7%); overweight (1-1.4%), obese (<1%); CED 1 (25.6%); CED 2 (19.9%); CED 3 (28.1%)  (89.6%) were pure vegetarians | Stat sig diff in stunting/thinness between rural/urban areas  Nearly one fourth of India’s population comprises of adolescents  Private schools |
| 1. Choudhuri et al (2020) | Bangladesh | Cross-sectional survey | School going children 14-18 years (n=893) | BAZ: Overweight (85^th^ -95^th^ percentile), thinness (<5^th^ percentile) | Nutritional status | Thinness (8.1%) and overweight (12.9%) | No sig rural urban differences in BMI |
| 1. Ramkumar et al (2018) | India, Pudcherry (November 2013 and January 2014) | Cross-sectional survey | School going children 7-15 years (n =792) | WAZ: underweight (<-2SD) and severe (<-3SD) underweight  HAZ: stunting (<-2SD) and severe (<-3SD) stunting  WHZ: wasted (<-2SD) and severe (<-3SD) wasting | Nutritional status | Underweight (38%), wasted (34%) and stunted (19%)  48.7% of children suffered from one or more anthropometric failure: Wasting only (6.4%), wasting and underweight (18.7%), wasting, underweight and stunting (8.6%), stunting and underweight (6.1%), stunting only (4.4%) and underweight only (4.5%) | Rural children  Wasting more prevalent among male children, no sig difference for stunting.  Undernutrition increased with age.  Using WAZ as the sole criteria for assessing the magnitude of undernutrition in this study would give us an underestimate of undernourished children of about 10% |
| 1. Sarma et al (2019) | India, Guwahati (2011-2013) | Cross-sectional survey | School going children 8-14 years (n=500) | Vitamin D: deficiency (25(OH)D <20 ng/ml); insufficiency (25(OH)D 21 to 29 ng/ml)  24hr recall  BMI: overweight (>25) and thinness (<18.5) | Nutritional status | Vit D deficiency (8.4%) and insufficiency (14.2%)  Overweight (1.4%) and thinness (73.26%) | Rural areas generally had higher sun exposure levels  Associated factors: calcium intake, hight, sun exposure  No difference between boys/girls  Short winter – little seasonal variations |
| 1. Zainab et al (2016) | Pakistan (May-October 2011) | Cross-sectional survey | Adolescents 10-14 years (n=385) | BAZ: Severe thinness (<-3SD); thinness (<-2SD); Overweight (>1SD-<2SD); obese (>2SD)  HAZ: Severe stunting (>-3SD); Moderate stunting (>-2SD-<-3SD); mild stunting (>-1SD-1SD) | Nutritional status | Severely thin (1.3%); Thin (8.1%); overweight (17.9%); Obese (3.6%), morbidly obese (1.6%)  Mild (18.7%) moderate (40.3%) and severe (31.4%) stunting.  Stunting increases with age | Child labours  Offered them good meal at meal time (88.3%); allowed the child to take rest (49.6%)  BMI might be inflated because of the decreased height of the children |
| 1. Young et al (2020) | India (2005/5 and 20015/16) | Secondary research | Adolescent girls 15-19 years (2016 n=117 722) | BMI z scores: thinness (<-1SD); thinness (<-2SD); overweight/obesity (>1SD) | Nutritional status | 2015: thinness (38.8%) and overweight (5.2%)  Decrease in thinness (42.7-38.3%), but increase in overweight (3%-5.2%)  Large variations cross states – states specific approaches | National Family Health Survey  Higher Socio-economic status, living in urban areas, improved diet diversity, higher education, religion and using an improved sanitation facility associated with obesity and thinness  Dual burdens of overweight and underweight, with divergent risk factors, greater alteration to the dual burden |
| 1. Yang et al (2020) | India, Ladakh (2012-2018) | Cohort study | School going children 3-18 years (n=401) | Underweight (WAZ <-2SD); wasting (WHZ <-2SD); stunting (HAZ <-2SD) | Nutritional status | Underweight (2012-18): 46.7%, 30.8%, 26.5%, 18.2%, 10.3%, 8.1%, and 3.6%  Stunting (2012-18): 44.6%, 40.7%, 33.3%, 27.5%, 26.9%, 24.3%, and 18.0%  Wasting (2012-18): 14.4%, 9.7%, 10.2%, 6.9%, 7.2%, 8.0%, and 9.0%. | Body weight and height increased gradually year by year  Cohort study |
| 1. Yang et al (2018) | Pakistan (2009), India (2007), Sri Lank (2008) | Secondary research | School going children 12-15 years – India (n=6132), Sri lank (n=2167), Pakistan (n=4808) | BAZ: thinness (<-2SD); overweight (>1SD) and obesity (>2SD)  BMI: thinness (<18.5 kg/m); overweight (=>25 kg/m); obese (=>30 kg/m) | Nutritional status | Pakistan: IOTF thinness (21.9%), overweight (5%), obese (0.8%); WHO thinness (8.8%), overweight (6.4%) and obese (1.3%)  India: IOTF thinness (27.8%), overweight (7.8%), obese (1.6%); WHO thinness (12.7%), overweight (10.5%), obese (4.2%)  Sri Lanka: IOTF thinness (48.8%), overweight (3.8%), obese (0.4%); WHO thinness (26.7%), overweight (4.5%), obese (0.9%) | Global School-based Student Health Survey  IOTF criteria notably higher prevalence of thinness – exceeding 15% frog India and Sri Lanka  No difference for obesity |
| 1. Caleyachetty et al (2018) | India (2007), Sri Lanka (2008), Pakistan (2009) | Secondary research | School going children 12-15 years | Stunting (HAZ <2SD); thinness (BAZ <2SD); overweight (BAZ >1SD); obese (BAZ >2SD) | Nutritional status | Pakistan: Stunting in boys (4.9%) and girls (10.6%); thinness boys (12.4%) and girls (9.2%); overweight/obese boys (5%) and girls (8.9%)  India: Stunting in boys (14.2%) and girls (15.1%); thinness boys (17.2%) ad girls (14.1%); overweight/obese boys (12.1%) and girls (9.8%)  Sri Lanka: Stunting in boys (30.4%) and girls (21.5%); thinness boys (39%) and girls (25.1%); overweight/obese boys (4.8%) and girls (4.8%)  Concurrent stunting and overweight or obesity: India (2.1%), Sri Lanka (0.8%) and Pakistan (0.6%) | Key principle: current actions designed to address one form of malnutrition do not unintentionally increase the risk of another.  Evidence-based double-duty actions can offer an integrated approach to addressing malnutrition, they warrant adaptation to different contexts  Global School-based Student Health Survey and Health Behaviour in School ages children survey |
| 1. Yadav et al (2018) | Nepal (July 2016-June 2017) | Cross-sectional survey | School going students 15-19 years (n=640) | Overweight (BMI =>25) | Nutritional status | Overweight (6.1%)  Fruit consumption >=5 days/week (72%), veg consumption >=5 days/week (53.7%) | BMI associated with ethnicity and urban residence |
| 1. William et al (2019) | Sri Lanka | Data from ‘Integrating Nutrition Promotion and Rural Development’ project, a quasi-experimental study | School going adolescent 12-18 years (n=1300) | Food frequency questionnaire; diet quality Index-International  BMI: overweight (25 kg/m^2^); obesity (30 kg/m^2^); thinness (BMI of 16, 17 and 18·5 kg/m^2^). | Nutritional status | thinness (35%): Grade 1 (18.24%), grade 2 (10.3%), grade 3 (6.73%)  Overweight (4.37%); obese (0.87%).  Suboptimal dietary intakes: carbohydrates provided over 70 % of energy, protein provided about 10 % | Males having significantly higher variety, adequacy and total DQI-I scores compared with females  Rural areas |
| 1. Verma et al (2020) | India, Punjab (march-August 2018) | Secondary data analysis | School going adolescent 5-18 years (n=897) | Mildly underweight (WAZ <-2SD) and severely underweight (WAZ <-3SD); Stunting (HAZ <-2SD); mild (<-2SD) and severe (<-3SD) thinness; overweight (BAZ >+1SD); Obesity (>+2SD) | Nutritional status | 5-9 years: Mildly underweight (25.6%); serve underweight (58.8%); mild stunting (34.4%); severe stunting (37.4%); serve thinness (31.8%); mild thinness (23.3%); overweight (0.3%)  10-18 years: mild stunting (33.9%); severe stunting (19.4%); serve thinness (26.9%); mild thinness (24.9%); overweight (0.1%) | Government/government aided schools  girls were affected more by undernutrition in middle childhood, i.e. during 5–9 years (both by stunting and thinness), but in middle and late adolescence, males were more stunted and thin compared to the females  Higher stunting in early adolescence (10–14 years) compared to late adolescence |
| 1. Yaya (2020) | India (2015-16) | Cross sectional survey | Adolescents girls 15-19 years (n=122 416) | BMI: thinness (<18.5 kg/m^2^); overweight (23–27.4); obese (>27.5 kg/m^2^ ) | Nutritional status | Thinness (31.7%); overweight (6%); obesity (3.3%) | National Family Health Survey – women childbearing age |
| 1. Wolf et al (2020) | Pakistan (2009); India (2011 & 2016); Bangladesh (2014) | Review | Adolescents 6-17 years Pakistan (n=284); India (2011 n=8401 & 2016 n=616); Bangladesh (n=10 135) | Underweight and obesity | Nutritional status | Pakistan: Underweight (52%), obesity (6%)  India 2011: Obesity increased from 9.8% to 11.7% (2006-09), while underweight decreased from 11.3% to 3.9%.  India 2016: 32% of the students were overweight or obese  Bangladesh: Obesity and overweight in students in urban (5.6%, 10.6%) and rural schools (1.2%, 8.6%, respectively); underweight in urban (16.1% ) and rural schools (19.2%) | Systematic review  Dual burden observed among Pakistani and Bangladesh school children  India: increase in obesity – most commonly males students attending private schools |
| 1. Warnakulasuriya et al (2019) | Sri Lanka | Cross sectional survey | School going children 5-15 years (n=12 788) | BAZ (WHO): Severe thinness (<-3SD); thinness (<-2SD); Overweight (>1SD-<2SD); obese (>2SD)  Stunting: mild (HAZ <-2SD) and severe (HAZ <-3SD)  Wasting (WHZ =<-2SD) | Nutritional status | Severe thinness (2.5-5.8%); overall thinness (17.9%); overweight (5.5%-12.9%); obese (2.8-6%)  Mild (3.4-7.6%) and severe stunting (0.3-1.1%)  Wasting (15-22%) | 19% of older boys and 27% of older girls were obese according to the level of FM.  IOTF and WHO obesity cut-off had poor level of case detection  Validity of internationally developed anthropometric cut-offs in South Asian children is unsatisfactory; hence, locally/regionally developed anthropometric tools should be used |
| 1. Tanwi et al (2019) | Bangladesh (2004, 2007, 2011 and 2014) | DHS- cross sectional survey | Adolescent 15-19 years (n=8803) | BMI: Thinness (< 18.5 kg/m^2^), overweight (25 ≤ BMI < 30 kg/m^2^) and obese (BMI ≥ 30 kg/m^2^) | Nutritional status | Thinness (32%) and overweight/obese (6.2%) | Used DHS data  Highest prevalence of thinness in women of repro age is in adolescents |
| 1. Campisi et al (2019) | Pakistan | Longitudinal cohort study | Adolescent 9-11 years (n=1872) | Stunting: mildly stunted (-2 <HAZ <-1), stunted (HAZ <-2); BAZ obese (>2), overweight (>1), thinness (<-2), severe thinness (<-3)  Anaemia WHO age/sex specific criteria: children 12-14 years and females 15+ years (mild (110-119 g/l), moderate (80-109 g/l), severe (<80)); men 15+ (mild (110-129 g/l), moderate (80-109 g/l), severe (<80g/l)  Vit A: serum retinal <.7 umol/L | Nutritional status | Mildly stunted (49.9-50.5%), stunted (34.9-33.3%), obese (0.1-0.6%), overweight (1.4-2.7%), thinness (0.7-1%), severe thinness (18.9-25.2%)  Anaemia (50%): mild (57.6-64.4%), moderate (41.6-33.3%), severe (0.1-0.2%)  High prevalence of Vit A deficiency | Karachi slum |
| 1. Ahmed (2017) | Bangladesh (July 2006-June 2007) | Cross sectional survey | School going children 10-18 years (n=386) | BAZ; Thinness (<5^th^ percentile); overweight (85^th^-95^th^ percentile); obesity (=>95^th^ percentile) | Nutritional status | Quarter performed moderate physical activity; 34% watched TV/used computer 3+hrs/day  Thinness (6.5%); overweight (16.3%) and obese (8.5%) | Children from affluent families |
| 1. Kumar et al (2019) | India | Cross sectional survey | School going adolescent 5-18 years (n=424) | BMI: Thinness (<18.5 kg/m); overweight (25 ≤ BMI < 30 kg/m^2^) and obese (BMI ≥ 30 kg/m^2^) | Nutritional status | Thinness (35.14%); overweight (8.49%); obese (2.12%).  Television for more than 2 hr (62.56%). 18.30% students were using the internet for more than 1 hour (18.30%); consuming fast food (37.70%) | Study – economically disadvantaged families |
| 1. Hambidge et al (2017) | India & Pakistan (2013-14) | Prospective observational study | Adolescent girls 15-18 years (India n=276 & Pakistan n=275) | BMI: Thinness (<18.5 kg/m); overweight ( ≤ 25kg/m^2^)  Stunting (HAZ) | Nutritional status | India: Thinness (17.9%) & overweight (11.8%); stunted (13.4%)  Pakistan: Thinness (17.2%) & overweight (6.6%); stunted (17.2%) |  |
| 1. Ali et al (2018) | Pakistan (February-August 2014) | Cross-sectional study | Children 5-14 years (n=500) | Food frequency questionnaire: BMI – thinness, overweight | Nutritional status | Thinness (68.8%); overweight (15.2%) |  |
| 1. Kajale et al (2016) | India | Cross-sectional survey | School going children 10-14 years (n=417) | 24 hour dietary recall: BAZ– overweight (>1SD) and obesity (>SD) | Nutritional status | Overweight (25%) & obesity (10%)  Satisfied RDA deity calcium intake (20%) | Affluent families |
| 1. Dhungana et al (2019) | Nepal (2015-16) | Global School Health Survey- Population-based cross-sectional survey | School going adolescent 13-17 years (n=5795) | BAZ (WHO): thinness (<-2SD); overweight (>1SD); obesity (SD>2SD) | Nutritional status | Overweight/obesity (5.23-7.04%); frit/veg intake <5 times/day (94.54-96.16%) | Nationwide survey – Global School Based Student Health Survey  Children with normal BMI in early age have a lower tendency to be overweight and obese by age 15 years and later, it is prudent to launch such behaviour change strategies in lower grade children |
| 1. Dolkar et al (2019) | India (February 2013-November 2014) | Cross sectional survey | School going children 10-19 years (n=616) | BIM-for-age (WHO): thinness (<-2SD); severe thinness (<-3SD) overweight (>1SD); obesity (SD>2SD) | Nutritional status | Obese (2.8%); overweight (12.8%); thinness (8.4%); severe thinness (3.4%) | Private and government schools  Malnutrition seen more in public schools |
| 1. Ekbote et al (2017) | India (June 2013-July 2014) | Cross sectional survey | School going children10-19 years (n=108) | 24 hr recall; stunting (HAZ); underweight (WAZ) | Nutritional status | Three primary patterns observed – mixed (MF - 49%), wheat and milk products (WMB-30%), rice and pulse patterns (RP-21%). Stunting in adolescent with WMB (10%), MF (28%), RP (29%)  80% did not meet calcium RDA  Overall: Stunting (19%) and underweight (28%) | MF lower energy and protein intake |
| 1. Faizi et al (2017) | India (August 2011-April 2012) | Global School Health Survey- Population-based cross-sectional survey | School going adolescents 13-15 years (n=1456) | BAZ (WHO): overweight (>1SD), obese (>2SD); thinness (<-2) | Nutritional status | Overweight (10.5-13.4%); obese (2.2-2.4%); thinness (2.6-2.9%) |  |
| 1. Ford et al (2020) | Nepal (2016) | National Micronutrients Status Survey- Cross-sectional nationally  representative survey | Adolescents 10-19 years (n=1135) | Anaemia (WHO): Boys and girls 10–11 years (Hb <11.5 g/dL); boys 12–14 years and girls 12–19 years (Hb <12.0 g/dL); boys 15–19 years (Hb <13.0). Anaemia severity: mild (boys and girls 10–11 years (11.0–11.4 g/dL), boys 12–14 years and girls 12–19 years (Hb 11.0–11.9 g/dL) boys 15–19 years (Hb 11.0–12.9 g/dL)); moderate (Hb 8.0–10.9 g/dL); severe (Hb <8.0 g/dL)  BAZ (WHO): thinness (<-2SD); overweight (>1SD)  Iron deficiency: ferritin <15 ug/L  Iron deficient anaemia: ferritin <15 ug/L and age/sex specific hemoglobulin levels  Vit A: RBP <0.64 umol/L | Nutritional status | Girls: Anaemia (20.6%); mild (12.62%), moderate (6.13%), severe (0.12%). thinness (12.80%); overweight/obese (4.64%), iron deficiency (17.56%), Vit A deficiency (1.79%), MDD (43.04%), IFA supplement (1.79%).  Boys: Anaemia (10.9%); mild (7.76%), moderate (1.34%), severe (0). thinness (22.03%), overweight/obese (3%), iron deficiency (3.72%), Vit A deficient (1.24%), MDD (46.43%), IFA supplements (1.34%) | Rural, urban and slum areas  Vit A retinol equivalent in text  One third anaemia girls were iron deficient  Food security also analysed  HO recommends intermittent IFA supplementation to all children aged 10–12 years and menstruating adolescent girls where anaemia prevalence is ≥20% - In 2016, the MoHP in Nepal began scaling up a program to provide weekly IFA supplementation to girls 10–19 years  High odds of anaemia in older girls; odds differed by ecological zone |
| 1. Galgamuwa et al (2017) | Sri Lanka (January-August 2014) | Cross sectional survey | Adolescents 6-15 years | (WHO): underweight (WAZ <-2SD); stunted (HAZ <-2SD); thin (BAZ <-2SD); overweight (WAZ >2SD); obesity (BAZ >2SD)  CIAF | Nutritional status | 6-10 years: underweight (48.9%), stunted (28.9%), thin (37.8%), overweight (2.8%), obese (2.8%), underweight & stunted (14.4%), underweight & thin (18.9%), thin & stunted (0), thin & stunted & underweight (11.1%)  10-15 years: underweight (24.2%), stunted (23.6%), thin (37.3%), overweight (4.3%), obese (4.3%), underweight & stunted (8.1%), underweight & thin (8.7%), thin & stunted (1.2%), thin & stunted & underweight (1.2%) | Secondary school children had the highest percentage of one type of undernutrition conditions  Two or more than two types of undernutrition conditions were more common among primary school children  As undernutrition increases with age, it reflects longer exposure to chronic malnutrition is common among children in this community  Plantation communities – relatively match national estimates |
| 1. Ganesan at al (2019) | India (2014-15) | Cross sectional survey | School going adolescents 10-18 years (n=1425) | BAZ (WHO): thinness (<-2SD); severe thinness (<-3SD) overweight (>1SD); obesity (SD>2SD) | Nutritional status | Severely thin (5.4%), Thin (17.8%), overweight (4.6%), obese (3.4%)  Habit of consuming green leafy vegetables (0%), protein rich foods (86%), mid-day snack (77.3%), eating junk food (84%), skipping meal (27.2%)  thinness was more common among boys and overweight and obesity were more among girls | Rural communities  Statically sig difference in eating habit between gender e.g. males more likely to skip meals  Eating habits associated with nutritional status –overweight/obese stat sig higher intake of mid-day snacks, eating from street shops, thin adolescents have low intake of protein rich foods/milk products, higher proportion skipping meals, eating cereal for three main meals |
| 1. George et al (2018) | India | Cross sectional survey | School going adolescent 10-19 years (n=449) | BAZ (WHO): overweight (>1SD); obesity (SD>2SD) | Nutritional status | Thinness (6-23%), overweight (28-40%), obese (11-14%) | Rural primarily farming communities |
| 1. Greene-Cramer (2016) | India | Cross sectional survey | School going adolescents 10-14 years (n=861) | BMI (WHO): thinness (<5^th^ percentile); overweight (85^th^-95^th^ percentile); obesity (=>95^th^ percentile) | Nutritional status | Thinness (7.35%) & overweight/obese (29.96%) | Private schools  Obese/overweight more prevalent in younger age group  Thinness more prevalent in order age group  Having one parents overweight associated with childhood overweight/obesity |
| 1. Harding et al (2019) | Bangladesh (1997 & 2007 & 2014), India (1999 & 2006 & 2016), Nepal (1996 & 2006 & 2016), Afghanistan (2013), Maldives (2009 & 1996), Pakistan (2013) | National Surveys- Cross-sectional nationally  representative survey | Adolescents girls 15-19 years – Afghanistan (2013 n = 2231); Bangladesh (1997 n=668 & 2007 n=1096 & 2014 n=1667); India (1999 n=5119, 2006 n=21 818, 2016 n=118 602); Maldives (2009 n=75); Nepal (1996 n=336 & 2006 n=2282 & 2016 n=1251) | BMI (IOTF); thinness (<18.5 kg/m2), overweight 1 (=>25 kg/m2), and/or overweight 2 (=>23 kg/m2), obese (=>30 kg/m2) | Nutritional status | Afghanistan 2013: thinness (16.7%), Overweight 1 (25.3%); overweight 2 (10.4%), obese (2%)  Bangladesh 1997: thinness (46.6%), Overweight 1 (2.5%); overweight 2 (0.7%), obese (0.7%)  Bangladesh 2007: thinness (30.8%), Overweight 1 (9.3%); overweight 2 (3.2%), obese (0.1%)  Bangladesh 2014: thinness (26.7%), Overweight 1 (19.2%); overweight 2 (8.2%), obese (1.3%)  India 1999: thinness (38.1%), Overweight 1 (5.5%); overweight 2 (1.6%), obese (0.1%)  India 2006: thinness (39.1%), Overweight 1 (7.5%); overweight 2 (2.7%), obese (0.3%)  India 2016: thinness (35.1%), Overweight 1 (11.2%); overweight 2 (4.9%), obese (0.9%)  Maldives 2009: thinness (23.4%), Overweight 1 (37.5%); overweight 2 (24.5%), obese (2.8%)  Nepal 2006: thinness (18.8%), Overweight 1 (12.7%); overweight 2 (3%), obese (0.1%)  Nepal 2016: thinness (23.8%), Overweight 1 (14.7%); overweight 2 (4.4%), obese (0.4%)  Pakistan 2013: thinness (28.8%), Overweight 1 (12.7%); overweight 2 (3%), obese (0.1%) | National surveys: Afghanistan National Nutrition Survey  and DHS surveys  Overweight more prevalent in urban areas  Overweight and obesity among adolescent girls is inconsistently measured or reported, and that when reported reports often include different age groups and definitions for overweight and obesity  National surveys |
| 1. Hassan et al (2020) | Bangladesh (December 2016-Novemver 2017) | Cross sectional survey | School going adolescents 14-19 years (n=825) | BMI (IOTF); thinness (<18.5 kg/m2); overweight (=>25 kg/m2); obese (=>30 kg/m2)  BAZ (CDC): thinness (5^th^ percentile); overweight (85^th^-95^th^ percentile); obesity (=>95^th^ percentile)  BAZ (WHO): thinness (SD <-2SD); overweight (>=1SD); obesity (>+2SD) | Nutritional status | WHO: thinness (8.48%); overweight (10.67%); obesity (4.96%)  IOTF: thinness (17.75); overweight (10.42%); obesity (1.33%)  CDC: thinness(9.33%); overweight (9.82%); obesity (2.79%) | Boys higher risk being overweight and obese  Thinness more prevalent in girls |
| 1. Hemamalini et al (2016) | India | Cross sectional survey | School going adolescents 11-15 years (n=100) | Recall and weight measurement method of 24hr time period were used to collected for 3 days  BMI: thinness grade 1 (17-18.5 kg/m2), grade II (BMI 16-17 kg/m2): overweight (=>25 kg/m2) | Nutritional status | BMI: Thinness grade 1 (31-37.7%), grade 2 (24.2-30%); overweight (10-10.4%)  Calcium (39.3-37.11%), Vit-A (42.42-43.86%), Vitamin C (15.6-8.89%) deficient intake (RDA) |  |
| 1. Higgins-Steele et al (2016) | Afghanistan (2013) | National Nutrition Survey- Cross-sectional nationally  representative survey | Adolescent girls 10-19 years (n=14 237) | Anaemia (Hb levels < 11.99 gm/dl); thinness (BMI <18.5); severe thinness (BMI <17) | Nutritional status | Anaemia (29.9%); thinness (8%) and severe thinness (1.5%) | Afghanistan National Nutrition Survey  Nutritional status of adolescent girls was assessed for the first time in Afghanistan National nutrition 2013 survey  Ministry of health priority area include targeting adolescent girls through school programs and including weekly IFA supplementation for both in‐school and out‐of‐school adolescent girls |
| 1. Iqbal et al (2020) | Pakistan (May-November 2017) | Cross sectional survey | Child labours 5-14 years (n=634) | Stunting (HAZ <-2SD) and wasting (WHZ <-2SD); food frequency questioner (past 30 days) | Nutritional status | Stunted (15.5%) and wasted (30%)  Majority of the children had an inadequate daily intake of all the food groups (fruits, vegetables, legumes, milk/dairy products and meat/ poultry) | Stunting more prevalent in girls  24% of children stated they had lost weight because of being unable to afford food.  High intake of tea and sweets  Malnutrition was no worse than general population – high variation depending on occupation, healthy children chosen tow work |
| 1. Jayalakshmi et al (2017) | India (June-August 2014) | Cross sectional survey | School going children 6-10 years (n=322) | WHO: Stunting (HAZ <-2SD); underweight (WAZ <-2SD); thinness (BAZ <-2SD); severe stunting (HAZ <-3SD); severe underweight (WAZ <3-SD); severe thinness (BAZ <-3SD)  CIAF Grouping | Nutritional status | Stunting (13.4%); underweight (38.8%); thinness (30.7%)  Severe stunting (0.9%), underweight (9.3%) and thinness (9%)  CIAF: thinness only (6.5%), thinness and underweight (18%); thinness, stunting and underweight (6.2%); stunting and underweight (6.8%), stunting only (0.3%), underweight only (7.8%) | 31.1% had multiple anthropometric failure  Thinness and severe underweight were more prevalent in children who occasionally/never took mid-day meal  Excluded overweight (1.6%) and obese (.4%) participants  Anthropometric failure associated birth order, birthweight, illness in the past two weeks |
| 1. Jayatissa et al (2019) | Sri Lanka (July-November 2017) | Cross sectional survey | School going children 10-18 years (n=2525) | Vit D deficiency (25 (OH)D <12 ng/mL); Vit D insufficient (25(OH)D 12-20 ng/mL)  BAZ (WHO): thinness (<-2SD), overweight (+1SD); obese (>2SD)  Stunting (HAZ <-2SD) | Nutritional status | Stunting (13.4%); thin (27%); overweight (8.5%); obese (2.2%); Vit D deficient (13.2%); Vitamin D insufficient (45.6%)  Daily consumption of food rich in vitamin D was low | Cross-sectional national level study  Vit d associated with stunting and age; associated with obesity, eco-agrological zone and gender (males) |
| 1. Kapoor et al (2018) | Pakistan (January -December 2016) | Cross-sectional survey | School going children 12-17 years (n=300) | WHO: Stunting (HAZ <-2SD); underweight (WAZ <-2SD); thinness (BAZ <-2SD); overweight (BAZ <1SD); obesity (BAZ <2SD) | Nutritional status | Stunting (32-34%); underweight (34-39%); thin (16-34%); overweight 2.6%; no obesity  Anaemia (50.6-70%) | Energy, carb, protein, fat and iron intake below RDA  Girls had higher iron intake, but lower energy, carb and fat intakes |
| 1. Karki et al (2019) | Nepal (May-October 2017) | Cross-sectional survey | School going children 6-13 years (n=575) | BAZ: thinness (5^th^ percentile); overweight (85^th^-95^th^ percentile); obesity (=>95^th^ percentile)  SPANS 2010 questioner | Nutritional status | 1/5 children consumed hot chips and crisp 3-4 times a week.  17% had confectioners and ice-cream more than 5 times a week.  83% took homemade food to school  Half of children engaged in small screen recreation for less than an hr a day.  43.6-50.1% of children performed one physical activity at least once a week  Obesity (7.1%); overweight (18.6%); thinness (11%) | Private schools  Overweight/obesity more prevalent among male (cultural factors)  High junk food consumption stag sig associated with overweight/obesity |
| 1. Kujur et al (2020) | India (November-December 2017) | Cross-sectional survey | School going children 5-14 years (n=962) | BAZ (WHO): thinness (SD <-2 to -3 SD); severe thinness (<-3SD); overweight (>=1SD); obesity (>+2SD)  HAZ (WHO): Stunted (<-2Sd to -3SD); severe stunting (<-3SD)  WAZ (WHO): underweight (<-2Sd to -3SD); severe underweight (<-3SD)  Anaemia (WHO classification) | Nutritional status | Stunted (24.8%); severe stunting (12.8%); underweight (31.6%); severe underweight (12.5%); thinness (24.8%); severe thinness (8.8%); overweight (.2%); obesity (.3%)  Mild anaemia (17.3%); moderate anaemia (59.3%); severe anaemia (3.6%) | Primarily tribal population |
| 1. Kumar et al (2017) | India (April 2014-August 2015) | Cross-sectional survey | School going adolescents 13-15 years (n=2952) | BAZ: thinness (5^th^ percentile); overweight (85^th^-95^th^ percentile); obesity (=>95^th^ percentile) | Nutritional status | Thinness (22.12%); overweight (1.15%); obese (.6%)  Junk food consumption (3.2%); did not eat fruit (35.6%) or veg (5.2%) in the past 7 days | Government schools |
| 1. Manandhar et al (2020) | Nepal (2019) | Descriptive cross-sectional study | School going children 6-17 years (n=356) | BAZ: thinness (5^th^ percentile); overweight/obese (=>85^th^ percentile); obesity (=>95^th^ percentile) | Nutritional status | Thinness (18.3%); overweight or obese (4.5%); obesity only (1.7%) | Private school |
| 1. Manandhar et al (2019) | Nepal (March-April 2018) | Cross-sectional survey | School children (n=440) | BAZ (WHO): overweight (>=1SD); obesity (>+2SD)  24hr recall | Nutritional status | More than half of children consumer unhealthy snacks  Overweight (13.2%) and obesity (6.8%) | 87.3 watch TV - One third children watch TV for <2hr/d  Consumption of sugary snacks(not unhealthy) and distance to green space associated with overweight/obesity |
| 1. Mansoori et al (2018) | Pakistan (2012) | Cross-sectional survey | School going children 6-15 years (n=887) | BAZ (CDC): thinness (5^th^ percentile); overweight (85^th^-95^th^ percentile); obesity (=>95^th^ percentile) | Nutritional status | Overweight (19.1%) and obesity (10.8%) | Public and private schools  (66.4% of overweigh/obese) private schools, (76.2%) watch TV for >2h/day were ass with overweight/obesity  Junk food was not sig |
| 1. Naotunna et al (2017) | Sri Lanka | Cross- sectional survey | School going children 5-10 years | BAZ (WHO): thinness (SD <-2 to -3 SD); severe thinness (<-3SD); overweight (>=1SD); obesity (>+2SD)  Stunting (HAZ <-2SD), server stunting (<-3SD)  Underweight (WAZ <-2SD)  Anaemia (WHO): mild (11.9-11 g/dl), moderate (10.9-8 g/dl) and severe (<8 g/dl) | Nutritional status | Severe thinness (7.5%), thinness (21.2%), overweight (3%) and obesity (2.5%)  Stunting (12.6%) of which 2.4% severe stunting  Underweight (27.1%)  Anaemia (17.1%): one child had serve anaemia, mild (9.4%) and moderate (7.6%) | Rural schools  Sri Lanka net enrolment is 99.8%  Increase in malnutrition (both forms) with age  Males had lower BMI  Anaemia and thinness more prevalent in schools furthest way from cities |
| 1. Pal et al (2017) | India (May 2014-April 2015) | Cross- sectional descriptive survey | Adolescents 10-17 years (n=839) | WHO growth ref: stunting (HAZ<-2SD); Thinness (BMI 5th percentile); overweight-obese (BMI <85^th^ percentile) | Nutritional status | Stunting (53.57%); thinness (48.75%); overweight-obese (4.64%)  & 1% of thin adolescents were also stunted & .69% of overweight-obese adolescent were also stunted | Rural areas – community based  Stunting increased with age  Higher risk of stunting among girls  Thinness was more prevalent in younger adolescents  Father's occupation, mother's education, economic status and sanitation, mother working status, family size etc associated with undernutrition |
| 1. Pandey et al (2018) | Nepal | Cross- sectional descriptive survey | Grade 10 adolescents: 15-16 years (n=120) | WHO growth ref: thinness (BAZ <5^th^ percentile); overweight (BAZ 85^th^ – 95^th^ percentile) and obesity (BAZ >95^th^ percentile) | Nutritional status | Thinness (11%), obesity (3.3%) and overweight (10%) | Private schools  Majority of students had adequate knowledge on obesity – no ass between knowledge and obesity  School main source of information |
| 1. Parry et al (2016) | India, Kashmir (June 2011 and June 2014) | Cross- sectional survey | School going children 5-19 years (n=1131) | BMI (WHO): thinness, (21.5 kg/m2); overweight/obesity (> 25 kg/m2) | Nutritional status | Thinness (74.63-88.8%) and obese/overweight (2.08-5.13%) | BMI higher in females in age group 15-19 years but not in younger age groups |
| 1. Pinni et al (2019) | India | Cross- sectional survey | School going children 5-11 years (n=1022) | BMI (WHO growth charts): thinness (<5^th^ percentile); overweight and obese (=>85^th^ percentile) | Nutritional status | Thinness (40.3%); overweight and obese (3.7%) |  |
| 1. Pramod et al (2019) | India | Cross- sectional survey | School going adolescents 10-19 years (n=227) | BMI: thinness; severe thinness; overweight; obesity | Nutritional status | Severe thinness (7%); thinness (11.9%); overweight (6.6%); obesity (1.8%)  One respondent had daily fruit consumption; daily veg consumption (95.2%); 34.4% consumed junk food more than 3 times/week | Study population: lower-socio economic status  Urban area |
| 1. Roy et al (2016) | India | Cross- sectional survey | School going girls 9-18 years (n=500) | WHO criteria for stunting (HAZ) and thinness (BAZ) | Nutritional status | Stunting (39.6%) and thinness (26%) | Stunting increased with age  Thinness was significantly higher in earlier age groups |
| 1. Saha et al (2018) | Bangladesh (September 2014) | Cross- sectional survey | School going children 10-14 years (n=288) | CDC growth reference | Nutritional status | Overweight (8.68%) and obese (5.55%); thinness (18.05%) | Sex, Tv watching and fast-food intake did not affect prevalence of obesity/overweight.  Obesity/overweight higher among middle/higher socio-economic class |
| 1. Saikia et al (2016) | India | Cross- sectional survey | School going adolescent 10-14 years (n=800) | WHO 2007 growth reference | Nutritional status | Overweight (22.5%) and obesity (9.7%)  61.89% had three meals a day, 5.5-11.6% skipped breakfast, 27.3% and 55.2% had daily intake if green leafy veg and fruit, 29-30% had daily consumption of meat, while approx. 30% had fast food, confectionary and soft drinks daily | More prevalent in girls and younger ages  Less frequency of major meals, extra snacks, irregular food habit, breakfast skipping, and increased restaurant visits were associated with increase in overweight/obesity |
| 1. Satyabodh et al (2017) | India | Cross- sectional survey | School going children 11-16 years (n=246) | Cole et al/IOTF (International) – server (16 kg/m2), moderate (17kg/m2) and mild (18.5kg/m2) thinness, overweight (25 kg/m2)  Anaemia (WHO): mild (11-11.9 g/dl), moderate (8-10.9 g/dl) and serve (<8 g/dl) | Nutritional status | Mildly thin (31.3%); moderately thin (12.9%); severely thin (10.9%); overweight (.7%)  Mild anaemia (14.35); moderate anaemia (3.4%); severe anaemia (1 participant) | Boys had higher haemoglobin levels  All participants had poor physical fitness  Tribal adolescents |
| 1. Throne-Lyman (2019) | Bangladesh (September 2015-March 2017) | Data collected as part of follow-up study of children whose mothers had participated in JiVitA-1 trial (Cluster RCT) | Adolescents 9-14 years (n=30 702) | Food frequency questioner; BAZ using WHO growth reference | Nutritional Status | Thinness (27.7%); overweight (1.9%); obesity (.3%)  “traditional” and “low vegetable and low fish” patterns were the two most prevalent - relatively moderate to low dietary diversity. Rest of the population was categorised as the “least diverse” (20%) “moderately high meat” (20%) and “most diverse” (9%) pattern | Rural areas  Majority of both those in the most diverse and moderately high meat group were boys and those in the least diverse group were girls  Adolescents adhering to either the most diverse or moderately high meat patterns had higher mean HAZ  Highly processed foods, biscuits/cake, and fried foods, a median of about two to three times each per week |
| 1. Twinkle et al (2020) | India (July-August 2018) | Cross-sectional study | School going children 6-14 years (n=208) | WHO growth reference: Stunted (HAZ >-2SD); underweight (WAZ <-2SD); thinness (BMI <-2SD) | Nutritional Status | Stunted (46.34%); underweight (67.8%); thinness (48.29%) | Slum areas |
| 1. Singh et al (2019) | Nepal (October-December 2018) | Cross-sectional study | Adolescent girls 10-19 years (n=407) | Food frequency questioner; DD – Low/medium/high (FAO)  BMI: thinness (<18.5); overweight (25-29.9); obese (>=30) | Nutritional Status | 88.2% had insufficient FV consumption; 83.5% eat junk food 1/week; DD low (14.7%) and medium (63.7%)  thinness (24.3%); overweight/obese (15%) | Community based study (rural areas)  Education level, household income, awareness of recommended FVs intake, availability of FVs, dietary diversity and body mass index were significant determinants of FV consumption |
| 1. Siva et al (2016) | India (April 2013-May 2014) | Cross-sectional study | Adolescent girls 10-19 years (n=270) | Anaemia (WHO cut offs)  BAZ (WHO classification) | Nutritional Status | Thin (17.2%); overweight/obese (12%)  Anaemia (21%); mild (19.1%) and moderate (1.9%) anaemia | Factors associated with anaemia were worm infestation, number of pads per day, washing hands before food intake and foot wear usage. |
| 1. Van Tuijl et al (2020) | Nepal (2014) | Nepal Adolescent Nutrition Survey- Cross-sectional study | Adolescent 10-19 years (n=3773) | Stunting (HAZ <-2SD); thinness (BAZ <-2SD) using WHO ref data | Nutritional Status | Stunting boys (32.7%) and girls (28.1%)  Thinness girls (7.2%) and boys (14%)  Male sex, older age, belonging to a religious minority, paternal occupation in business, daily wage, retirement or other and living in the Mid-Western and Mountain areas were among the main risk factors for stunting.  Male sex, belonging to a religious minority, a low household income, maternal education beyond primary education and paternal foreign employment were the main factors associated with increased odds of thinness | Nepal Adolescent Nutrition Survey 2014  Stunting increased with age, while thinness odds decreased with age  Ass of adol nutritional knowledge with status (spill over effect, food choices)– expect girls thinness (agency)  interventions should not only be aimed at improving adolescent nutrition but also at optimizing adolescents’ environment |
| 1. Goonapienuwala et al (2019) | Sri Lanka (April 2013-November 2014) | Cross-sectional descriptive study | School going adolescent 13-16 years (n=3128) | BAZ (WHO criteria) | Nutritional Status | Thin (21.71%); overweight (7.13%); obese (2.35%)  31% of normal weight adol desired larger body size | Rural area  More girls underestimated while more boys overestimated body size  More boys were dissatisfied with their body size  Overweight students were most dissatisfied with their body size |
| 1. Karki et al (2019) | Nepal (April-July 2016) | Cross-sectional survey | School going children 5-15 years (n=1137) | IOTF and WHO growth reference charts | Nutritional Status | IOTF: thinness (38.6%) and overweight (7.9%)  WHO: Thinness (15.4%) and overweight (4.6%) |  |
| 1. Islam et al (2020) | Bangladesh (January-December 2018) | Cross-sectional survey | School going children 12-18 years (n=854) | BMI (WHO growth ref) | Nutritional Status | Inadqeute intake FV (97.6%)  thinness (7.1 %); overweight (9.8%); obesity (9.8%) | Rural and urban areas  High prevalence of overweight/obesity in urban areas |
| 1. Rai et al (2019) | Nepal (2016) | Nepal DHS- Cross-sectional survey | Adolescent girls 15-19 years | WHO BMI cut offs: thinness (<18.5); overweight/obese (>=25) | Nutritional Status | Thinness (30.4%) and Overweight (3.3%) | DHS 2016  Thinness most prevalent in adolescents |
| 1. Scott et al (2020) | India (2015-16) | UDAYA survey- Cross-sectional survey | Adolescent 10-19 years (n=14,793) | >18 years (IOTF) and <18 years (WHO)  Anaemia (age/sex specific WHO cut offs) | Nutritional Status | Anaemia: boys (27.39%) and girls (58.52%)  Thinness boys (45.99%) and girls (38.93%) | Lives of Adolescents and Young Adults survey in Uttar Pradesh and Bihar (state representative)  Rural, high poverty states in north India |
| 1. Asif et al (2020) | Pakistan (March-June 2016) | Cross-sectional study | School ging children 5-14 years (n=7921) | BAZ (WHO growth refence) | Nutritional Status | Overweight (16%); obesity (3.3%); thinness (1.9%) | Urban areas – 4 major cities of Pakistan |
| 1. Nithya et al (2017) | India (2014) | Cross-sectional study | Adolescent 13-17 years (n=183) | BAZ (WHO criteria); 24hr recall; DD (FAO 13 food groups) | Nutritional status | Thinness males (50.7%) and females (27.3%); stunting in  males (50.7%) and females (27.3%)  Cereal based diet – 64% total energy intake.  Majority had low DD |  |
| 1. Biswas et al (2020) | Bangladesh, Nepal, India and Pakistan (2001-2007) | Systematic Review and Meta-analysis | Girls 15-19 years (9 DHS surveys, from Bangladesh (4) Nepal (3) India (1) and Pakistan (1)) | Underweight: BMI < 18.5 kg/m2; overweight as BMI ≥ 25 kg/m2 | Nutritional Status | Overweight (3.5%) and underweight (30%) | Systematic review included DHS data  Pooled prevalence |
| 1. Rahman et al (2019) | Bangladesh (2004 and 2014) | Demographic Health Survey- Cross sectional survey | Adolescent girls 15-19 years (2004 n=10431 & 2014 n=1619) | Thinness (BMI < 18.5 kg/m2); overweight (BMI ≥ 25 kg/m2); obesity (=>30 kg/m2) | Nutritional Status | 2004: thinness (39.7%); overweight (1.5%); obesity (.3%)  2014: thinness (31%); overweight (5.8%); obesity (1.3%) | Decrease in thinness and increase in overweight/obesity  DHS data |
| 1. Dutta et al (2019) | India (2015- 2016) | National Family Health Survey- Cross sectional survey | Adolescents 15-19 years (n=136 417) | Thinness: BMI < 18.5 kg/m2; overweight/obesity BMI ≥ 25 kg/m2 | Nutritional Status | Thinness: men (44.9%) and women (42%)  Overweight/obesity: men (4.8%) and women (4.3%) | National family Health Survey  Thinness highest among 15-19 year olds for men and women |
| 1. Risk Factor Collaboration (2017) | South Asia (1975-2016) | Secondary data analysis (population-based studies) | Adolescents 5-19 years | BAZ (WHO growth reference): moderate/serve thinness (BAZ >2sd); overweight (>1SD); obese (>2SD) | Nutritional Status | 2016: moderate and serve thinness: girls (20.3%) and boys (28.6%)  South Asia one of the highest increases in the number of obese adolescents – rapidly growing population  South Asia – pop increase has led to an increase in the absolute burden of thinness | Mean BMI lowest in South Asia  Global increase in BMI  South Asia rise in BMI has accelerated since 2000 for both sexes  Higher BMI in girls than boys observed in many South Asian countries  Decreasing trend in thinness  Pooled analysis - population based studies including national/subnational surveys |
| 1. Campisi et al (2020) | Pakistan (January 2019-Febuary 2020) | Cross-sectional study | Adolescents 9-15 years (n=1385) | Stunted (HAZ >-2): BAZ (WHO) overweight/obese (BAZ >2), thinness (BAZ <-5); severe thinness (BAZ <-3) | Nutritional Status | Stunted (27-37.2%); overweight/obese (0.4-12%); thinness (15.6-21.8%); severe thinness (4.9-9.5%); stunting & thinness (10.7-11.6%) | Girls higher prevalence of stunting; boys higher prevalence of thinness/severe thinness  Rural |
| 1. Chakraborty et al (2018) | India | Cross-sectional study | School going adolescents 11-17 years (n=2403) | Dietary recall (n=1556); BMI (IOTF) | Nutritional Status | Overweight (23.3%) and obesity (7%)  Higher intake of energy, carbohydrate, protein and fat, vitamin B12, total folate and dietary fibre among urban participants | Lower prevalence of overweight in rural area & no obesity case |
| 1. Chattopadhyay et al (2019) | India (May 2016-April 2017) | Cross-sectional study | Adolescents 10-19 years (n=6352) | BAZ (WHO) | Nutritional Status | Stunted (33.6%); servery stunted (8.1%); thin (17.1%); servery thin (3.9%) | Higher prevalence of stunting among older age groups but higher prevalence of thinness among younger age groups  Rural India  Malnutrition associated with easy access to water, soap usage, not strengthening health services and *Kishori* group - adolescent girls’ club addressing issues specific to adolescent girls using participatory learning and action cycle methodology. |
| 1. Hassan et al (2017) | Pakistan (August 2013-Febaury 2014) | Cross-sectional analytical study | Adolescent girls 14-19 years (n =140) | BMI: thinness <18.5 and overweight >25 m2 | Nutritional Status | thinness (58%) and overweight (8%); 72% experienced food insecurity with 34% experiencing it every month | Peri-urban areas  Associated with malnutrition: father literacy rate, lived in joint families, rented houses or frequently fell ill |
| 1. Lahiri et al (2019) | India (June-December 2017) | Prospective repeated measures study | School going 14-19 years (n=645) | BAZ (WHO) | Nutritional Status | Obesity (.93-7.44%); obesity (.93-1.86%); thinness (12.56-13.33%);  Baseline: 12.40% went hungry most of the time; 47.60% and 23.88% eating fruits and veg <1 per day, respectively. 46.51 and 53.02% had soft drink and fast food more than three times per week, respectively. | Overall: Older age, females, fast food, soft drink, less fruit and vegetable consumption significantly associated with overweight/obesity  Longitudinal study |
| 1. Krishna et al (2020) | India (January-April 2019) | Cross sectional survey | School going children 10-19 years | Food frequency questioner; BMI | Nutritional Status | Thin (11.5%); severely thin (6.9%); overweight (11.5%); obese (12.2%)  45% consumed three meal per day; adequate fruit/veg intake 41.9% and 46.6%. Unhealth foods consumed by approx. 30%; 16.8% skipped breakfast | More males consumed three meals, consumed soft drinks and snacks.  More females skipped breakfast |
| 1. Li et al (2018) | India (November 2011-October 2012) | India Human Development Survey – Cross sectional design | Children 6-14 years (n=20 315) | BAZ (WHO) | Nutritional Status | Obese (5.22%); overweight (6.28%); thin (13.61%); severely thin (7.68%) | Nationally representative – IHDS-II  Household water treatment increased probability of norm weight/overweight/ obesity and decreases probability of thinness |
| 1. Jeyakumar et al (2017) | India | Cross sectional survey | Adolescent girls 16-18 years (n=565) | Repeated 24hr recall; stunting (HAZ) and thinness (BAZ): <-1 mild, <-2 moderate <-3 severe  Anaemia: milk (11-11.9 g/dl); moderate (8-10.9 g/dl); severe <8g/dl | Nutritional Status | 28% did not consume any breakfast; 17% depending on school meal for breakfast had maximum nutrient intake; 99.6% consumed inadequate breakfast – high energy, protein, iron deficient  Anaemia (56%); mild (11%), moderate (43%) and severe (2%)  Stunting: Mild (43.5%); moderate (28%) and serve (5%)  Thinness: mild (25.7%); moderate (7.1%) and serve (1.2%) | Slum areas  Dietary deficits arising in breakfast was not compensated in the subsequent meals |
| 1. Mia et al (2018) | Bangladesh (2014) | Demographic Health Survey- Cross sectional design | Adolescent girls 15-19 years (N=5064) | Thinness (BMI <18.5 kg/m2); overweight BMI (>25kg/m2) | Nutritional Status | Thinness (32.6%); overweight (8.1%) | DHS  Prevalence of thinness in women of reproductive age was highest in girls 15-19 years; obesity was slowest in this age group |
| 1. Sethi et al (2019) | India (2015-16) | National Family Health Survey- Cross sectional design | Adolescent girls 15-19 years (n=9072) | BAZ (WHO criteria) | Nutritional Status | 2015-16: Thin (7.1%); severely thin (1.1%); obesity/overweight (5.3%) | Nulliparous married women  NFHS/DHS data  Proportion of thin adolescent marginally declined (7.8 to 7.1%) and severely thin remined unchanged but overweight/obesity increased (2.3 to 5.3%) between 2005-6 and 2015-16.  Lower prevalence of thinness/obesity/obesity when using  BAZ rather than BMI cut offs  Age, ethnicity, education and poverty influenced BMI |
| 1. Rauf et al (2018) | Pakistan (2011-15) | Longitudinal study | Children 5-17 years (n=1708) | BMI: Thinness (<18.5kg/m2); overweigh (<25kg/m2); obesity (>=30 kg/m2) | Nutritional Status | Thinness (53%); overweight (16%) | Orphaned children |
| 1. Shinsugi et al (2019) | Sri Lanka | Cross-sectional study | School going children (n=543) | BAZ (WHO) | Nutritional Status | Thinness (19.3%); overweight (13.4%); obesity (5.3%) | Maternal employment – thinness more prevalent for housewives  Thinness: maternal educational status, maternal employment status, and maternal nutritional status  Overweight/obesity: maternal employment, household income |
| 1. Kumar et al (2017) | India | Cross-sectional study | School going children 11-14 years (n=1092) | BAZ (IAP growth charts) | Nutritional Status | Thinness (13.1%); overweight (9.6%); obese (3.7%) |  |
| 1. Eroglu et al (2018) | Nepal | Data collected from antenatal micronutrient supplementation trial (Randomised Controlled Trial) | Children 6-8 years (n=3305) | Underweight (WAZ -<2SD); Thin (BAZ-<2SD); stunted (HAZ-<2SD); VAD (retinal <.7 umol/l) | Nutritional Status | Underweight (48.5%); thin (39.1%); stunted (16.1%); Vitamin A deficiency (8.8%) | Nested in supplementation trial for mothers |
| 1. Khan et al (2019) | Bangladesh (2012-13) | Cross-sectional study | School going adolescent 12-17 years (n=793) | BAZ (WHO) | Nutritional Status | Thinness (9%); overweight (17%); obese (7%)  59% reported having breakfast every day | Urban  participants who missed breakfast had a 2.6 times higher odds of being obese than those who regularly ate breakfast |
| 1. Gamage et al (2018) | Sri Lanka | Cross-sectional descriptive study | School going adolescents 17-19 years (n=634) | BMI (WHO ref values for adolescents) | Nutritional Status | Thinness (49.8%); overweight (6.6%); obesity (4.6%)  Only 24.1% (*n* = 153) reported consuming fruits for ≥5 days, and 31.7% (*n* = 201) reported ≥5 servings of vegetables/day | More females with thinness; males higher prevalence of overweight/obesity  Health diet associated with female sex |
| 1. Raqib et al (2017) | Bangladesh | Data collected as part of Randomised Controlled Trial | Children 8-9 years (n=525) | Stunting (HAZ); thinness (BAZ - WHO) | Nutritional Status | Stunting: boys (19%) and girls (23%)  Thinness: boys (40%) and girls (39%) | Rural areas  Nested in MiniMat trial |
| 1. Moonajilin et al (2020) | Bangladesh (November 2019-Febuary 2020) | Cross-sectional study | School going adolescent 13-18 years (n=622) | BMI (CDC cut offs) | Nutritional Status | Thinness (12.2%); overweight (8%); obesity (5%) | Urban area only |
| 1. Rousham et al (2016) | Bangladesh | Secondary data analysis (Save the Children School Health programme) | School going children 6.5-13.5 years (n=900) | BAZ (WHO); stunting (HAZ) | Nutritional Status | Thinness (40.6-44.3%); stunting (34.6-36.8%) | No evidence of gender inequalities |
| 1. Sultana et al (2018) | Bangladesh (June 2012-May 2013) | Cross-sectional study | School going children 6-12 years (n=1768) | BMI (CDC cut offs) | Nutritional Status | Obesity (5%); overweight (10.1%); thinness (14.7%)  67.3% ate junk food daily | Urban areas only  Public and private schools  Higher prevalence of overweight/obesity in high socio-economic status  Overweight/obesity associated with family history of overweight/obesity |
| 1. Schott et al (2019) | India (2001-2016) | Secondary data (Young Lives study) analysis | Children 12-19 years (n= appox.2000) | Stunting (HAZ); Overweight (BMI -IOTF cut offs) | Nutritional Status | Stunting (27.9-36%); overweight (0.8-5.7%) | Young lives cohort  Evidence of catch up growth  Stunting decline, while overweight increase with age  Children who are stunted, more likely to be overweight at follow up  Associated with stunting: maternal education, wealth, rural residence.  Associated with overweight: urban residence, wealth quintiles |
| 1. Banik et al (2020) | Bangladesh (February 2019-June 2019) | Cross-sectional study | School going students 10-19 years (n=518) | BMI (CDC); Food frequency questioner | Nutritional Status | Thinness (11%); overweight (25.3%); obesity (23.4%)  68.3% consumed junk food in the past week; taste and convenience were main reasons for consumption | Urban areas only  Males higher prevalence of obesity  Fast food consumers were 4 times more likely to be obese |
| 1. Saeedullah et al. (2021) | Pakistan (March to April 2020) | Cross-sectional survey | adolescents, aged 10–19 years (n=206) | Thinness, Stunting, overweight and obesity, Vitamin D, Vitamin B12, folate, anaemia, | Nutritional status | Stunting (35.3%), thinness (4.4%), overweight (11.8%) and obesity (3%), vitamin D deficiency (80.5%), vitamin B12 (41.9%), folate (28.2%) and anaemia (10.1%). | Stunting was more common in younger adolescents (10–14 years of age), overweight/obesity two times more prevalent in boys and thinness more among boys. |
| 1. Baxter et al. (2021) | Pakistan (June 2017 to July 2018) | Cross-sectional design | Late adolescent and young women 15–23 years (n=3461) | WHO adult BMI cut-offs for Underweight, Overweight and obesity, Anaemia; iron deficiency anaemia, Vitamin A deficiency, Vitamin D deficiency | Nutritional status | Underweight (42%), Overweight (5.9%), Obese (1%).  Overall Anaemia (47.6%), mild anemia (21.2%), Mod anemia (21.7%), severe (4.7%); Iron deficiency anaemia (38.0%), vitamin A deficiency (31.8%), and vitamin D deficiency (81.1%) | 91% of the late adolescent girls experienced at least one of IDA, VAD, or VDD. |
| 1. Khan et al. (2022) | Pakistan (systematic review, 2000- 2021) | Systematic review | School going children and adolescent (5-15 years) (n=62 148) | Underweight (weight for age z score <-2SD), stunting (height for age z score<-2SD), wasting (weight for height z score <-2SD, thinness (BMI for age z score <-2SD), overweight (BMI for age z score >+1SD), and obesity (BMI for age z score >+2SD), Dietary intake | Nutritional status and dietary intake pattern | Underweight (25.1%), stunting (23%), wasting (24%), thinness (12.5%), overweight (11.4%), obesity (6.9%)  A relatively high intake of carbohydrates, soft drinks, and sweets/chocolates; and a low intake of protein-rich foods, fruits, and vegetables, compared to the recommended daily allowance (RDA) | Underweight, stunting, and wasting was found to be higher amongst females, children from government schools, belonged to low SES, from rural areas and the province of Punjab and Sindh and from disaster-stricken areas.  Thinness was higher amongst males, those attending government schools,  and those from a low SES.  Overweight and obesity was higher amongst males, children going to private Schools, those from a high SES and living in urban areas. |
| 1. Wangaskar et al. (2021) | India (September- October 2019 | Cross-sectional survey | Adolescents (10-18 years) (n=499) | Stunting (height for age z score<-2SD), and severe stunting (<-3SD), thinness (BMI for age z score <-2SD), and severe thinness (<-3SD), overweight (BMI for age z score >+1SD), and obesity (BMI for age z score >+2SD), Undernutrition (either stunting or thinness or both), malnutrition (Undernutrition and/or, overweight and/or obesity), | Nutritional status | Undernutrition (33.3%), Stunting or severe stunting (21.6%), severe stunting (3.4%), Thinness (15%), overweight (10.2%), obesity (5.8%) | Males had 1.4 (1.0–1.9) times more risk of having under-nutrition as compared to females and was found to be statistically significant. |
| 1. Pandurangi et al (2022) | India (CNNS data) | Cross-sectional survey | Adolescents (10-19 years) (n= 31 941 for BAZar and n= 32 045 for HAZ) | Thinness (BAZ<-2SD), Stunting (HAZ <-2SD), overweight (BAZ >+1SD), Obesity (BAZ >+2SD) | Nutritional status | Stunting (27.4%), thinness (24.4%), overweight (4.8%), obesity (1.1%)  Double burden of undernutrition  Stunting and thinness (8.6%)  Stunting and overweight (0.8%) | Stunting predictors- Adolescents in the age group of 15–19 years, females, Muslim, scheduled caste, scheduled tribes, adolescents never attending the school, and belonging to the lowest wealth index  Thinness- Adolescents from lowest wealth index families and OBC were at increased odds, while 15–19 years compared to 10–14 years, female gender compared to males were at decreased odds of thinness.  Adolescents of 10–14 years, urban residents, general category of social class, mother’s education >12 years, highest wealth index families, were at increased odds of overweight. |
| 1. Querol et al (2021) | 8 South Asian countries (Global School-based Student Health Survey)  Pakistan 2009, Afghanistan 2014, Bangladesh 2014, India 2007, Maldives 2009, Nepal 2015,  Sri Lanka 2016, and Bhutan 2016. | Global School-Based Student Health Survey- Cross-sectional survey | School going adolescents (12-15 years) (n=24 053) | Stunting (HAZ <-2SD), Thinness (BAZ <-2SD), Overweight/obesity (BAZ <+1SD) | Nutritional status | South Asia- stunting (13%), Thinness (10.8%), overweight/obesity (10.8%)  Boys vs girls- stunting (11.6 vs 14.8%) thinness (11.8 vs 9.3%), overweight/ obese (11.4 vs 9.9%)  Pakistan- Stunting (3.9%), Thinness (9.09%), Overweight (7.98%)  Afghanistan- Stunting (28.15%), Thinness (2.51%), Overweight (19.04%)  Bangladesh- Stunting (13.52%), Thinness (11.43%), Overweight (11.3%)  India- Stunting (9.65%), Thinness (12.82%), Overweight (12.96%)  Maldives- Stunting (13.33%), Thinness (17.86%), Overweight (13.77%)  Nepal- Stunting (24.09%), Thinness (8.25%), Overweight (9.53%)  Sri Lanka- Stunting (24.99%), Thinness (18.56%), Overweight (13.22%)  Bhutan- Stunting (7.58%), Thinness (1.49%), Overweight (16.34%) | The factors associated with adolescent malnutrition were: age, hygiene behaviours, social support, sedentary behaviour, and tobacco use.  Age 13-15 years were two times likely stunted than students aged 12 years (*p* < 0.001). |
| 1. KC D et al (2021) | Nepal (Dec 2019-May 2020) | Cross-sectional survey | Early adolescents (10-14 y) (n=384) | Thinness (BAZ <-2SD->= -3SD), severe thinness (<-3SD), Stunting (HAZ<-2SD->= -3SD), Severe stunting (<-3SD), Underweight (WAZ <-2SD->= -3SD only for 10y), Severe underweight (WAZ <-3SD) | Nutritional status | Thinness (5.5%), severe thinness (2.6%), stunting (8.4%), severe stunting (5.8%), underweight based on WAZ (13%), severely underweight (1.3%) | Males (9.1%), and 10–12 y (9.1%) were thinner than females (7.1%) and 13–14 y (6.1%).  Female were more stunted (16.0%) and 13–14 y (16.1%) than male (12.6%) and 10–12 y (12.8%) respectively. |
| 1. Saha et al (2021) | India, Gujarat (February to March 2020) | Cross-sectional survey | Adolescent girls n=1252 | Underweight (BAZ <-2SD)  overweight (BAZ >+1SD), Obesity (BAZ >+2SD) | Nutritional status | Underweight (19.6%), overweight (8.9%), obese (2.6%) | A statistically significant association of age (p = 0.00), the number of family members (p = 0.016), knowledge (p = 0.05), and use of toilet (0.041) with low-BMI.  BMI less among the participants aged between 10 and 14 years (144.18, 28.4, 13.7 kg/m2, respectively) than 15 to 19 years (149.19, 40.34, 14.9 kg/m2).  Participants between the ages of 10 and 14 years more underweight (64.62%) than 15 to 19 years (35.84%). |
| 1. Bellundagi et al (2021) | India, Bangalore (December 2016 to May 2017) |  | Adolescent girls (10-19 years) (n=296) | BMI for Asian Indians-  underweight (<18.5 kg/m2), normal weight (18.5–22.9 kg/m2), overweight (23–25 kg/m2) and obese (>25 kg/m2) | Nutritional status | 31% underweight, overweight (27%) and obese (15%).  Urban vs rural difference  Underweight- Urban (21.3%), rural (31.7%), transition (40%)  Overweight – Urban (33.3%), rural (24.4%), transition (21.5%) | Consumption of vegetables, wealth index and per capita income had a positive had a significant influence on the nutritional status. |
| 1. Rao et al (2021) | India, South Andaman | Cross-sectional study | School-going Children 6-8y (n=200) | WHO 2007 reference standard- stunting, wasting, underweight, thinness (BMI<=17kg/m2, Cole et al. 2007) | Nutritional status | Stunting (9.5%), wasting (3%), underweight (10%), and thinness (38%) | Underweight cases higher among boys (14) compared to girls (6) across age.  Thinness higher in both boys (35%) and girls (41%)  For girls, increasing age (6 to 8 years) frequency of thinness was decreasing (66.6%, 35.4%, and 25.6%) but for boys, slightly decrease from 6 to 7 years (42.8% - 21.8%) and then increasing (8 years: 39.3%) overall thinness |
| 1. Kamruzzaman et al (2021) | Bangladesh (northern) (April 2019 to September 2019) | Cross-sectional study | Children and adolescents 2-15 years (n=330) | WHO 2007 reference standard- stunting, wasting, underweight, BMI for age | Nutritional status | 5 to 10 years- stunting (15%), Severe stunting (7), wasting (9%), severe wasting (22%) underweight (16%), severe underweight (14%), overweight (9%), obesity (9%), thinness with BAZ (14%), severe thinness with BAZ (16%), Overweight (6%) and Obesity with BAZ (3%)  10 to 15 years- stunting (13%), Severe stunting (9%), Thinness with BAZ (4%), severe thinness with BAZ (0%), overweight with BAZ (4%), Obesity with BAZ (0%) | Wasting were two times more likely among girls than boys.  Children whose mothers were service holders and low family income quartile were less likely to be wasted than their counterparts.  Stunting was more likely among children from lower family income quartile.  Obese was less likely among low family income quartile than the higher income quartile. |
| 1. Seth U (2021) | India, Delhi (2015) | Cross- sectional study | School going adolescents 12-14y (n=491) | 24-hours dietary recall for 3 consecutive days; WHO 2007 reference standard BAZ classification | Nutritional status | 85% regularly snack such chips, biscuits and kurkure. Almost all children liked package food.  74% (n=364) consumed 3 meals/day, 11% (n=52) consumed 4 meals/day and remaining 15% (n=75) consumed 2 meals/day  The average percent adequacy intake of cereals was 63.5%, pulses 71%, vegetables 54%, fruits 60%, milk and milk products 69%, sugar 187% and visible fat/oils 196%.  The average percent adequacy intake of energy was 87%, protein 74%, iron 69.5%, calcium 59% and vitamin A 80%. The average percent energy intake from fat is 35%, which is high.  Overweight (17%), underweight (42%) and normal (41%) |  |
| 1. Panda et al (2021) | India (DLHS-4 2012-13 and AHS 2014) | District Level Household and Facility Survey and Annual Health Survey- Cross sectional study | Adolescents 10-19 years (n=470 801) | WHO 2007 reference standard BAZ classification-  Thinness BAZ <-2SD; Overweight BAZ>+1SD | Nutritional status | Thinness (24.8%), Overweight (9.4%) and obesity (0.4%)  Thinness boys (27.9%) vs girls (21.8 %)  Overweight boys (8.2%) vs girls (6.8%) | 10 to 14 years and boys more likely to be thin as well as overweight than older adolescents and girls.  Adolescents from lower socio-economic groups and rural areas were at high risk of being thin, while those from high socioeconomic groups and residing in urban areas had a high risk of being overweight. |
| 1. Seema S et al (2021) | India, Haryana (May 2016 to May 2018) | Cross sectional study | Adolescents 12-16 years (n=385) | WHO 2007 reference standard BAZ classification-  Overweight BAZ>+1SD; Obesity (BAZ >+2SD) | Nutritional status | Overweight (17.1%); Obese (6.8%); underweight (22.3%) | Adolescents who were 16 years of age, male gender,  belongs to class III socio-economic status, having nuclear family, non‑ vegetarian, eat junk food regularly and watch TV while eating, 2 hr of screen time, physically inactive, were likely overweight or obese. |
| 1. Tripathi et al (2022) | India, Allahabad (April 2019  and July to September 2019) | Cross sectional study | School going adolescent girls 15-18 years (n=400) | WHO 2007 reference standard BAZ- Underweight; overweight | Nutritional status | Underweight (29.8%), Overweight (4.2%) | Rural vs urban- Underweight (22% vs 37.5%); Overweight (4.5% vs 4%) |
| 1. Bhattacharyya et al (2021) | India, Kolkata (November 2017 to May 2018) | Cross sectional study | School going children 6-19 years (n=508) | WHO 2007 reference standard- Stunting; Severe stunting; Thinness (BAZ <-2SD); overweight (BAZ >+1SD); obesity (BAZ score >+2SD) | Nutritional status | Overall stunting (10.6%), Severe stunting (1.2%), mild to mod. stunting (9.4%), thinness (23.6%) and Overweight (11%) | Stunting- Male had higher odds of stunting in comparison to female. A significant positive association of stunting and age was observed. Children from mothers with educational attainment below primary level higher odds of stunting than children from mothers with minimum secondary education.  Thinness- Higher age (15-19 y), children of mother who had below primary and primary level of education had higher odds thinness compared to lower age (6-9y) and mother having secondary education.  Overweight- Higher educational attainment by parents and higher risk of overweight children was  observed. |
| 1. Verma et al (2021) | India, Punjab (RBSK March and August 2018) | Secondary data analysis-School health surveys | School going children and adolescents 6-18 years (n=897) | WHO 2007 reference standard-Underweight (WAZ); Stunting (HAZ); severe stunting; Thinness (BAZ <-2SD); Severe thinness (BAZ<-3SD); overweight (BAZ >+1SD); obesity (BAZ >+2SD) | Nutritional status | In 5–9 years age group- severely underweight (58.8%), mild to mod. Underweight (25.6%), severely stunted (37.4%), mild to mod. stunted (34.4%) and severely thin (31.8%), mild to mod. thin (23.3%), overweight (0.3%)  In 10–18 years age group- Severe stunting (19.4%), mild to mod. stunting (33.9%), severely thin (26.9%), mild to mod. thinness (24.9%), overweight (0.2%) | A significantly higher  proportion of females aged 5-9 years were severely stunted and thin compared to males. But in middle and late adolescence (10-18 y), more of males were severely stunted and thin compared to females.  No significant gender differences in underweight for 5-9 years.  Higher stunting in early adolescence (10–14 years) as compared to late adolescence (15–18 years). |
| 1. Tariqujjaman et al (2022) | Bangladesh, Dhaka (January to June 2018) | Cross sectional study | School going children 6-15y (n=2690) | WHO 2007 reference standard (WAZ and BAZ)- Underweight, overweight, obesity | Nutritional status | Overall prevalence- Underweight (16%), overweight (19%), obesity (9%) | Children from high-tuition schools, parental NCDs and overweight, and lack of physical activity inside schools were positively associated with overweight and obesity.  Parental NCDs and overweight were negatively associated with underweight. |
| 1. Singh B et al (2022) | India, Pune (January–February 2019) | Cross sectional study | School children 6 to 12 years (n=134) | WHO 2007 reference standard (HAZ, WAZ, BAZ)- Mild to moderate stunting, severe stunting, Mild to mod. underweight, severe underweight | Nutritional status | Mild to mod. stunting (16.4%), severe stunting (3%), mild to mod underweight (36.4%), severe underweight (2.3%).  Anemic (64.18%); out of which mildly anemic (38.37%) and moderate anemia (61.63%) |  |
| 1. Radhakrishnan et al (2021) | India (RSOC-2013-14) | Cross sectional study | Adolescent girls 10-18 y (n=28 000) | WHO BMI classification for underweight, overweight and obese | Nutritional status | Underweight (62.5%), overweight (2.2%), obese (1.3%) | Underweight in early and middle adolescent girls age 10-14y (62.5%) was higher than older adolescent girls age 15-18y (44.7%). |
| 1. Zareen S (2021) | Pakistan, Punjab | Cross sectional study | School going children and adolescents 6-19y (n=384) | WHO 2007 reference standard (HAZ, WAZ, BAZ)- stunting, underweight, anemia | Nutritional status | Underweight (21.1%), Stunting (33.3%), anemia (1.8%) | Girls vs boys- Stunting (42.6% vs 31.3%), underweight (16.2% vs 22.2%) |
| 1. Dhobi S and Giri M (2021) | Nepal (January  2020 to April 2020) | Cross sectional study | School going adolescents 10-19 y (n=242) | WHO 2007 reference standard (BAZ)- underweight, overweight  Food frequency during last 24 hours based on 7 food groups | Nutritional status | Underweight (37.6%), overweight (2.5%)  Malnourished (underweight and overweight) (40.1%)  Food groups consumption-  Grains (100%), Milk and milk products (69.8%), Vitamin A rich fruits and veg (93.8%), Other fruits and veg (82.2%), meat, egg and fish (29.3%), legumes and nuts (38%), oils and fats (97.1%) | Significant association of underweight with gender 64.8% males and 35.2% females  For overweight, both sexes were equal at 50.0% |
| 1. Sathiadas G et al (2021) | Sri Lanka (November  2015 to August 2016) | Cross sectional study | School going children and adolescents 6-16y (n=1012) | WHO 2007 reference standard (HAZ, BAZ)- stunting, thinness, overweight, obesity | Nutritional status | Stunting (11.4%), Thinness (29.8%), Overweight (11.1%), Obesity (6.3%)  Boys vs Girls- stunting (10.9 vs 11.8%), thinness (30.6 vs 29.1%), Overweight (11.3 vs 10.7%), Obesity (8.7 vs 4.1%) | Obesity significantly higher in boys than girls (4.1%) (p < 0.001).  Maternal Education and Family income had a significant impact on the prevalence of stunting, thinness, overweight and obesity.  Family size had a significant impact on the thinness, overweight and obesity.  The residential area had a significant impact on the stunting. |
| 1. Hombaiah et al (2021) | India, Karnataka (September 2018 and March 2019) | Cross sectional study | School going children and adolescents 6-17 years (n=8688) | WHO 2007 reference standard (BAZ)- thinness, severe thinness, overweight, obesity | Nutritional status | Severely thin (14.7%), thin (15.8%), overweight (6.9%), and obese (2.2%) | Boys were more severely thin (18%) than girls (11.4%) and girls (7.6%) were more overweight than boys (6.3%).  More of the rural students (16.2%) were severely thin and more urban students were overweight (8%) and obese (2.7%), respectively.  Severe thinness was maximum in the age group of 6–8 years (40.2%) which has a decreasing trend as age increased (15–17yrs – _12.1%). |
| 1. Ghosh et al (2021) | Bangladesh (July and December 2019) | Cross sectional study | Adolescent girls 12-16 years (n= 422) | WHO 2007 reference standard (HAZ, BAZ)- Thinness, Overweight, Obesity, Stunting  Individual Dietary Diversity Score (FAO, 2011)- low (score 0-3), medium (score 4-5) and high (score 6-9) | Nutritional status | Underweight (9.5%), overweight (13.2%), and obesity (4.3%), Stunting (12.1%)  High IDD score (31.5%), Low IDDS (25.6%) and medium IDDS (42.9%) | Stunting was highest among 15 years old.  HAZ was associated with the age of adolescent’s (p=.037).  Mother’s (p=.005) education was correlated with the BAZ of adolescent girls. |
| 1. Goonapienuwala et al (2022) | Sri Lanka (April 2013 to November 2014) | Cross sectional descriptive study | School going adolescents 13 to 16 years (n=3105) | WHO 2007 reference standard- Thinness (BAZ <-2SD); severe thinness (BAZ -3SD-<-2SD); stunting (HAZ -3SD-<-2SD); severe stunting (HAZ<-3SD); overweight (BAZ +1SD - +2SD) and obese > +2SD | Nutritional status | Obese (2.4%), Overweight (7.1%), thin (15.9%), Severely thin (5.7%), stunted (11%), severely stunted (1.7%) | More boys (3.1%) than girls (1.7%) were obese as well as thin (29.0% compared to 15.0%).  Prevalence of overweight/ obesity was higher among students in urban schools, belonging to high social class and more educated parents. |
| 1. Anwaar et al (2022) | Pakistan, Lahore | Cross-sectional descriptive study | School going children 7-15 years (n=205) | CDC growth reference; 24 hours dietary recall; food frequency questionnaire, | Nutritional status | Underweight (31.2%), overweight (4.9%) and obese (4.4%)  Breakfast, lunch, and dinner were consumed by 75.6%, 81%, and 85.9% respectively.  Daily consumption- chapati (85%), Intake of refined carbohydrates from noodles (8.3%), biscuits (38%) and rusk (6.3%), egg (16.1%), mutton/beef (only 1%), milk (37.1%), yogurt (16.1%), fresh fruits (24.6%), cooked vegetables (9.3%).  1/3 population from our sample consumed empty calories in the form of gums and candies. Daily consumption of candies/chocolates (40%), tea (33.2) and 20.5% consumed carbonated drinks 1 time/week. Fast food consumption rate is high in children, consume shawarma, fries and pizza on weekly basis. |  |
| 1. Pant GP and Paudel P (2021) | Nepal (May to June 2019) | Descriptive cross-sectional study | Grade 4, 5, 6 and 7 school students (n=301) | CDC growth reference for BMI for age)- underweight, overweight, obese | Nutritional status | Underweight (16.3%), overweight (6%), obese (0.7%)  Boys vs girls underweight (19.0% vs 13.8%) and overweight (5.6% vs 6.3%) |  |
| 1. Singh S et al (2021) | India, HP | Cross-sectional survey | Adolescent girls age 13-18y (n=276) | CDC growth reference for stunting | Nutritional status | Stunting (17.4%) | 16-18 years showed significantly greater odds for being stunted than 13-15y.  Adolescent girls belonging  to joint families, lower SES, physically inactive, wasted and thin were at higher odds of being stunted than their counterparts. |
| 1. Khanam et al (2021) | Bangladesh | Cross-sectional survey | School children age 5-10y (n=400) | WHO 2007 reference standard- stunting (HAZ), underweight (WAZ), wasting (WHZ) | Nutritional status | Wasting (48.0%), underweight (40.5%), stunting (38.0%) | Children aged 9–10 years were more likely to be stunted than 5–6 years. 7–8 years and 9–10 years were more likely to be wasted and underweight than 5–6 years.  Children of parents with secondary level education were significantly higher chances of stunting.  Children of mothers with 2 or more children were more 8.5 times and 6.3 times higher likely of being stunted and wasted than mothers with one child.  Children whose families had only agricultural land were more likely to be stunted. |
| 1. Kumar P et al (2021) | India, UP & Bihar (UDAYA survey, 2016) | Understanding the Lives of Adolescents and Young Adults(UDAYA) project survey- Cross-sectional design | Adolescents aged 10-19 years (n=20 594) | WHO 2007 reference standard for thinness (BAZ), stunting (HAZ) | Nutritional status | Thinness (36.6%), Stunting (36.8%), both (34.7%), coexistence of both thinness and stunting (girls 6% and boys 10%)  Boys vs girls: Thinness (25.8% vs 13.1%), stunting (25.6% vs 39.3%), both (9.7% vs 6%) | The odds of stunting and thinness were higher among late adolescents, uneducated adolescents, and poorest adolescents. Thinness was significantly more likely among not working adolescent boys respectively than working counterparts. |
| 1. Jan et al (2021) | Pakistan | Cross-sectional study | School going children aged 5-10 y (n=235) | WHO 2007 reference standard (WHZ, HAZ, WAZ) for underweight, stunting and wasting | Nutritional status | Underweight (16.8%), stunting (27.7%), wasted (9.8%)  Overall undernutrition (35.1%)  Boys vs girls: underweight (7.7% vs 8.9%), stunting (10.2% vs 17.4%), wasted (4.7% vs 5.1%) | Both stunting and underweight were more common in girls than boys. |
| 1. Kumari R and Nandita K (2021) | India | Cross-sectional study | Adolescents aged 6-12 years (n=200) | WHO 2007 reference standard (BAZ, HAZ, WAZ) for underweight, stunting Thinness, overweight, obesity | Nutritional status | Mild underweight (27.5%), Moderate underweight (18%), severe underweight (6%)  Mild stunting (32%), mod stunting (6.5%), severe stunting (0)  Mild-mod thinness (50.5%), severe thinness (45.5%), overweight (1.5%), obesity (0.5%) |  |
| 1. Krishna et al. (2020) | India (January-December 2018) | Cross sectional survey | Medical students (<=19years) | Anaemia (Hb 3 gm/dl in males and Hb <12mg/dl in females).  BMI | Nutritional status | Thinness (8.16%); overweight (27.55%); obese 1 (17.35%); obese II (14.29%).  Anaemia (11.22%) – all had mild anaemia | Association between anaemia, diet and BMI |
| 1. Ashtekar et al (2019) | India (November 2017 to February 2018) | Cross-sectional study | School going children 6-16 years (n=1510) | 7-day dietary recall.  Stunting (IAP percentile <3%) | Nutritional status | Tribal students: Animal source foods (%): private school (0-5.3%) and government school (18-19%; staples (42–50%); legumes and pulses (22–25%)  Stunting: girls at 7 (23.4%) and 15 years (13.7%); boys at 7 years (28.9%) and 15 years (13.7%) | Ashram schools for tribal students  Same dietary guidelines but govern schools revived more funds  Statically sig difference in body weight and height between tribal and urban students. |
| 1. Kapil et al (2017) | India (2015-16) | Cross-sectional survey | School going children 6-18 years (n=1222) | Vit D deficient (25(OH)D <20 ng/ml) and insufficient (20-29 ng/ml)  BAZ: thinness (<-2SD), overweight (+1SD); obese (>2SD)  Stunting (HAZ <-2SD) | Nutritional status | Severely thin (18.4-4.2%), thin (27.2-15.7%), overweight (.8-2.1%) and obese (.7-.2%)  Vit D deficient (80.5-81.1%) and insufficient (15.2-18.6%) | Kullu and Kangra districts – high altitude  Girls had higher prevalence of Vit D deficiency |
| 1. Khan et al (2020) | Nepal (August 2017-september 2018) | Cross-sectional survey | School going children 7-15 years (n=181) | Vit D: insufficient Vit D (25(OH)D 29.9-20); deficient (<20 ng/ml)  BAZ: thinness (5^th^ percentile); overweight (85^th^-95^th^ percentile); obesity (=>95^th^ percentile) | Nutritional status | thinness (11.6%); overweight (4.97%); obese (1.66%)  Vit D deficient (44.8%) and insufficient (27.6%) | Public schools  Males stag sig higher Vit D levels |
| 1. Marwaha et al (2019) | India | Pre-post design | School going children (n=468) | IAP growth reference  Vit D deficient: severe (25OHD <5 ng/ml); moderate (25OHD <10 ng/ml); mild (25OHD <20 ng/ml) | Nutritional status | Obesity (11.3%) and overweight (18.8%); sever (8.8%), moderate (42.7%) and mild (48.5%) Vit D deficient | Fee paying schools  Supplementation in Vit D deficient children resulted in decrease in both bone formation (P1NP) and resorption (CTx). |

**Supplementary Table S5**: Summary of identified evidence on nutritional status interventions targeting school-aged children and adolescents in South Asia

| **Author (year)** | **Country (year of data collection)** | **Study design** | **Target population (sample size)** | **Outcome(s) of interest & classifications criteria used** | **Nutritional status or Interventions** | **Key findings** | **Author’s Notes** |
| --- | --- | --- | --- | --- | --- | --- | --- |
| ***Interventions targeting stunting*** | | | | | | | |
| 1. Varkey et al (2020) | India (January-March 2017) | Randomised controlled trial | School going children 6-10 years (n=806) | Moderate (HAZ <-2SD) and severe (HAZ <-3SD) stunting | Intervention: Feeding micronutrient-fortified, high-quality legume protein-based food product (6, 8, 10 or 12g) to moderately stunted children for 1 month | Moderate (17%) and severe stunting (3%)  No significant difference in HAZ or BAZ between groups | 38 serum metabolites were altered significantly |
| ***Interventions targeting overweight and obesity*** | | | | | | | |
| 1. Thakur et al (2016) | India | Cluster randomized trial | School going adolescents, mean age 13 years (n=462) | BMI, waist circumference, triceps thickness, weight, dietary recall and food frequency questionnaire | Intervention: 20-week school based lifestyle intervention package | Decrease in weight, waist circumference and triceps thickness– no impact on BMI | Significant reduction in intake of energy, protein and fat but no to minimal reduction in biochemical parameters |
| 1. Nayak et al (2016) | India (August 2009 and February 2012) | Randomised controlled trial | School going children 14-16 years (n=131) | Lifestyle practices and self-esteem scores; BMI, triceps, biceps, subscapular skin fold thinness | Intervention: target at obese children for 6 months – increased physical activity in school, education on lifestyle modification for children and parents | Non-significant decrease in BMI (p= 0.34); significant decrease in skinfold measurements: triceps (p < 0.001), biceps (p < 0.001), subscapular (p < 0.001). Improved lifestyle practices (p =0.009) |  |
| ***Interventions targeting micronutrient deficiencies*** | | | | | | | |
| 1. Adams et al. (2017) | Bangladesh (September 2011 – November 2017) | Cohort pre-post research design with a control group | School going children 6-11 years from disadvantaged areas (n=351) | Anaemia (Hemoglobulin <11.5g/dL); IDA (ferritin <15.00 ng/mL); Vit A (serum retinol <0.70 umol/L) | Intervention: School-based micronutrient fortification biscuit program – pre-post study design | Baseline: Anaemia (12%); Vitamin A deficiency was slightly higher (36%), iron deficiency anaemia (5.5%).  Endine: Significant positive impact on mean levels of haemoglobin, folic acid, vitamin B12, retinol and vitamin D, and helped reduce anaemia and vitamin D deficiency. | While school feeding programs cannot reverse the damage of early nutritional deficits, evidence suggest school feeding/fortification programs can have measurable impacts on micronutrient deficiencies, school attendance, and the growth |
| 1. Bhuvaneswari et al (2020) | India | True Experimental study (Pre-test post-test with control) | Adolescent 10-19 years (n=170) | Anaemia severity using WHO anaemia severity assessment scale | Intervention: honey dates amla mix | Baseline: Mild anaemia (23.5-38%); Moderate anaemia (59-73%); severe anaemia (4%).  Endline: Alternative herbal supplementation is effective treatment of iron deficiency anaemia among adolescent girls | There is significant reduction of clinical symptoms of iron deficiency anaemia among adolescent girls. |
| 1. Bansal et al (2016) | India | Randomised double-blind clinical trial | Adolescent girls 11-16 years (n=794) | Anaemia: mild (100-119 g/l) and moderate (70-99g/l) and severe (>70g/l) anaemia | Intervention: mild and moderately anaemia gilds weekly supplementation with IFA+ Placebo or IFA+ cyanocabalamin | Anaemia (58.7%): mild (46.5%), moderate (11.9%) and severe (0.25%) anaemia  Supervised IFA supplementation with and without vitamin B12, increased mean haemoglobin levels and reduced the prevalence of anaemia by 37.9% | Slum areas in Delhi  Anaemia prevalence increased with age |
| 1. Kumar & Erhardt et al (2020) | India | Pre–post-test design | School going children 5-17 years (intervention n=128, reference group n=100) | Iron deficient (serum ferritin <15 ug/L)  Iodine deficient (UIE <100 mcg/L) | Intervention: Noon meal programming with multiple micronutrition fortified salt (1 year) | Baseline: Iron deficiency intervention (50%) and reference (34%) group  Endline: sTfR decreased in the intervention but increased in the reference group, while body iron stores increased in the intervention and decreased in the reference group - indicate a decrease in the intervention group. However, no sig changes in serum ferritin and urinary iodine were observed in either group or between groups. | Baseline: children consumed approximately 2.5–3 g of salt per meal = approximately 2.5–3 mg of iron, which hypothesised to be to low to improve iron levels  Intervention: consume 2.5–3 g of fortified salt, they acquire approximately 7.5–9 mg of chelated iron, 225–270 µg of iodine, 7.5–9 mg of zinc, 3–3.6 µg of vitamin B-12, and 75–90 µg of folic acid. 50 % iron, 75-90% of zinc, 100% B12 and 75=90 folic acid RDA. |
| 1. Mandlik et al (2018) | India (August 2014-Febuary 2015) | Randomized double-blind placebo-controlled trial | Children 6-12 years (n=106 supplemented) | Vitamin D deficiency (<50 nmol/L); insufficiency (50.0–74.9 nmol/L) | Intervention: daily supplementation for 6 months of 1000 IU vitamin D & 500 mg calcium (RCT) | Baseline: Vit D deficient (23%) and insufficient (68%)  End line: effective in raising serum 25(OH)D concentrations to optimal levels of ≥75 nmol/L in almost 50% of the children | Lower baseline serum 25(OH)D concentrations change in serum concentrations were higher.  70% of children who were deficient were able to achieve optimal levels compared to 43% of children who were insufficient |
| 1. Rahman et al (2016) | Bangladesh (April 2002) | Double-blind cluster randomised controlled trial | School aged children 6-15 years (n=352) | Low serum retinal (<1.05 umolL-1);; iron deficiency (serum ferritin <20ug/L-1) | Intervention: CRCT -fortified wheat flour for 6 months | Baseline: VAD (13.6-15.4%); low serum retinal (63.6-67%); Anaemia (24.3-30.3%), iron deficiency (19.4-24.5%).  Endline: sig reduction in VAD at 3 and 6 months & low serum retinal at 6 months; No sig effect on anaemia, iron and haemoglobin status | Rural Bangladesh  Still high prevalence of low serum retinal – longer exposure required  amount of iron consumed by the intervening children from fortified flour was 6.6 mg day^–1^ which might not be sufficient to reduce iron deficiency and anaemia as their iron consumption from regular diet might also be low  iron may not have been as bioavailable as originally assumed |
| 1. Kumar et al (2021) | India | Pre-post-test deign | Children and adolescent 5-17 years (n=117 in experimental group and 95 in control group) | Anaemia, zinc deficiency, iodine deficiency | Intervention: multiple micronutrient (iron, iodine, vitamin B12, folic acid and zinc) fortified salt  (For 8 months from April 2012 to beginning of January 2013) | Baseline:  Intervention group- Anaemia (67.5%),  zinc deficiency (32.7%)  Control group- Anaemia (46.3%), Zinc  deficiency (22.1%)  Endline:  Decrease in the prevalence of anaemia  from 67.5% to 29.1%) and zinc  deficiency from 32.7 to 12.4%) in the  experimental group.  No change in urinary iodine in the  experimental group while it decreased in the control group. | Fortified Crystal salt was effective in decreasing multi-micronutrient deficiencies. |
| 1. Kumar et al (2021) | India (October 2017 to January 2019) | Comparative observational study | School going children 5-19 years (n=318, 159 each in intervention and control group) | Status of serum micronutrients (vitamin A, D, B12, calcium) and anaemia | Intervention: 200ml fortified (vitamin A and D) milk supplementation for 1 year | Intervention group- Vitamin D deficiency (99.4%), vitamin B12 deficiency (25.2%), calcium deficiency (8.8%), anaemia (31.4%)  Control group- Vitamin D deficiency (99.4%), vitamin B12 deficiency (46.5%), calcium deficiency (24.5%) and anaemia (41.5%) | The study reported milk  consumption could help in improving the calcium and  vitamin B12 status of school children of a tribal state,  whereas it does not have any significant effect on vitamin  D level. |
| 1. Gupta et al (2022) | Pakistan (November 2019 and March 2021) | Cluster-randomised, double-blind, controlled effectiveness trial | Adolescent girls (10-16 years) (n=517, control=260 and intervention-257) | IZiNCG cut offs for Zinc deficiency- Plasma concentrations of zinc (PZC) levels < 650g/L for girls below 10y and <660g/L for girls 10 years or older  Storage iron deficiency- Serum ferritin (SF) <15 ng/mL  Functional iron deficiency- Serum transferrin receptor (sTFR) > 4.59 mg/L  Iron deficiency- SF <15 ng/mL or sTFR > 4.59 mg/L  Anaemia- WHO Hb criteria  Hb levels were <11.5 g/dL for girls aged <12 years and <12.0 g/dL for those aged >=12 years.  Iron deficiency anaemia- anaemia + Iron deficiency | Intervention: zinc-biofortified flour for 25 weeks  Control: control flour for 25 weeks | Baseline-  Intervention group: Zinc deficiency (71.9%), storage iron deficiency (40.3%), functional iron deficiency (13.6%), iron deficiency (44.4%), Iron deficiency anaemia (8.5%)  Control group: Zinc deficiency (66.1%), iron deficiency (36.2%), storage iron deficiency (30.8%), functional iron deficiency (13.2%), Iron deficiency anaemia (10%)  End line:  Intervention group: Zinc deficiency (64.4%), storage iron deficiency (42.6%), functional iron deficiency (17.1%), iron deficiency (46.8%), Iron deficiency anaemia (13.3%)  Control group: Zinc deficiency (60.3%), storage iron deficiency (41.3%), functional iron deficiency (12.4%), iron deficiency (42.3%), Iron deficiency anaemia (10.8%) | Intervention had no significant effect on PZC, however overall increase in the storage iron deficiency prevalence in the control arm was 11.8% vs. 1.0% in intervention arm. |
| 1. Kunnath AK et al (2022) | India, Meghalaya | Randomised double-blinded, controlled intervention trial | Anaemic Adolescent girls aged 10-19 y (n=123) | Anaemia- WHO Hb criteria | Intervention: Soup made from sodium iron EDTA salt-enriched fish powder (250 mg/100 g) for 60 days  Control- Soup made out of plain fish powder (100 g) with similar composition containing 1.1 mg of Fe | Baseline vs end line-   - Hb levels   Intervention group (n=62)-  Mild anaemia (11.4 vs 13.9g/dl), Moderate anaemia (9.7 vs 11.9), Severely anaemia (7.6 vs 9.1)  Control group (n=61)  Mild anaemia (11.4 vs 11.5g/dl), Moderate anaemia (9.7 vs 9.5), Severely anaemia (7.6 vs 7.7)   - Serum iron   Intervention group (n=62) (6.93 vs 14.85)  Control group (n=61) (7.24 vs 7.85) | The effects of NaFeEDTA salt-enriched fish powder were statistically significant. |
| 1. Shrivastava et al (2019) | India (2014-2016) | Cross-sectional study | School going children 10-19 years (n=768) | Anaemia | Evaluation: Weekly IFA supplementation scheme in schools | Mild (29.68%), moderate (19.27%) and severe (3.13%) anaemia | Rural schools – central India  Coverage (11.54%)  Teachers not knowing difference between deworming, IFA schemes; majority not participated in training; did not know how to access program; Repro health and Child health officer unaware of lack of distribution, had received report of the program running regularly from field staff  Monitoring high burden on PHC Medical officers |
| 1. Scott et al (2018) | India | Randomized Controlled Efficacy Trial | School going adolescents 12-16 years (n=140) | Anaemia (hb <120 g/L for males aged <15 y and all females and <130 g/L for males aged ≥15 y); Iron deficiency (Ferritin <15 ug/L); iron deficient anaemia | Intervention: Iron-fortified pearl millet (6 months) | Baseline: Anaemia (33%), iron deficient (50%), iron deficient anaemia (16%)  Endline: improved iron status and some measures of cognitive performance | Double blind randomized efficacy trail |
| 1. Field et al (2020) | Bangladesh, India and Sri Lanka (2012-2017) | Systematic review | Children 6-15 years (n=5 trials) | Anaemia (WHO criteria) | Intervention: fortification of wheat flour | Iron alone AND Iron + micronutrient vs unfortified: little or no difference to anaemia, probably makes little or no difference to iron deficiency.  Iron + micronutrient vs micronutrients only: inconclusive | Systematic review – 5/9 relevant for this review |
| 1. Resmi et al (2019) | India | Pre and post-test experimental design with control | School going adolescent girls 14-17 years (n=120) | Haemoglobin, serum Vit C and iron levels | Intervention: Amaal Jaggery and pumpkin leaves abstract for 6o days | Significant increase in haemoglobin levels, Vit C and iron levels | Randomised pre-post experimental design |
| 1. Ramirez- Luzuriaga et al (2018) | India (2016) (n=35,197) | Systematic review and meta-analysis from randomized and quasi-randomized controlled trials | Children and adolescents | Anaemia | Intervention: double fortified salt with iron and iodine | Pooled effect size of anaemia were not significant in the two efficacy trails | Systematic review and meta-analysis  Effectiveness trials  4/7 efficiency trails for school aged children were from India (1980-2014): significant impact on hemoglobulin concentrations – study also found significant effect on Anaemia and IDA |
| ***Interventions targeting dietary patterns and dietary quality*** | | | | | | | |
| 1. Thapa et al (2020) | Nepal | Pre-post design | School going adolescent 12-16 years | Dietary patterns | Intervention: 25 hrs (spread over 24 weeks) of continued BAT - experiential learning intervention | Aggregate decline in the consumption of junk food and unhealthy drinks.  Increase in percentage of students consuming healthy food | Self-reported before and after surveys  Positive changes in school adolescents’ self-reported aggregate CVD risk behaviours |
| ***Interventions targeting multiple nutrition status outcomes*** | | | | | | | |
| 1. Devara et al. (2017) | India (September 2015 - March 2017) | Cluster randomised trial | School going children 6 -14 years (n=1600) | Stunting, wasting, Haemoglobin levels, Folic acid, calcium, Thyroid stimulating hormone, Vitamin D, Vitamin A and Vitamin B12 | Intervention: Local vs. Centralized “Annapurna” kitchens | Baseline: thinness (31.9-36.9%); Stunting (30.0-38.2%).  Endline: Intervention and the control groups showed an improvement in percentage of thinness children and stunting as compared to the baseline. | Stunting highest among tribal children (54%)  Provision of regular nutritious meals, through centralized and local kitchen in Ashram schools of Maharashtra, is effective in tackling undernutrition in Tribal children. |
| 1. Anitha et al (2019) | India (July-September 2018) | Parallel group, two-arm, superiority trial | School going adolescent 10-14 years (n=243) | Dietary assessment  Stunting (HAZ <-2SD)  BAZ: undernourished (<-3SD), moderately undernourished (<-2SD), and over-nourished (>2SD) | Intervention: Millet vs rice based mid-day school meals for two months | Baseline: Eat breakfast everyday (56.6%); meals consumed by household members provided 50 to 60% of required calories, 50% of protein, 40 to 50% of iron, 60%  of calcium, and 40–50% of zinc, based on RDA  Endline: Stat significant improvements in stunting and BMI | Cost effective if millets are given government pricing support as equally as rice |
| 1. Marwaha et al (2019) | India (July 2015-December 2017) | Single-blind prospective randomised clinical trial | School going children 6-16 years (n=1008) | IAP growth reference  Vit D (<50 nmol/l); severe (25(OH)D <12·5 nmol/l); moderate (25(OH)D <25 nmol/l); mild (25(OH)D <50 nmol/l) | Intervention: daily supplement of 600 IU or 1000 IU or 2000 IU of D_3_ for 6 months | Baseline: Obesity (8.6%) and overweight (18.7%)  Severe (14.6%), moderate (46.8%) and mild (38.6%) Vit D deficiency  Endline: rise in serum 25(OH)D was maximum with 2000 IU, followed by 1000 IU and 600 IU | Fee paying schools  Two participants developed hypercalciuria, but none developed hypercalcaemia  absence of universal food fortification with vitamin D, supplementation is an effective alternate strategy |
| 1. Shrestha et al (2020) | Nepal (March 2015- June 2016) | Cluster-randomised  controlled trial | School going children 8-17 years (n=682) | Stunting (HAZ <-2SD WHO median); thinness (WHZ <-2SD WHO median); anaemia (Hb <80g/l) | Intervention: CRCT - School Garden with education (SG) or School garden with WASH, health and nutrition interventions (SG+) or control | Baseline: Stunting (17.7-19.9%), thinness (5.7-12.3%); anaemia (20.7-33%)  Endline: SG+ statically sig reduction in intestinal parasitic infections, increased handwashing, fruit and veg consumption and nutrition knowledge. | No effect on stunting and thinness |
| 1. Tamang et al (2019) | Nepal (October 2015 – July 2017) | Cross sectional study | School going children (n=1042, UIC and salt samples n=946) | IDD (WHO criteria) Severe (<20ug/l); mild (20-49 ug/l); moderate (50-99 ug/l); more than adequate (200-299 ug/l); excess (>=300 ug/l) | Intervention: 1 year educational programme | Baseline: Severe IDD (.4%); mild (2.3%); moderate IDD (7.1%); more than adequate (23.5%); excess (34.4%)  End line: slight decrease in prevalence of excessive UIC but increase in IDD | Salt iodisation 0- <15ppm (11.8%)  3.2% of the children were identified to have thyroid dysfunction - hypothyroidism or hyperthyroidism  Statistical significance not reported |
| 1. Muhammad et al (2019) | India (2014-2015) | Baseline and endline survey | School going children 6-18 years (n=1836) | BAZ (WHO) | Intervention: School based health screening and education via counselling, health education by teacher and per-led sessions for 1 year | Baseline: thinness (22.9%) and overweight/obese (6.4%)  Endline: Positive impact on diet and hygiene habits and reduction in thinness | Drawback – lack of parental involvement  Habits most difficult to influence |

**Supplementary Table S6**: Distribution of studies across South Asian countries

| **Country** | **Number of studies** | **Percent distribution** |
| --- | --- | --- |
| India | 174 | 54.0% |
| Pakistan | 40 | 12.4% |
| Bangladesh | 38 | 11.8% |
| Nepal | 34 | 10.6% |
| Sri Lanka | 22 | 6.8% |
| Afghanistan | 6 | 1.9% |
| Maldives | 2 | 0.6% |
| Bhutan | 2 | 0.6% |
| Pooled (South Asia) | 4 | 1.2% |

**Supplementary Table S7**: Internationally recognised definitions for outcomes of interest

| **Undernutrition**  *Underweight*: WAZ <-2 standard deviations (SD) below the World Health Organisation (WHO) Child Growth Reference median  *Wasting*: Weight-for-heigh (WHZ) <-2 SD below the WHO Child Growth Reference median  *Stunted*: Height-for-age (HAZ) <-2 SD below the WHO Child Growth Reference median  *Thinness*: BMI-for-age (BAZ)<-2 SD below the WHO Growth Reference median OR BAZ expressed as <5th percentile of the CDC growth reference  *Chronic energy deficiency*: CED I (Body mass Index (BMI) 17-18.5), CED II (BMI 16-17) and CED III (BMI< 16)  **Overnutrition**  *Overweight*: Overweight: BMI-for-age >+1 SD above the WHO Growth Reference median OR BAZ expressed as <85th percentile of the CDC growth reference OR BMI over or equal to 25kg/m² (International Obesity Task Force - IOTF cut offs) OR BMI over or equal to 23kg/m² (Indian Academy of Paediatrics - IAP)cut offs) OR overweight (WAZ >2SD); obesity (BAZ >2SD)  *Obese*: BMI-for-age >+2 SD above the WHO Growth Reference median OR Obesity: BAZ expressed as <95th percentile of the CDC growth reference OR BMI over or equal to 30kg/m² (IOTF cut offs) OR BMI over or equal to 27kg/m² (IAP cut offs)  **Micronutrient deficiency**  *Anaemia*: Children <12 years (Hb <11.5.0 g/dl); Children 12-14 years and females 15+ years (mild (110-119 g/l), moderate (80-109 g/l), severe (<80)); men 15+ (mild (110-129 g/l), moderate (80-109 g/l), severe (<80g/l) (WHO cut offs)  *Iron deficiency*: Serum Ferritin level <15µg/L (WHO cut offs)  *Vitamin-A deficiency*: Serum retinol concentrations ≤0.70 μmol/L (WHO cut-offs)  *Calcium deficiency*: Serum ascorbic acid level of <0.3mg/100ml (WHO cut-offs).  *Vitamin-D deficiency*: No agreed cut-offs. Individual: deficiency can be classified as <25/ <30/ <50nmol/L and insufficiency <50/ <75nmol/L 25(OH)D level of ≤20 ng/mL (The Endocrine Society classification for vitamin D deficiency)  *Zinc* *deficiency*: For <10 years old: Serum zinc level of <65 ug/dL. (WHO/UNICEF serum zinc status indicators).  *Iodine deficiency*: Median urinary iodine concentration (UIC): insufficient < 100 µg/L, adequate 100-299 µg/L and excessive 300 and above µg/L (WHO cut offs)  *Goitre*: Grade 1 (Persons with neck thickening as a result of enlarged thyroid, palpable goitre, not visible in normal position of neck. Includes nodular goitre also) and Grade 2 (Neck swelling, visible when the neck is in normal position, corresponding to enlarged thyroid found in palpation (WHO definition) |
| --- |
